# Excision in Two Dimensions: Synthesis of 2D Metal–Organic Nanosheets via Clip-off Chemistry

**DOI:** 10.1021/jacs.5c16242

**Published:** 2025-12-08

**Authors:** Pilar Fernández-Seriñán, Partha Samanta, Inhar Imaz, Daniel Maspoch

**Affiliations:** † Catalan Institute of Nanoscience and Nanotechnology (ICN2), CSIC and Barcelona Institute of Science and Technology, Campus UAB, 08193 Bellaterra, Barcelona, Spain; ‡ Departament de Química, Facultat de Ciències, Universitat Autònoma de Barcelona, 08193 Bellaterra, Spain; § ICREA, Pg. Lluís Companys 23, 08010 Barcelona, Spain

## Abstract

Two-dimensional metal–organic nanosheets (MONs) combine the topological and chemical versatility of metal–organic frameworks (MOFs) with the advantages of 2D materials, yet their top-down synthesis remains limited mainly to systems with labile coordination bonds. Here, we report a top-down strategy to access MONs from robust 3D MOFs via in-plane covalent bond excision using clip-off chemistry. This approach enables formation of crystalline, porous 2D layers from otherwise nonexfoliable 3D Zr-polycarboxylate frameworks, expanding the scope of accessible MON architectures. Compared to the parent MOF, the resulting MONs exhibit enhanced catalytic performance in esterification reactions, due to the improved exposure of outer active sites.

## Graphical abstract

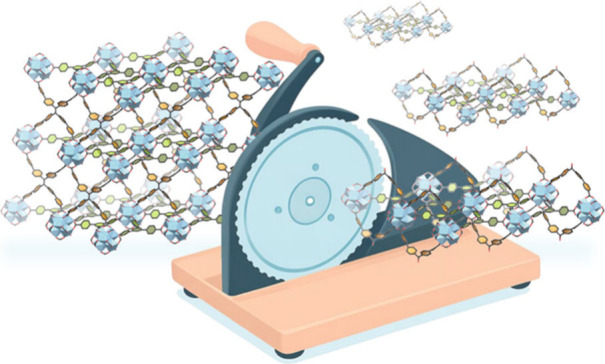

Two-dimensional (2D) materials have attracted widespread attention due to their exceptional physical and chemical properties. Among them, organic materials such as graphene, and inorganic materials such as transition-metal dichalcogenides, hexagonal boron nitride, or MXenes, have emerged as platforms for myriad applications, including in electronics,^1–3^ energy storage and conversion,^4–7^ catalysis,^8,9^ sensing,^10–12^ and membrane technologies.^13–15^ Confining matter to two dimensions while preserving long-range order and achieving high surface-to-volume ratios has positioned 2D materials at the forefront of nanotechnology and materials science.

Miniaturization of metal–organic frameworks (MOFs) into 2D architectures known as metal–organic nanosheets or nanolayers (MONs) is rapidly gaining attention.^16,17^ This emerging class of hybrid materials combines the structural and chemical versatility of MOFs with the advantages of low dimensionality, opening opportunities in separations,^18^ sensing,^19^ catalysis,^20–22^ and optoelectronics.^23^ For instance, ultrathin MONs in mixed-matrix membranes show excellent separation performance due to improved substrate diffusion,^24^ while exfoliated ZIF-L nanosheets outperform their bulk counterparts in catalysis, due to greater exposure of active sites.^25^

Currently, MONs can be synthesized bottom-up or top-down. In the former, MONs are assembled during synthesis, typically by controlling metal–ligand coordination geometry, introducing modulators, or using templates that limit growth in the lateral dimension.^26–30^ However, these methods often require complex reaction conditions, are difficult to generalize, or face challenges with template removal. In contrast, top-down approaches involve exfoliating layered bulk MOFs or breaking down 3D frameworks into 2D fragments through external stimuli, such as by ultrasonication.^31^ This requires structurally labile or weakly bonded planes that can be preferentially cleaved while preserving their lateral dimensions. Such planes are usually found in 3D MOFs with carboxylate-based layers connected by labile pillars (e.g., pyridyl-based linkers),^32–34^ or in defect-engineered structures such as cluster-condensed frameworks.^35^ However, in these cases, the formation of MONs through exfoliation is limited to frameworks that enable cleavage of metal–linker bonds, thus excluding the most common 3D MOFs with strong metal–carboxylate bonds along all crystallographic directions.

Herein, we report a new top-down synthetic route to MONs, based on our group’s expanding concept of clip-off chemistry,^36–38^ and validate their catalytic activity in organic transformations. In this approach, covalent bonds within the polycarboxylate linkers of 3D MOFs are selectively cleaved, breaking them down into 2D fragments that can then be isolated via sonication as crystalline MONs.

To validate our approach, we chose LIMF-66W^39^ as the parent 3D MOF, as it is constructed from Zr-clusters connected by polycarboxylate linkers in all three dimensions. It can be described as comprising **kgd**-type layers formed by 10-connected Zr_6_O_8_ nodes joined together by the tritopic linker 1,3,5- tris­(4-carboxyphenyl)­benzene (H_3_BTB). These layers are further bridged along the *c*-axis of the crystal by the tetratopic linker 4′,4′′′,4′′′′′,4′′′′′′′-(ethene-1,1,2,2-tetrayl)­tetrakis­([1,1′-biphenyl]-4-carboxylic acid) (H_4_ETTC). In this configuration, the cleavable olefinic bonds within the ETTC^4–^ linkers are all aligned parallel to the *c* crystallographic axis, thereby enabling the selective release of the layers upon cleavage of the olefinic bonds along this direction (Figure 1).

**Figure 1:**
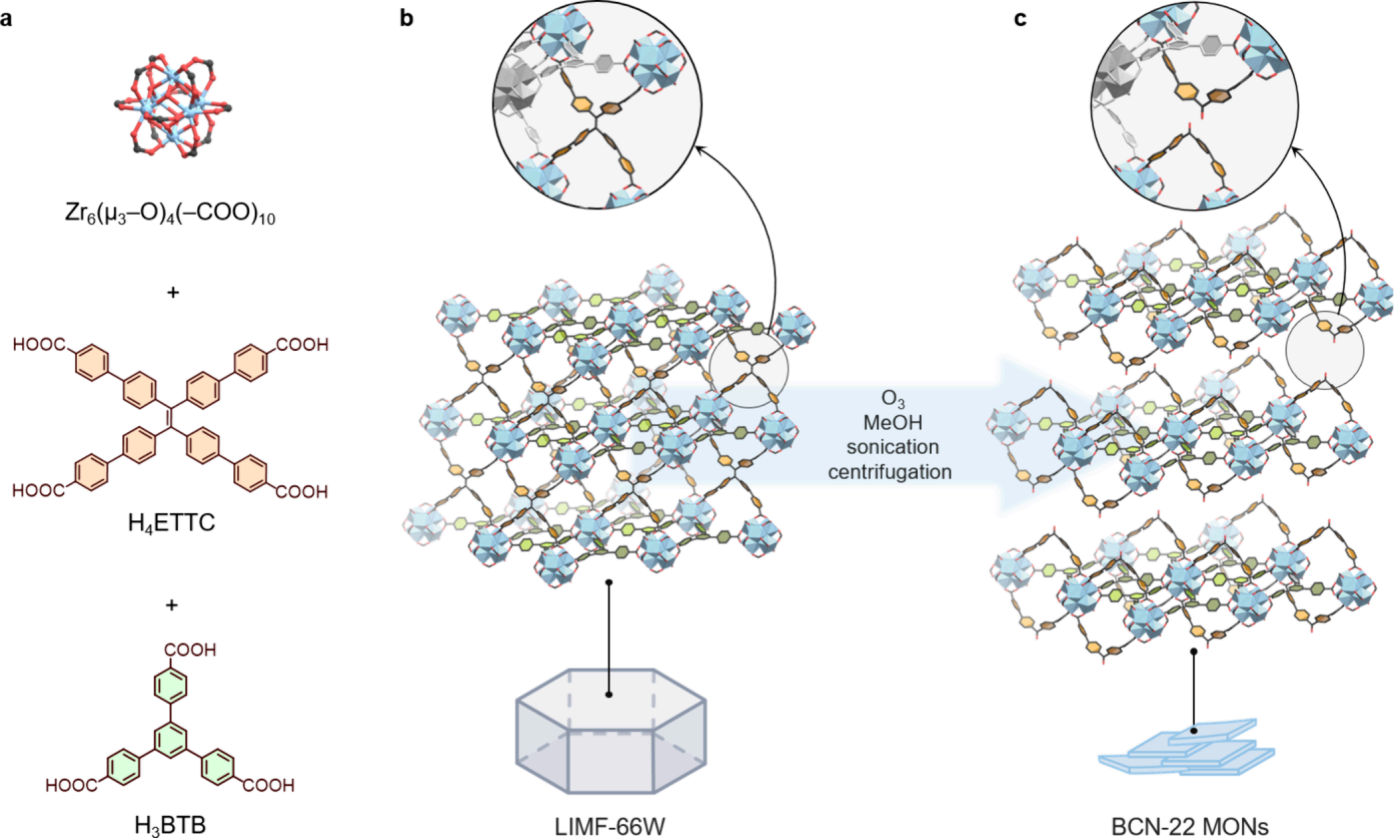
Schematic of the synthesis of MONs via clip-off chemistry from a 3D MOF: (a) the components of the parent MOF LIMF-66W; (b) cleavable sites (top) within LIMF-66W (middle) and its crystal habit (bottom); and (c) cleaved H_2_CBC (top) in the structure of BCN-22 (middle) within the obtained MONs (bottom).

To demonstrate the feasibility of disconnecting layers in a 3D MOF via cleavage of covalent bonds, we first confirmed that the central quaternary olefinic bonds of H_4_ETTC could be selectively cleaved into two ketone groups upon exposure to ozone,^40^ which yields 4′,4′′′-carbonylbis­(([1,1′-biphenyl]-4-carboxylic acid)) (H_2_CBC) (Scheme S2). To this end, a stream of ozone (∼40 g Nm^–3^) was bubbled through a methanolic suspension of H_4_ETTC for 5 h. Next, a white solid was isolated by filtration and identified as H_2_CBC, using proton and carbon nuclear magnetic resonance (^1^H NMR and ^13^C NMR) spectroscopy, as well as electrospray ionization mass spectrometry (ESI-MS) (Figures S2–S4).

Having confirmed selective cleavage of the olefinic bonds in H_4_ETTC, we synthesized the precursor MOF, LIMF-66W, as transparent hexagonal prismatic single crystals (Figure S5).^39^ Approximately 10 mg of these LIMF-66W crystals were packed into a plastic tube, and then subjected to ozonolysis for 5, 30, 60, or 90 min using a dry ozone stream (∼25 g Nm^–3^). After each ozonation period, the resultant crystals were removed and analyzed by ^1^H NMR spectroscopy after digestion, ESI-MS and Fourier-transform infrared spectroscopy (FT-IR). The ^1^H NMR spectra revealed that although the signals corresponding to H_3_BTB remained unchanged, the initial resonances associated with H_4_ETTC (at 7.21, 7.64, 7.78, and 7.98 ppm; see Figure S6) gradually decreased as the ozonolysis progressed. Concurrently, new aromatic signals appear at 7.92, 7.93, 7.98, and 8.09 ppm, consistent with the formation of H_2_CBC. In the sample subjected to 90 min of ozonolysis, no traces of H_4_ETTC were detected, indicating complete cleavage (see Figures 2a and 2b, as well as Figure S7). This fully converted sample corresponds to a 2D-MOF (hereafter called BCN-22) composed of Zr_6_O_8_ clusters linked by CBC^2–^ and unreacted BTB^3–^ linkers. The chemical transformation was further corroborated by ESI-MS analyses of both pristine and ozonized samples, which indicated H_3_BTB in both LIMF-66W and BCN-22, but H_2_CBC only in BCN-22 (Figure S8). Finally, the presence of the ketone-functionalized H_2_CBC was further supported by FT-IR, as the spectrum for BCN-22 featured a characteristic C=O stretching band at 1687 cm^–1^ (Figure S9).

**Figure 2:**
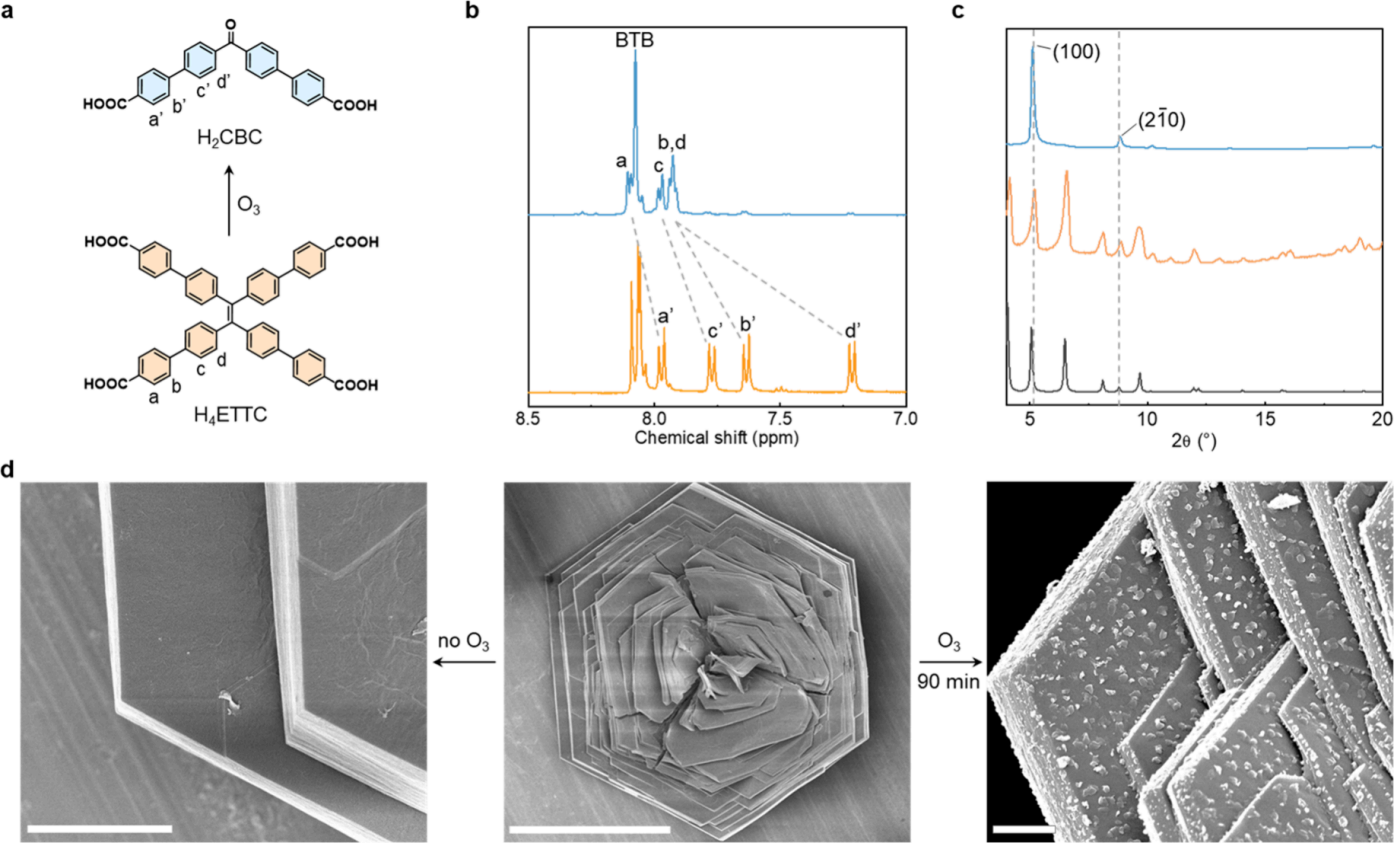
(a) Ozonolysis of H_4_ETTC to produce H_2_CBC. (b) ^1^H NMR spectra of the digested LIMF-66W (orange) and of the ozonized 2D-MOF BCN-22 (blue). (c) PXRD pattern of the calculated LIMF-66W (black, bottom), as synthesized LIMF-66W (orange, middle) and BCN-22 (blue, top). (d) SEM micrograph of the surface of the parent LIMF-66W (left) and its overall morphology (middle), and effect of the ozonolysis in the surface of the crystal (right). Scale bars = 50 μm (middle), 3 μm (sides).

Given that the alkene bonds from ETTC^4–^ are aligned parallel to the *c* crystallographic axis in LIMF-66W, we reasoned that the progress of the ozonolysis reaction could also be monitored via powder X-ray diffraction (PXRD) spectroscopy. The PXRD pattern of pristine LIMF-66W matched the reported one,^39^ confirming its successful synthesis and phase purity. As the ozonolysis reaction selectively cleaves the alkene bonds, the layers within LIMF-66W progressively disconnect, leading to a gradual attenuation of the (*hkc*) reflections in the PXRD patterns, whereas the (*hk*0) reflections are preserved (Figure S10). When the 2D BCN-22 is formed, only the sharp (100) peak at 2θ = 5.1° and the weaker (21̅0) reflection at 2θ = 8.8° remain visible (Figure 2c). This PXRD signature indicates that BCN-22 retains the periodicity within its layers while losing the long-range order along the stacking direction. Notably, BCN-22 is isoreticular with the Zr-BTB nanosheets previously reported by Matzger and co-workers,^41^ as corroborated by their matching PXRD patterns.

Interestingly, the chemical disconnection of the 3D framework into layers was accompanied by morphologic changes in the pristine microscale (∼50 μm) hexagonal crystals of LIMF-66W (Figure 2d). Scanning electron microscopy (SEM) revealed the appearance of flakes on the initially smooth crystal surfaces as the ozonolysis progressed. These flakes preferentially formed on the upper and lower crystal faces, which correspond to the (001) and (001̅) planes, respectively (Figure S11). This suggests that flaking might be guided by the cleavage along the *c* crystallographic axis.

Next, we attempted to exfoliate the flakes from the crystals by immersing and sonicating the ozonized crystals in MeOH. However, although this produced some observable isolated parallelepiped nanosheets of BCN-22, in extremely low yield, they were heterogeneous in size, with layer thicknesses of <200 nm and lateral dimensions of <2 μm (Figure S12).

We then endeavored to improve the synthesis of BCN-22 nanosheets by facilitating the separation of the exfoliated layers, reasoning this could be achieved by chemically disconnecting the 3D framework directly in MeOH. For this, 10 mg of LIMF-66W crystals were dispersed in 2 mL of MeOH, and dry ozone (∼25 g Nm^–3^) was bubbled for 90 min. Following ozonolysis, mild sonication and purification by centrifugation yielded a colloidal suspension of BCN-22 nanosheets (yield: 30%), which exhibited the characteristic Tyndall effect (Figure 3a, Figure S13). The formation of BCN-22 was confirmed by ^1^H NMR, PXRD, and X-ray photoelectron spectroscopy (XPS) (Figure S14). Interestingly, reactions on 10-mg and 100-mg scales gave similar purities and yields. Control experiments, in which LIMF-66W was incubated in MeOH without ozonolysis and then sonicated, led to the milling of LIMF-66W crystals, without any sign of corresponding MONs (Figure S15).

**Figure 3:**
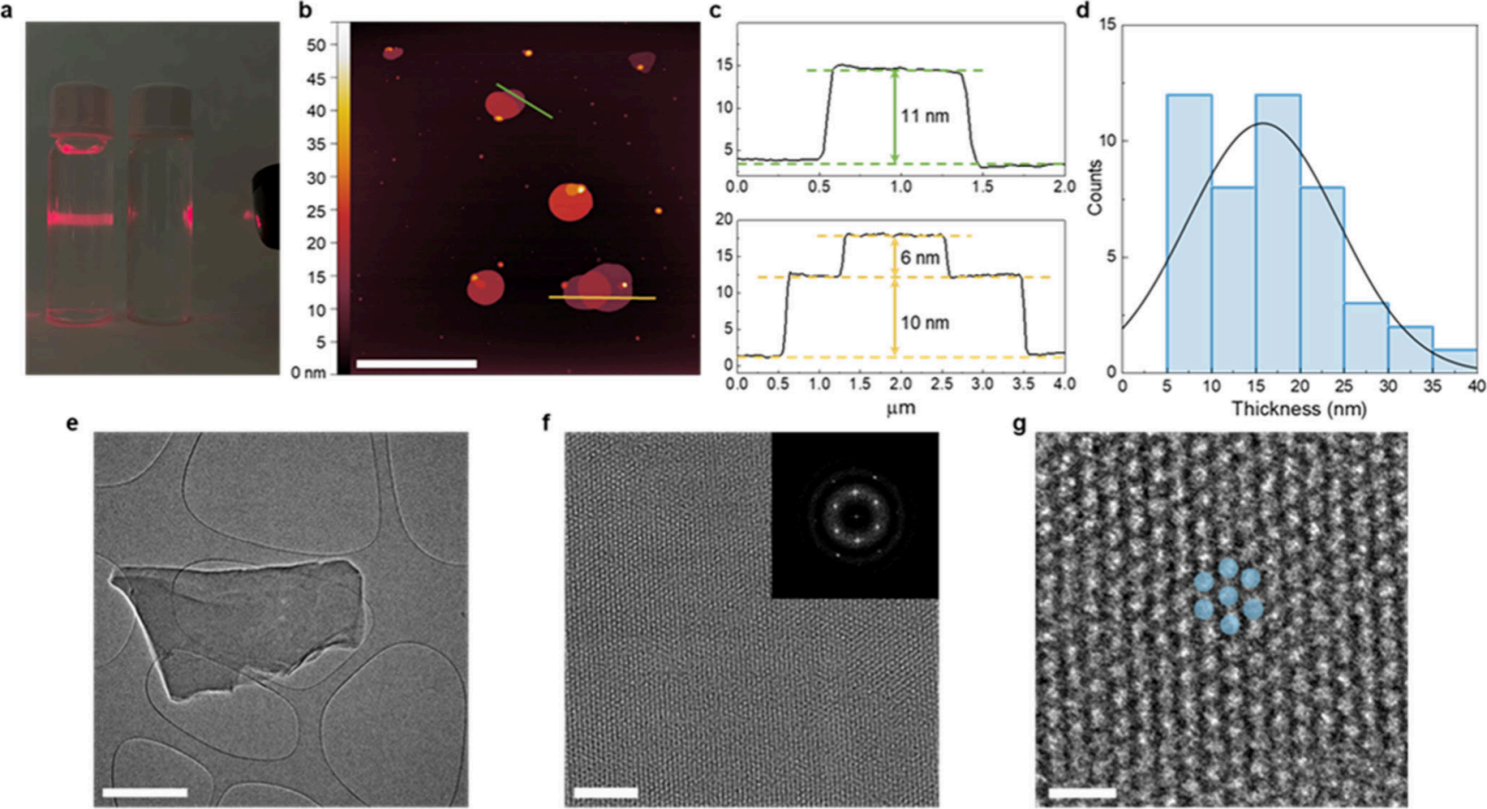
(a) Tyndall effect of BCN-22 dispersed in MeOH (left vial), and blank MeOH for reference (right vial). (b) AFM image of BCN-22 nanosheets with regions of interest marked in green and yellow (scale bar = 5 μm), and (c) corresponding height profiles. (d) Statistical distribution of all height profiles measured. (e) HR-TEM micrograph of a single BCN-22 nanosheet. (f) HR-TEM micrograph of the surface of a BCN-22 nanosheet. Inset shows the Fourier transform. (g) Magnified HR-TEM micrograph including a representation (in blue) of the hexagonal array. Scale bars = 1 μm (panel (e)), 20 nm (panel (f)), 5 nm (panel (g)).

To structurally characterize the BCN-22 nanosheets, we next turned to atomic force microscopy (AFM) and high-resolution transmission electron microscopy (HR-TEM). AFM revealed that the obtained MONs have an average layer thickness of ∼15 nm, corresponding to approximately 6 monolayers of BCN-22, and lateral dimensions of ∼2 μm (see Figures 3b–d, as well as Figure S16). Crucially, HR-TEM enabled direct visualization of these individual crystalline MONs (see Figure 3e, as well as Figure S17), for measurements on crystal lattice *d*(100) spacing, which was found to be 1.70 nm, and *d*(21̅0), found to be 0.98 nm, closely matching the calculated values of 1.71 and 1.00 nm, respectively (Figure 3f). Moreover, HR-TEM allowed for clear identification of the hexagonal array expected in BCN-22 (Figure 3g). Additionally, N_2_ sorption measurements revealed a Brunauer–Emmett–Teller (BET) surface area of 155 m^2^/g, significantly lower than that of the precursor MOF, which is consistent with the loss of interlayer porosity (Figures S18–S22). This loss was further corroborated through pore-size distribution measurements, which revealed far fewer interlayer pores of ∼18 Å, compared to inside the precursor material (Figure S23).

Having converted the Zr–polycarboxylate MOF LIMF-66W into BCN-22 nanosheets, we then investigated their catalytic potential. We expected that the nanosheets would expose a significantly larger number of catalytically active Zr clusters on their outer surface compared to the parent MOF. As a model reaction, we selected esterification of carboxylic acids using alcohols, choosing oleic acid as representative due to the widespread use of its ester derivatives in the pharmaceutical, cosmetic, and polymer industries.^42^ As a starting point, we targeted the synthesis of methyl oleate from oleic acid and methanol, using BCN-22 as catalyst, under solvent-free conditions. The reaction was run using 1 mmol of oleic acid and 8 mmol of methanol, in the presence of BCN-22 (2.0 mol % Zr) at 100 °C for 6 and 12 h, affording yields of 57% and 98%, respectively.

To assess the catalytic advantage conferred by the 2D nature of BCN-22, we compared its performance with that of its parent 3D MOF, LIMF-66W, under identical conditions. LIMF-66W exhibited markedly lower activity, with yields of only 30% after 6 h and 47% after 12 h. Moreover, a blank reaction (i.e., no catalyst) run for 12 h gave an even worse yield (27%), confirming both catalysts’ activity. Encouraged by the superior activity of BCN-22, we extended the substrate scope to include other linear alcohols: ethanol, 1-propanol, 1-butanol, and 1-hexanol, which afforded the corresponding esters in yields of 61%, 53%, 52%, and 50%, respectively, after 12 h (Figure 4). In all these cases, LIMF-66W was much less active, whereas control reactions without catalyst showed negligible conversion. We also tested the reactivity with bulkier alcohols (benzyl alcohol and 2,2-dimethyl-1-propanol), again observing superior performance for BCN-22, compared to LIMF-66W (Tables S1 and S2). Finally, for the esterification of oleic acid to ethyl oleate, both BCN-22 and LIMF-66W maintained their catalytic efficiency over five consecutive cycles, with no substantial loss in activity (Figures S66 and S67). Consistent with this preserved functionality, PXRD and inductively coupled plasma optical emission spectroscopy (ICP-OES) confirmed the structural integrity of the solids after catalysis (Figure S68, Table S3).

**Figure 4:**
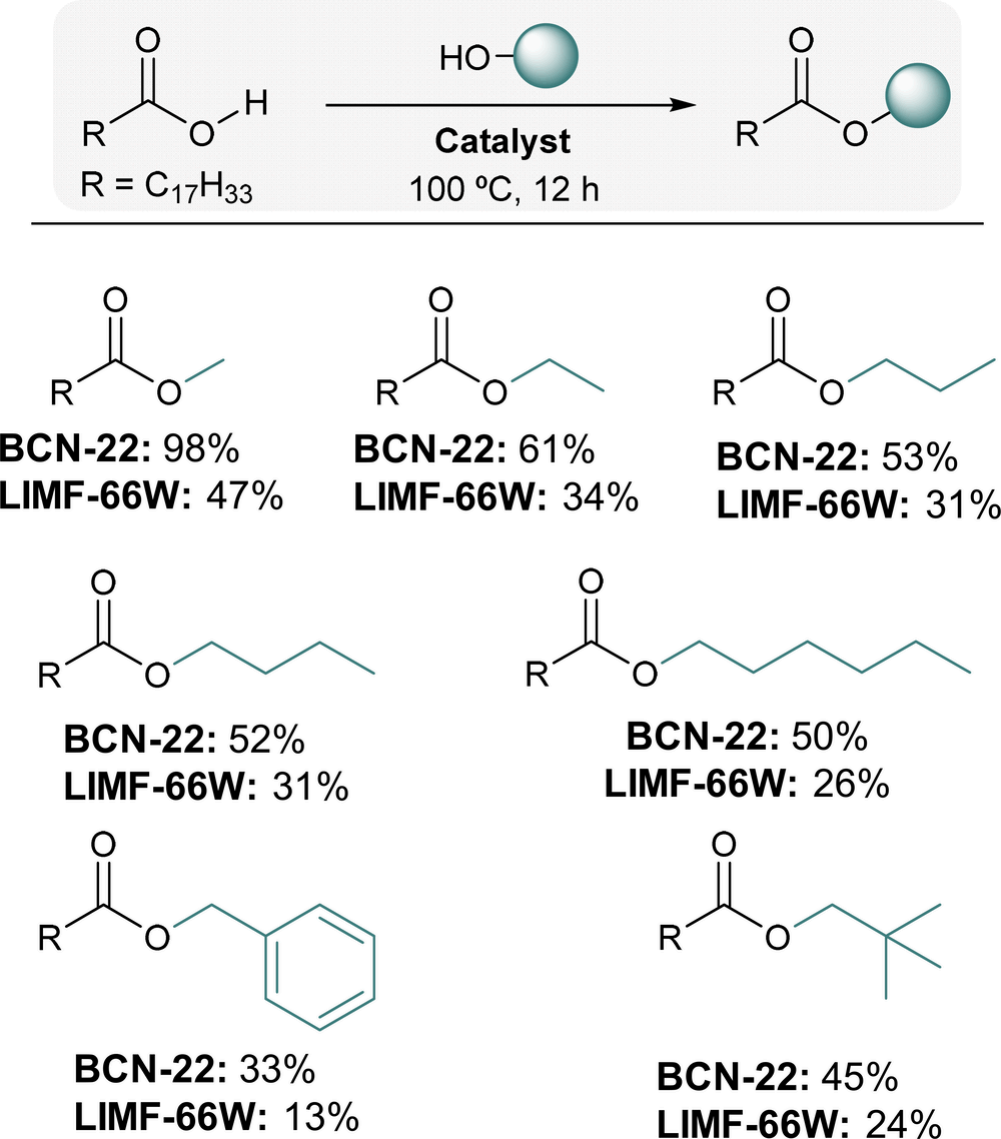
Substrate scope for esterification of oleic acid with alcohols, as catalyzed by either BCN-22 or LIMF-66W (2.0 mol % Zr).

In conclusion, we have shown that 3D MOFs can serve as precursors for the controlled synthesis of 2D MONs via clip-off chemistry. By strategically incorporating cleavable linkers along a crystallographic plane, and then selectively excising covalent bonds within those linkers, we were able to form and isolate crystalline MONs whose catalytic activity exceeded that of the parent MOF. As a new, facile synthetic route to previously inaccessible MONs, our approach both expands the scope of clip-off chemistry and broadens the design space for tailored 2D MOF materials.

## Supplementary Material



## Notes

Detailed experimental section and characterization including NMR spectra, PXRD patterns, ESI-MS and FT-IR data, SEM images, BET and pore size distribution data (PDF)
