# Supplementary material for: Excision in Two Dimensions: Synthesis of 2D Metal–Organic Nanosheets via Clip-off Chemistry
Source: J Am Chem Soc. 2025 Dec 8;147(50):45849–54. doi: 10.1021/jacs.5c16242 (PMC12715795; doi:10.1021/jacs.5c16242)
Supplement: Supplementary file 1 [file ja5c16242_si_001.pdf]

Supporting Information for

Excision into Two Dimensions: Synthesis of 2D Metal-Organic Nanosheets via Clip-off Chemistry

Pilar Fernández-Seriñán,[a,b] Partha Samanta,[a,b] Inhar Imaz,[a,b,*] Daniel Maspoch[a,b,c,*]

[a] Catalan Institute of Nanoscience and Nanotechnology (ICN2), CSIC, and Barcelona Institute of Science and Technology, Campus UAB, 08193 Bellaterra, Barcelona, Spain

[b] Departament de Química, Facultat de Ciències, Universitat Autònoma de Barcelona, 08193 Bellaterra, Spain

[c] ICREA, Pg. Lluís Companys 23, 08010 Barcelona, Spain

* To whom correspondence may be addressed: e-mail: inhar.imaz@icn2.cat; daniel.maspoch@icn2.cat

Table of Contents

Section S1. General methods and materials ... 3

S1.1 Chemicals and reagents ... 3

S1.2 Instruments ... 3

Section S2. Synthesis of 4',4''',4''''',4'''''''-(ethene-1,1,2,2-tetrayl)tetrakis(([1,1'-biphenyl]-4-carboxylic acid)) (H_4ETTC) ... 5

Section S3. Synthesis of 4',4'''-carbonylbis(([1,1'-biphenyl]-4-carboxylic acid)) (H_2CBC) ... 8

Section S4. Synthesis of LIMF-66W ... 12

S4.1. NMR spectroscopy ... 12

Section S5. Synthesis of BCN-22 via solid-gas ozonolysis ... 13

S5.1. NMR spectroscopy ... 13

S5.2. ESI-MS ... 15

S5.3. FT-IR ... 16

S5.4. PXRD ... 17

S5.5. Scanning Electron Microscopy (SEM) ... 18

Section S6. Synthesis of BCN-22 via ozonolysis in dispersion ... 20

S6.1. Isolation of nanosheets of BCN-22 ... 20

S6.2. Tyndall effect ... 21

S6.3. Formation of BCN-22 nanosheets ... 22

S6.4. Scanning Electron Microscopy (SEM) ... 23

S6.6. Atomic Force Microscopy ... 24

S6.6. Transmission Electron Microscopy (TEM) ... 25

S6.7. BET and Pore Size Distribution measurements ... 26

Section S7. Catalytic experiment ... 32

S7.1. General protocol ... 32

S7.2. Recyclability experiments ... 57

S7.3. PXRD after catalytic experiments ... 59

S7.4. ICP-OES ... 60

S7.5. Hot-filtration experiments ... 61

References ... 62

Section S1. General methods and materials

S1.1 Chemicals and reagents

Potassium carbonate, potassium hydroxide, tetrakis(triphenylphosphine)palladium(0) ($Pd(PPh_3)_4$) and zirconium(IV) oxide chloride octahydrate were purchased from Sigma Aldrich. 1,3,5-tris(4-carboxyphenyl)benzene (H_3BTB) was purchased from BLD pharm. *N,N*-dimethylformamide (DMF), tetrahydrofuran (THF), methanol (MeOH), dichloromethane (DCM) and concentrated hydrochloric acid (conc. HCl 37%) were purchased from Fisher Scientific. Trifluoroacetic acid (TFA) was purchased from TCI. Deuterated dimethyl sulfoxide-d_6 (DMSO-d_6) and $CDCl_3$ were purchased from Eurisotop. All reagents and solvents were used as received without further purification. The deionized water from all the aqueous solutions in the article was obtained from a Milli-Q® system (18.2 MΩ·cm).

S1.2 Instruments

Powder X-ray diffraction (PXRD) data was recorded on an X'Pert PRO MPD analytical diffractometer (Panalytical) at 45 kV, 40 mA using CuKα radiation (λ = 1.5418 Å). X-ray powder diffraction patterns of BCN-22 were collected using a capillary of 0.7 mm inner diameter.

Proton Nuclear Magnetic Resonance (^{1}H-NMR) spectra were collected in a Bruker Avance NEO 300 MHz, Bruker Avance NEO 400 MHz spectrometer and Bruker Avance 500 NMR spectrometer at "Servei de Ressonància Magnètica Nuclear" from Autonomous University of Barcelona (UAB). All MOFs digestions were performed in HF (5% in aqueous solution) and heated at 120 ºC overnight.

Fourier Transform Infrared (FT-IR) spectra were acquired on a Bruker IR Alpha II spectrometer equipped with a diamond attenuated total reflection (ATR) accessory. All spectra were collected neat in ambient atmosphere.

Ozonolysis was carried out using an ozone generator GHBZO3-E Commercial Ozone Generator from ZonoSistem, equipped with ozone analyzer UVOZ-1200.

N_2 sorption isotherms were collected using an ASAP 2460 (Micromeritics). Temperature was controlled by using a liquid nitrogen bath (77 K). Pore size distribution was estimated using a density functional theory (DFT) model (DFT – slit – N_2–DFT model) implemented in the Microactive 4.06 software with a regularization factor of 0.01.

Elemental Analysis (EA) measurements of C, H and N were performed in a Flash EA2000

Thermo Fisher Scientific analyzer at “Servei d'Anàlisi Química (SAQ)” from Autonomous University of Barcelona (UAB).

Electrospray ionization mass spectrometry (ESI-MS) measurements were performed using a 1260 Infinity II system coupled to a 6420 Triple Quad MS, Agilent Technologies mass spectrometer at “Servei d'Anàlisi Química (SAQ)” from Autonomous University of Barcelona (UAB).

Scanning Electron Microscopy (SEM) images were obtained with a FEI Magellan 400L XHR microscope by drop casting the sample on conductive aluminum tape. Samples were coated with Au using a Emitech K550X/K250 C cathodic metallizer.

High-Resolution Transmission Electron Microscopy (HR-TEM) and atomic-resolution High-Angle Annular Dark Field Scanning Transmission Electron Microscopy (HAADF-STEM) images were acquired using a ThermoFisher Scientific Spectra 300 STEM with double-aberration correction, operated at 300 kV. A Thermo Scientific Ceta™ 16M Camera was utilized to acquire HR-TEM images.

Atomic Force Microscopy (AFM) images were collected on an Agilent 550 AFM/SPN microscope. Samples were prepared by diluting 100 µL of BCN-22 ozonolysis crude in 2 mL of MeOH and sonicating for 10 minutes at 90% power. Then, samples were drop casted on a silicon wafer and air dried. 46 images were collected to elaborate statistics on lateral size measurements.

X-ray photoelectron Spectroscopy (XPS) measurements were performed in ultrahigh vacuum of 5 x 1E-10 mbar with a SPECS Phoibos 150 hemispherical analyser using monochromatic Al Kα radiation with an energy of 1486.6 eV. All XPS spectra were calibrated by graphitic carbon 1s at 284.8 eV and analyzed using CasaXPS (Casa Software Ltd.) peak fitting software. Zirconium 3d spectra (Zr 3d) were numerically fitted with Gaussian-broadened Lorentzian peaks (GL(20)).

Inductively Coupled Plasma Optical Emission Spectroscopy (ICP-OES) analyses were performed on an Agilent 5900 at the “Servei d'Anàlisi Química (SAQ)” from Autonomous University of Barcelona (UAB).

Sonication of samples was performed in a VCX 130 Vibra-Cell Ultrasonic Liquid Processor.

Section S2. Synthesis of 4',4''',4''''',4'''''''-(ethene-1,1,2,2-tetrayl)tetrakis(([1,1'-biphenyl]-4-carboxylic acid)) (H_4ETTC)

The tetratopic linker, 4',4''',4''''',4'''''''-(ethene-1,1,2,2-tetrayl)tetrakis(([1,1'-biphenyl]-4-carboxylic acid)) (H_4ETTC), was synthesized according to previous literature reports (Scheme S1).[2]

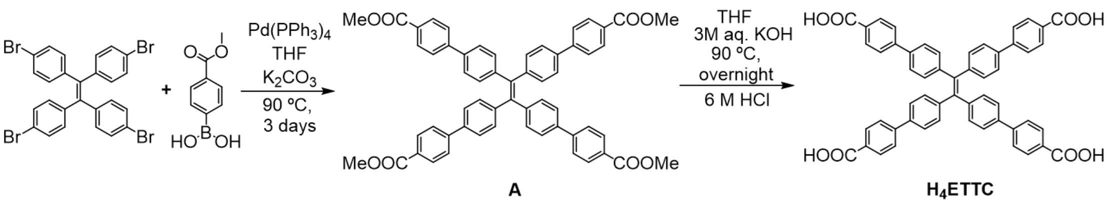

Scheme S1. Synthesis of 4',4''',4''''',4'''''''-(ethene-1,1,2,2-tetrayl)tetrakis(([1,1'-biphenyl]-4-carboxylic acid)) linker.

Preparation of tetramethyl 4',4''',4''''',4'''''''-(ethene-1,1,2,2-tetrayl)tetrakis([1,1'-biphenyl]-4-carboxylate) (A). A solution tetrakis(4-bromophenyl)ethylene (2.0 g, 3.08 mmol) and 4-methoxycarbonyl-benzene boronic acid (3.33 g, 18.5 mmol) in 100 mL THF was taken in a 250 mL round bottom flask and deaerated with argon (3-4 cycles of vacuum and argon). 0.175 g of tetrakis(triphenylphosphine)palladium(0) ($Pd(PPh_3)_4$) was added to the reaction mixture. Then, 15 mL of 3M K_2CO_3 solution in water was added to the reaction mixture and deaerated again with argon (one cycle). The reaction mixture was allowed to reflux under argon atmosphere for 48 hours at 90 °C. After completion of the reaction, THF was removed under reduced pressure and then, the reaction mixture was extracted with dichloromethane (DCM) and aqueous sat. NaCl solution. A yellowish green solid was obtained after removing the DCM under reduced pressure, which was purified with column chromatography to produce A as a yellowish green colored solid. Yield: 60 %.

Preparation of 4',4''',4''''',4'''''''-(ethene-1,1,2,2-tetrayl)tetrakis(([1,1'-biphenyl]-4-carboxylic acid)) (H_4ETTC). In a 250 mL round bottom flask, 2.0 g (0.025 mol) of product A

was dispersed in 35 mL THF and 35 mL of 3M aqueous solution of KOH. Then, the reaction mixture was refluxed at 90 ºC for 18 hours (overnight). On completion of the reaction, the solution was concentrated under reduced pressure and then, diluted with water. Further, the clear solution was acidified with 6 M HCl to produce a yellowish green precipitate. Upon filtration, a light green colored product was obtained, which was washed with water to remove access acid and then, H_4ETTC was dried. Yield: 90 %. ^{1}H-NMR (400 MHz, DMSO-d_6) of H_4ETTC: δ (ppm) = 7.98 (d, J = 8.5 Hz, 1H), 7.78 (d, J = 8.6 Hz, 1H), 7.63 (d, J = 8.5 Hz, 1H), 7.21 (d, J = 8.4 Hz, 1H).

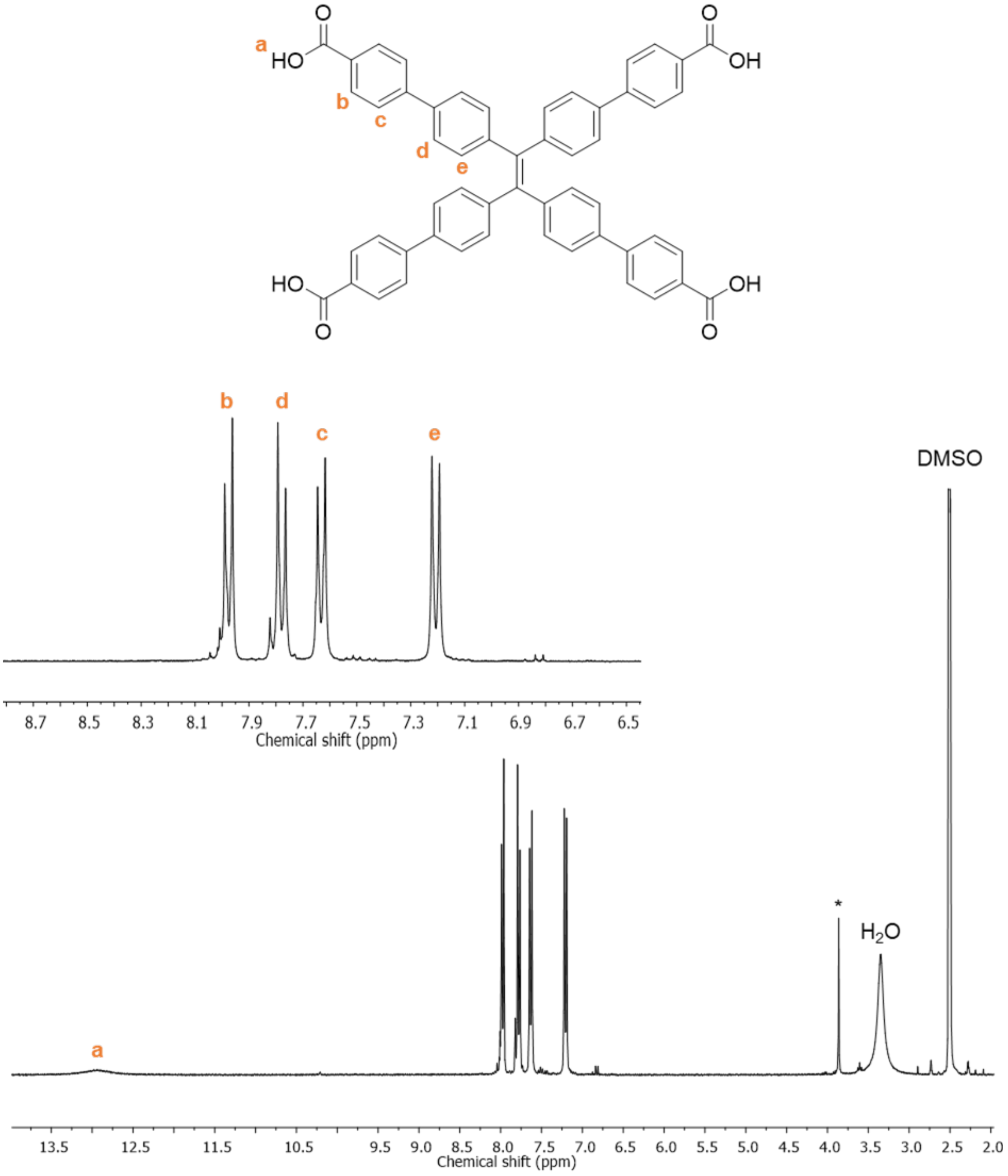

Figure S1. ^{1}H-NMR spectrum (400 MHz, DMSO-d_6) of H_4ETTC.

Section S3. Synthesis of 4',4'''-carbonylbis(([1,1'-biphenyl]-4-carboxylic acid)) (H_2CBC)

The ditopic linker 4',4'''-carbonylbis(([1,1'-biphenyl]-4-carboxylic acid)) (H_2CBC) was synthesized by means of an ozonolysis reaction (Scheme S2).

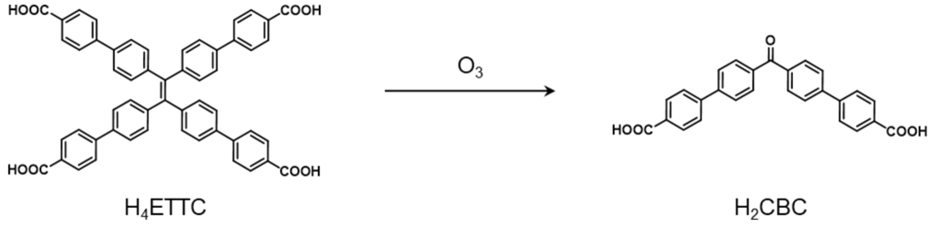

Scheme S2. Synthesis of the linker 4',4'''-carbonylbis(([1,1'-biphenyl]-4-carboxylic acid)).

To that effect, 20 mg of 4',4''',4''''',4'''''''-(ethene-1,1,2,2-tetrayl)tetrakis(([1,1'-biphenyl]-4-carboxylic acid)) (H_4ETTC) were suspended in 50 mL MeOH in a 100 mL round bottom flask sealed with a septum. Two needles were used, one as an outlet and one as an inlet connected to the ozonator setup. Then, dry ozone was flowed (~40 g Nm^{-3}) continuously through the sample for 5 hours, resulting in the obtention of a white powder of H_2CBC. Yield: 100 %. ^1H-NMR (400 MHz, DMSO-d_6) of H_2CBC: δ (ppm) = 13.05 (s), 8.14 – 8.09 (m), 7.98 (d, J = 6.2 Hz), 7.93 (d, J = 5.0 Hz), 7.91 (s).

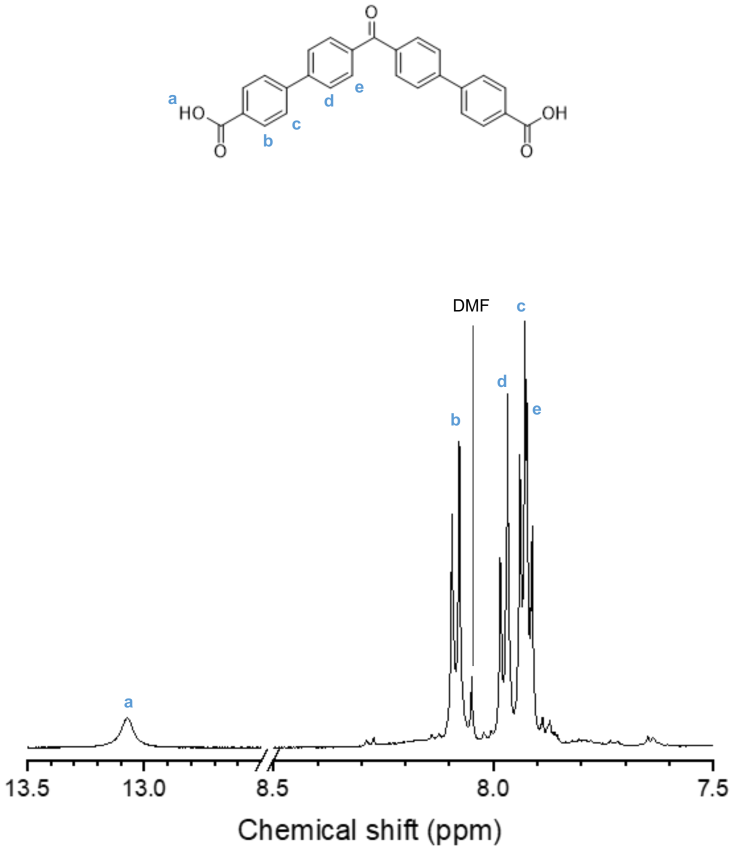

Figure S2. ^{1}H-NMR spectrum (400 MHz, DMSO-d_6) of H_2CBC.

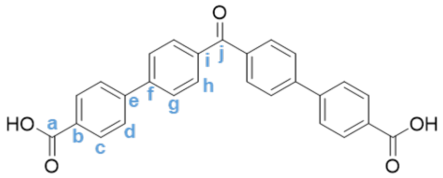

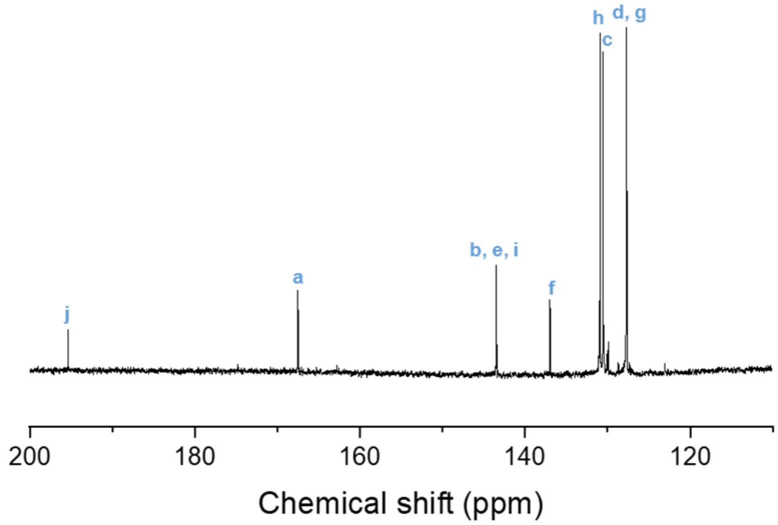

Figure S3. ^{13}C-NMR spectrum (400 MHz, DMSO-d_6) of H_2CBC.

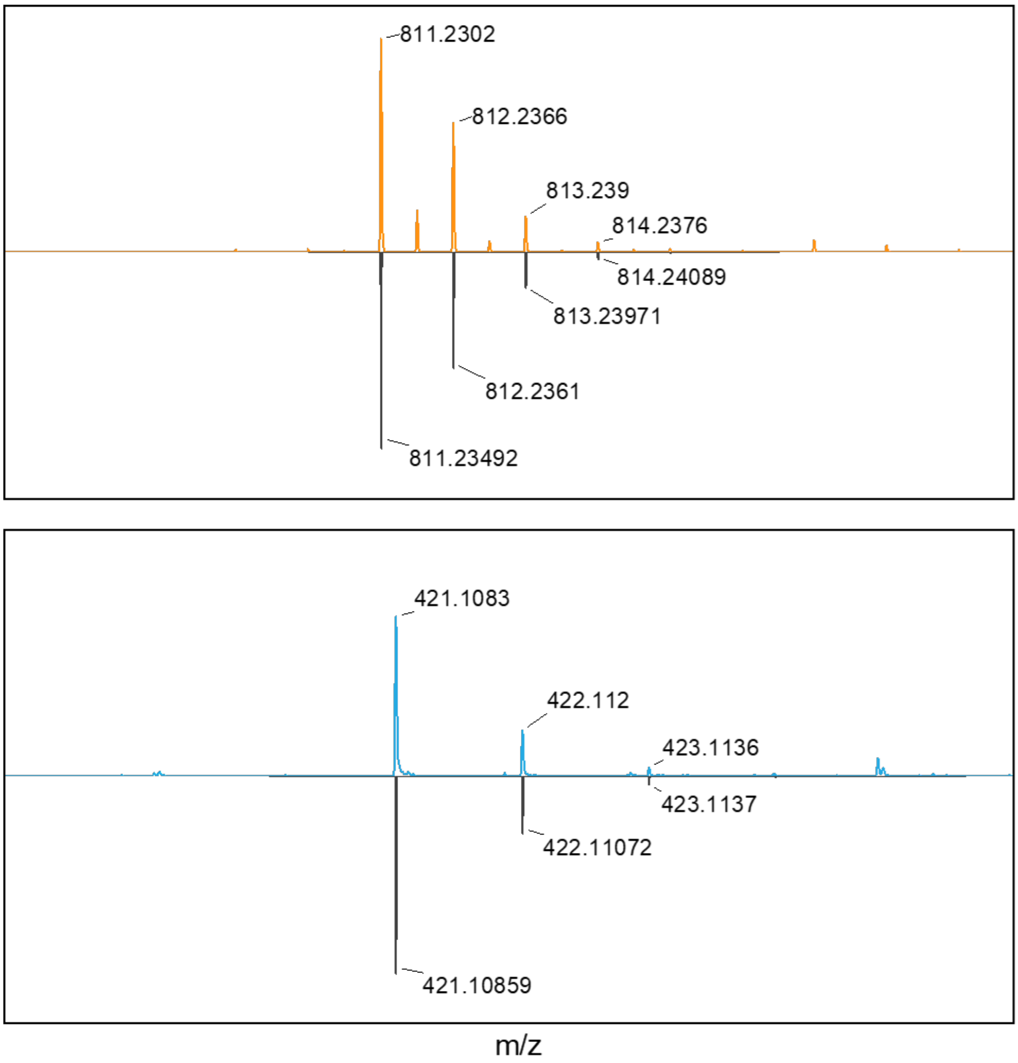

Figure S4. ESI-MS spectrum of H_4ETTC (top, experimental data in orange, calculated in grey) and H_2CBC (bottom, experimental data in blue, calculated in grey).

Section S4. Synthesis of LIMF-66W

LIMF-66W was synthesized according to the reported protocol in literature[3]. Elemental analysis found (calculated) for $(C_{108}H_{62}O_{32}Zr_6)\cdot(DMF)_{0.5}\cdot(H_2O)_6$, which represents $C_{113}H_{79}O_{40}Zr_6$: C% 51.15 (51.3), H% 3.49 (3.05), N% 0.29 (0.27).

S4.1. NMR spectroscopy

10 mg of BCN-22 were first digested using an aqueous solution (200 μL) of 5 wt% HF and heated at 120 °C overnight. Afterwards, 500 μL of DMSO-d_6 was directly added to the resulting solution.

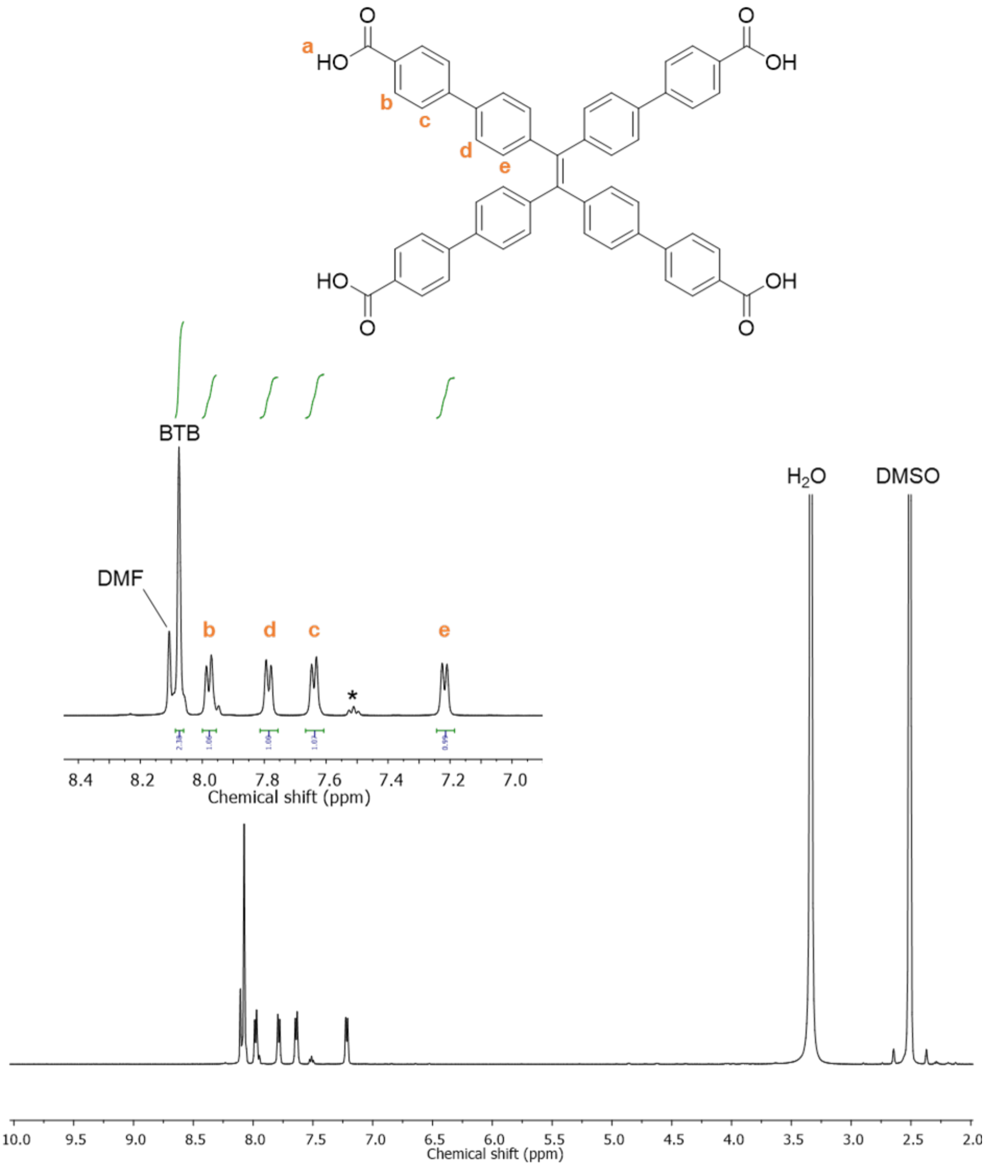

Figure S5. ^{1}H-NMR spectrum (400 MHz) of digested LIMF-66W, displaying the ratio between BTB^{3-} and $ETTC^{4-}$. Note that the experimental ratio of BTB^{3-} over $ETTC^{4-}$ is 2:1, matching the expected one.

Section S5. Synthesis of BCN-22 via solid-gas ozonolysis

BCN-22 was obtained through a solid-gas ozonolysis reaction. Briefly, 10 mg of LIMF-66W, previously soaked in MeOH solvent and air-dried, was packed into a plastic tube and connected to the ozonator setup. Then, dry ozone was flowed (~25 g Nm^{-3}) continuously through the sample for 90 min.

S5.1. NMR spectroscopy

10 mg of each sample were first digested using an aqueous solution (200 μL) of 5 wt% HF and heating at 120 °C overnight. Afterwards, 500 μL of DMSO-d_6 was directly added.

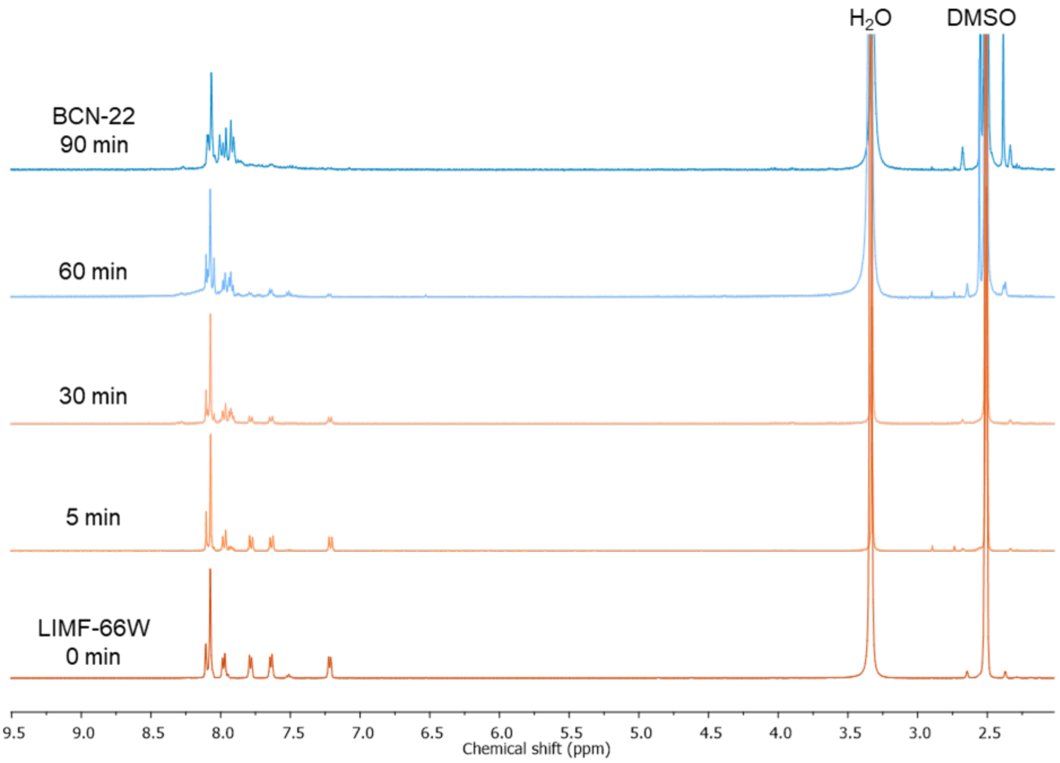

Figure S6. ^{1}H-NMR spectra (400 MHz, DMSO-d_6) displaying transformation from LIMF-66W to BCN-22 according to the ozonolysis time: LIMF-66W, as synthesized (bottom, dark orange); 5 minutes ozonized – 35% converted (orange), 30 minutes ozonized – 50% converted (peach); 60 minutes ozonized – 92% converted (light blue); fully converted BCN-22 sample, ozonized for 90 minutes (blue).

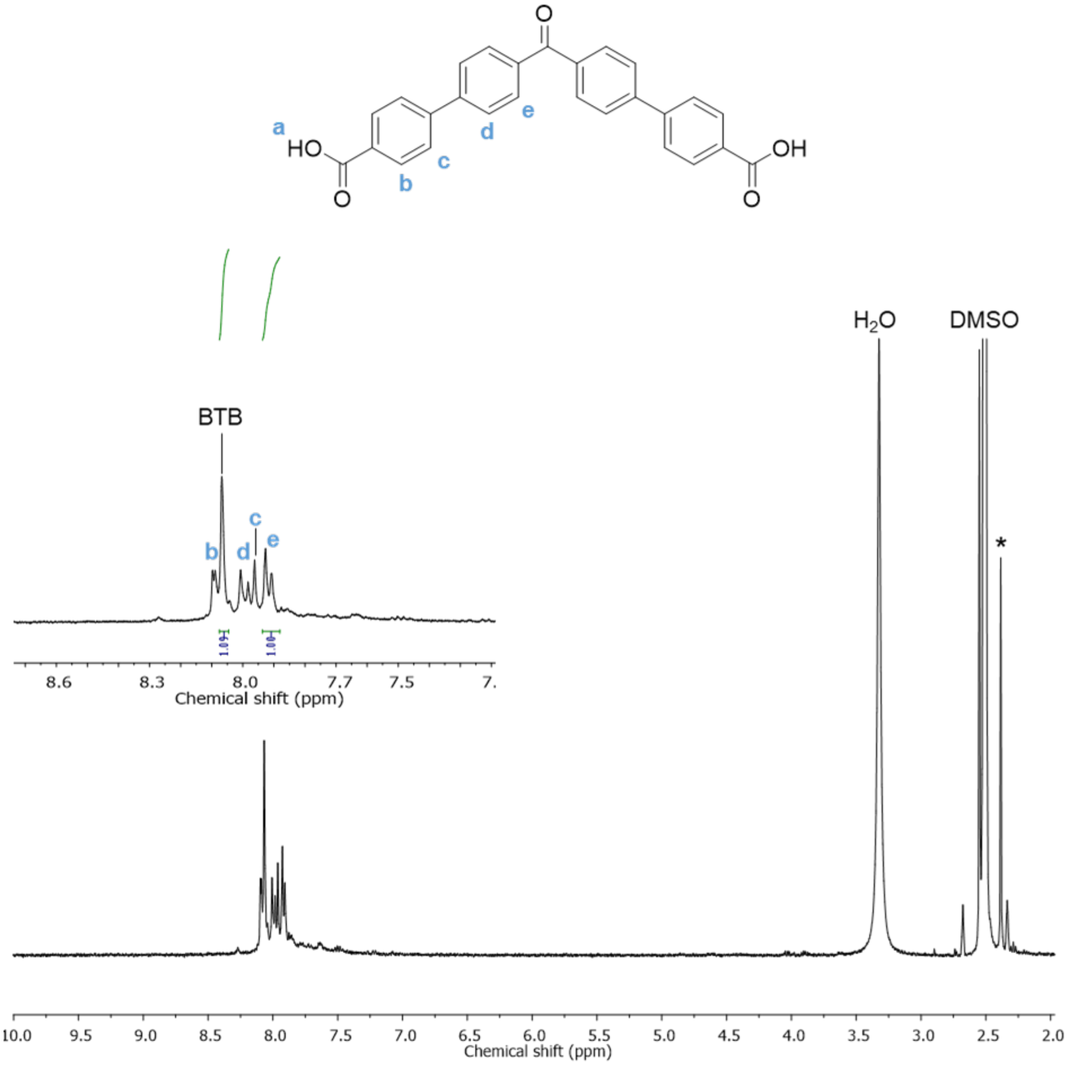

Figure S7. ^{1}H-NMR spectrum (400 MHz) of digested BCN-22 synthesized via solid-gas ozonolysis, displaying the ratio between BTB^{3-} and CBC^{2-}. Note that the experimental ratio of BTB^{3-} over CBC^{2-} is 1:1, matching the expected one.

S5.2. ESI-MS

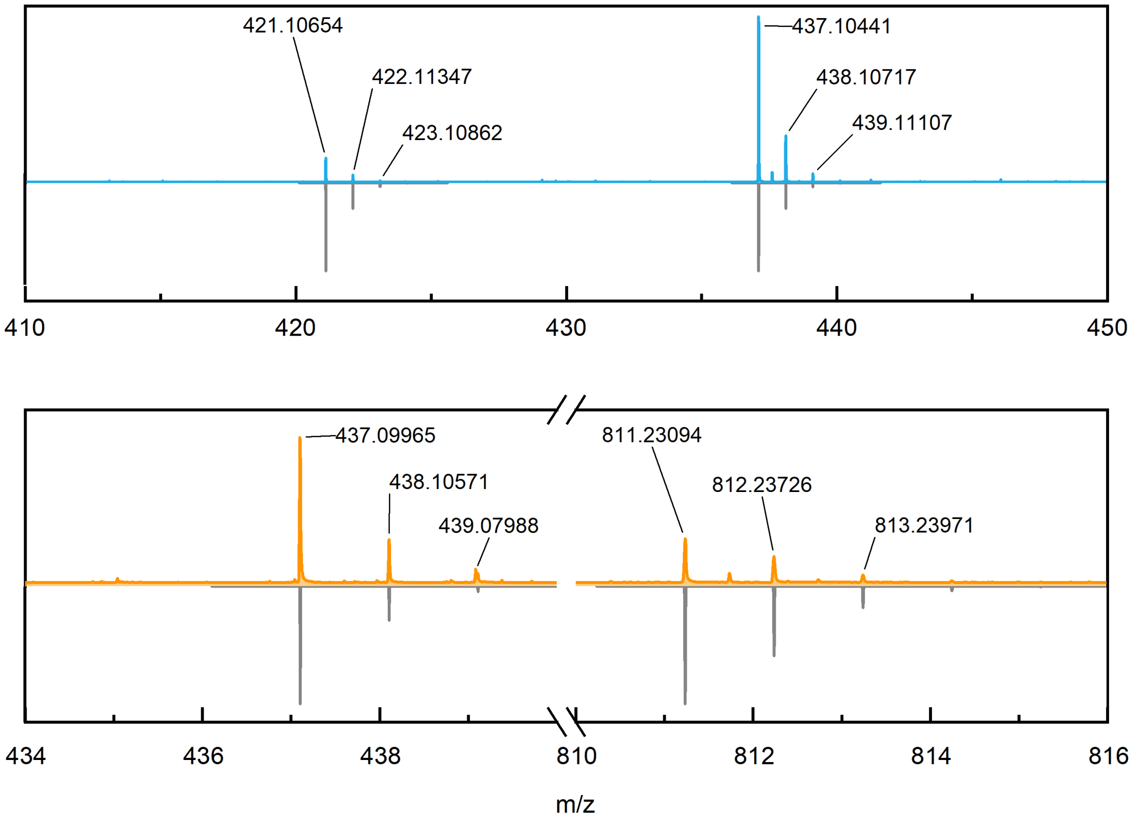

Figure S8. ESI-MS spectrum LIMF-66W (bottom, experimental data in orange, calculated in grey) and BCN-22 (top experimental data in blue, calculated in grey). Note that H_3BTB was found at m/z = 437.09965 and m/z = 437.10441 g/mol in LIMF-66W and BCN-22, respectively, and expected at m/z = 437.1026 g/mol, proving this ligand remained unaltered. H_4ETTC was found at m/z = 811.23094 g/mol and expected at m/z = 811.23492 g/mol only in LIMF-66W. H_2CBC was found at m/z = 421.10654 g/mol and expected at m/z = 421.10859 g/mol only in BCN-22.

S5.3. FT-IR

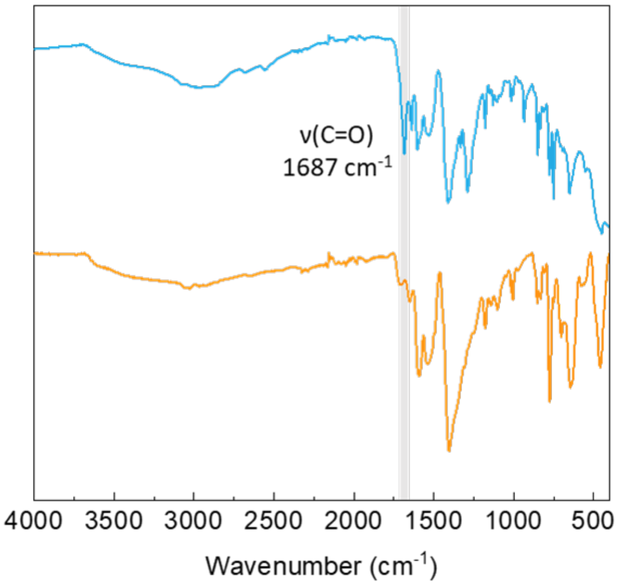

Figure S9. FT-IR spectra of LIMF-66W (orange) and BCN-22 (blue), displaying the appearance of the C=O stretch ascribed to the resulting H_2CBC linker of BCN-22.

S5.4. PXRD

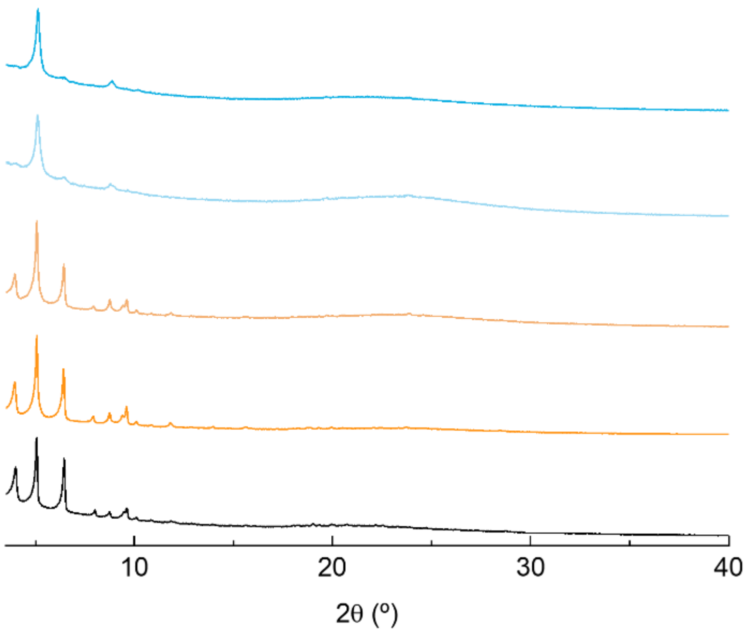

Figure S10. PXRD patterns displaying transformation from LIMF-66W to BCN-22 according to the ozonolysis time, from bottom to top: LIMF-66W, as synthesized (bottom, black); 5 minutes ozonized (orange); 30 minutes ozonized (peach); 60 minutes ozonized (light blue); BCN-22, ozonized for 90 minutes (blue).

S5.5. Scanning Electron Microscopy (SEM)

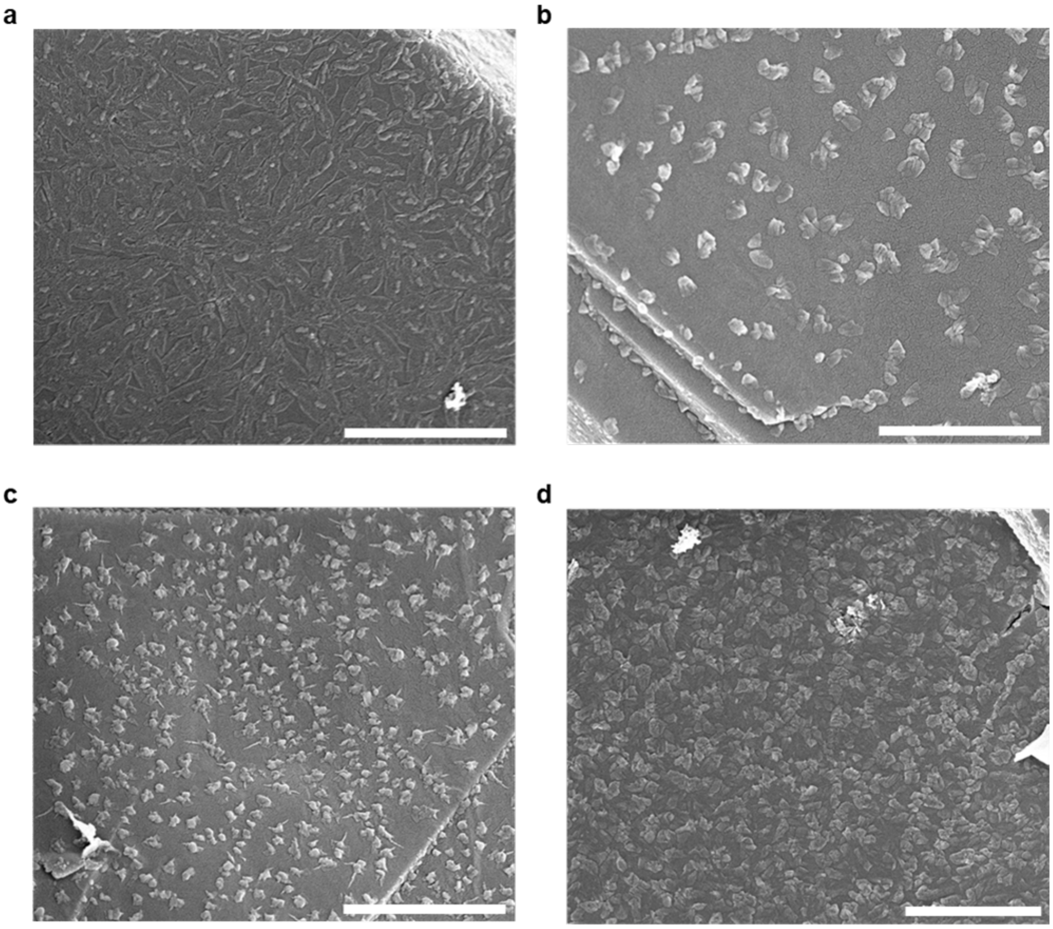

Figure S11. SEM images of the surface of (a) 5 minutes ozonized, (b) 30 minutes ozonized, (c) 60 minutes ozonized, and (d) BCN-22, 90 minutes ozonized. All this samples were prepared using the solid-gas ozonolysis protocol. Scale bars: 2 μm.

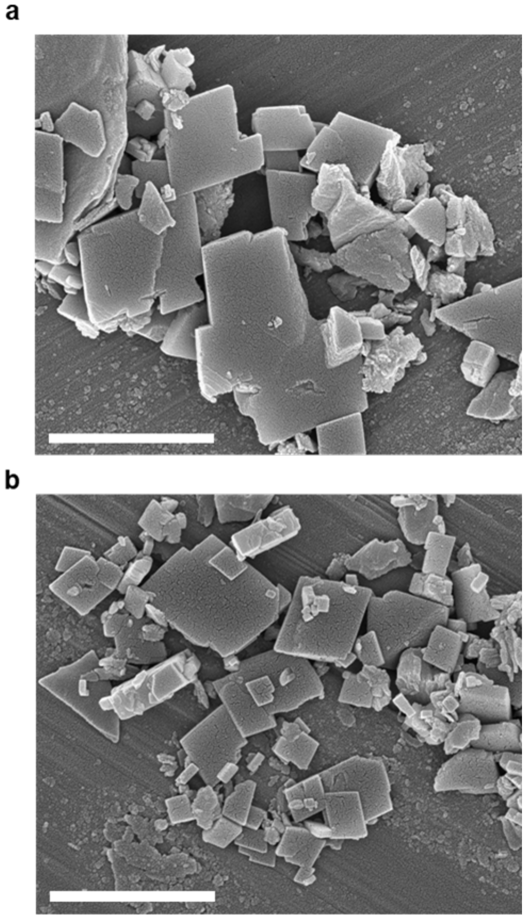

Figure S12. SEM images of BCN-22 ozonized in solid-gas, immersed and sonicated in MeOH.

Scale bars: 2 μm.

Section S6. Synthesis of BCN-22 via ozonolysis in dispersion

BCN-22 was obtained through an ozonolysis reaction in dispersion. Briefly, 10 mg of LIMF-66W were suspended in 2 mL MeOH in a 10 mL round-bottom flask sealed with a septum. Two needles were used, one as an outlet and one as an inlet connected to the ozonator setup. Then, dry ozone was flowed (~25 g Nm^{-3}) continuously through the sample for 90 minutes. Elemental analysis found (calculated) for $(C_{108}H_{62}O_{34}Zr_6)\cdot(DMF)_2\cdot(H_2O)_{12}$, which represents $C_{128}H_{106}O_{54}Zr_6$: C% 45.60 (48.67), H% 3.65 (3.58), N% 0.93 (1).

This protocol could be performed in 10-fold upscale. To this effect, 100 mg of LIMF-66W were suspended in 25 mL MeOH in a 100 mL three-neck round-bottom flask. The stream of ozone was passed through a 10 mL plastic pipette tip through the middle neck of the flask, and 1 mL plastic pipette tips through the two necks on the sides. Then, dry ozone was flowed (~30 g Nm^{-3}) continuously through the sample for 90 minutes.

S6.1. Isolation of nanosheets of BCN-22

Sonication of BCN-22 in a suspension in 2 mL of MeOH was performed for 10 minutes at 90% power. Then, the sample was centrifuged at 13000 rpm for 5 minutes allowing for the purification of nanosheets of BCN-22, which remained in the supernatant and could therefore be isolated from bigger particles within the sample. Subsequently, nanosheets were obtained in average 30% yield. These experiments were repeated four times to estimate the average yield, calculated by evaporating the solvent, weighting the remaining solid and comparing to the weight of the original samples.

S6.2. Tyndall effect

a

b

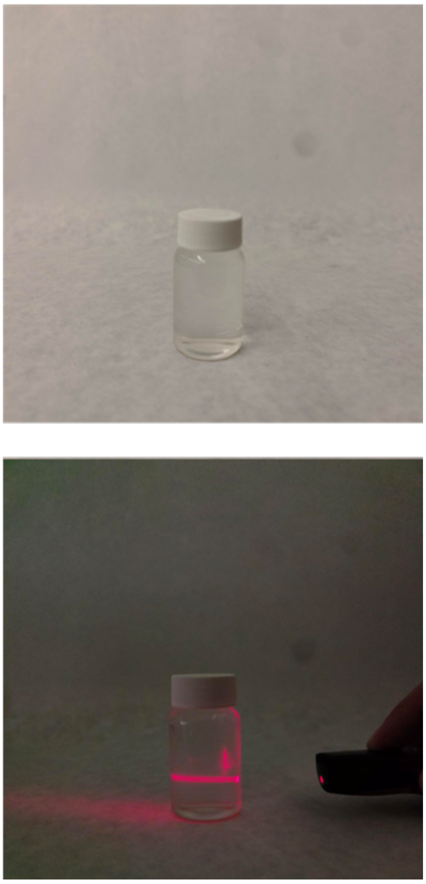

Figure S13. Image of the Tyndall effect observed upon irradiation with a laser beam of the colloidal sample of BCN-22 MONs obtained through the upscaled ozonolysis in MeOH dispersion, prior to its use for AFM measurements.

S6.3. Formation of BCN-22 nanosheets

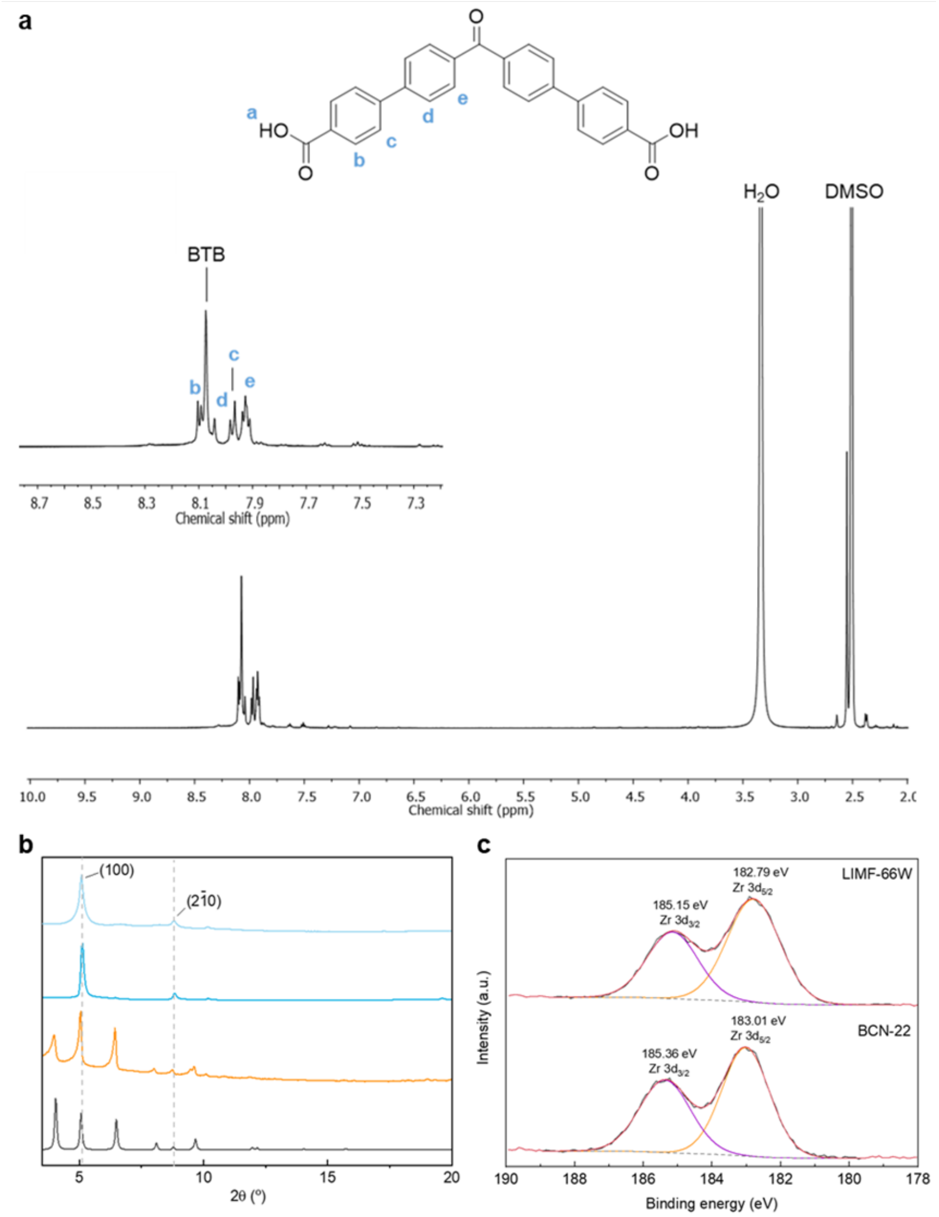

Figure S14. (a) ^{1}H-NMR spectrum (400 MHz) of digested BCN-22 nanosheets synthesized via ozonolysis in MeOH, displaying the ratio between BTB^{3-} and CBC^{2-}. Note that the experimental ratio of BTB^{3-}over CBC^{2-} is 1:1, matching the expected one. (b) PXRD pattern of the calculated LIMF-66W (black, bottom), as synthesized LIMF-66W (orange, middle), BCN-22 bulk (blue, middle) and BCN-22 nanosheets (light blue, top). (c) Zr 3d spectra from XPS measurements of as-synthesized LIMF-66W (top) and BCN-22 nanosheets (bottom), showing the characteristic peaks of Zr $3d_{5/2}$ and $3d_{3/2}$, with no significant differences.[4-6]

S6.4. Scanning Electron Microscopy (SEM)

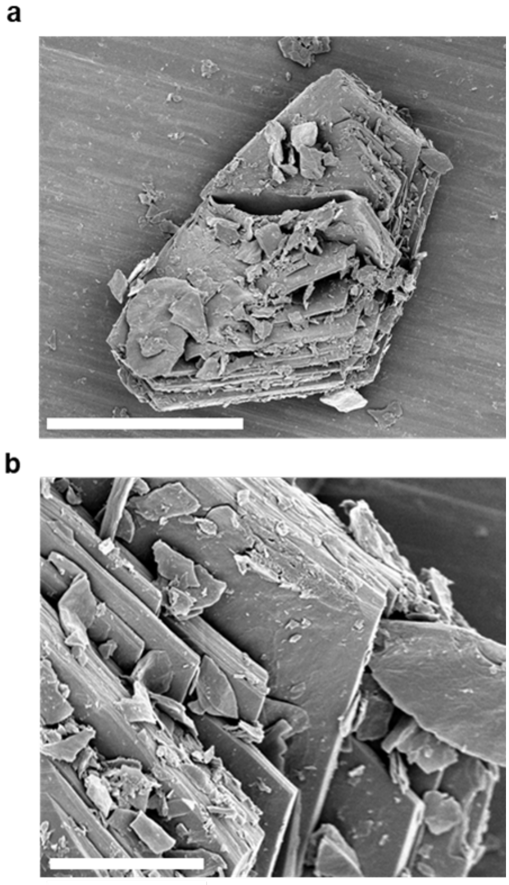

Figure S15. SEM images of the blank test performed by sonicating a sample of LIMF-66W without ozonolysis treatment. Scale bars: 50 μm (a) and 2 μm (b).

S6.6. Atomic Force Microscopy

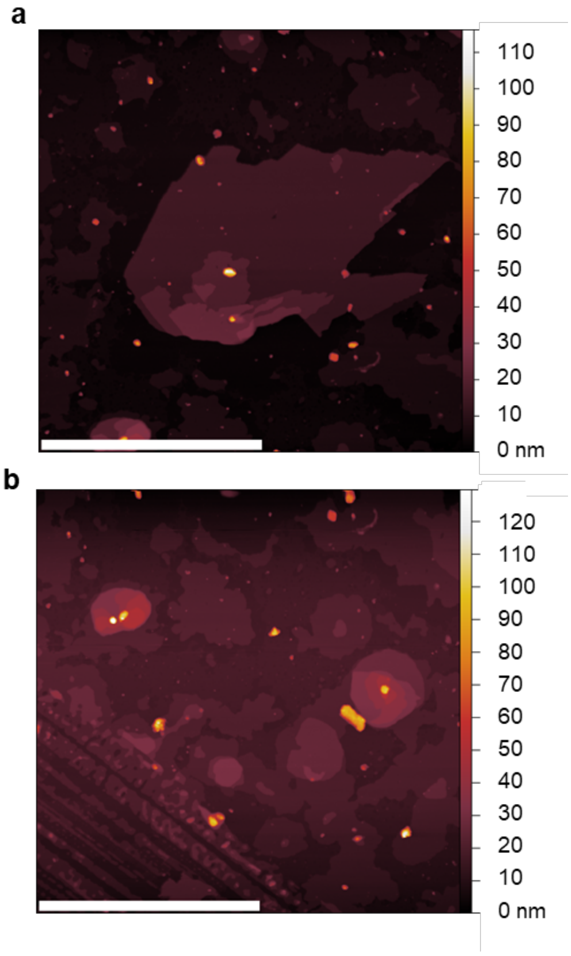

Figure S16. Representative AFM images of BCN-22 nanosheets. Scale bars: 1 μm.

S6.6. Transmission Electron Microscopy (TEM)

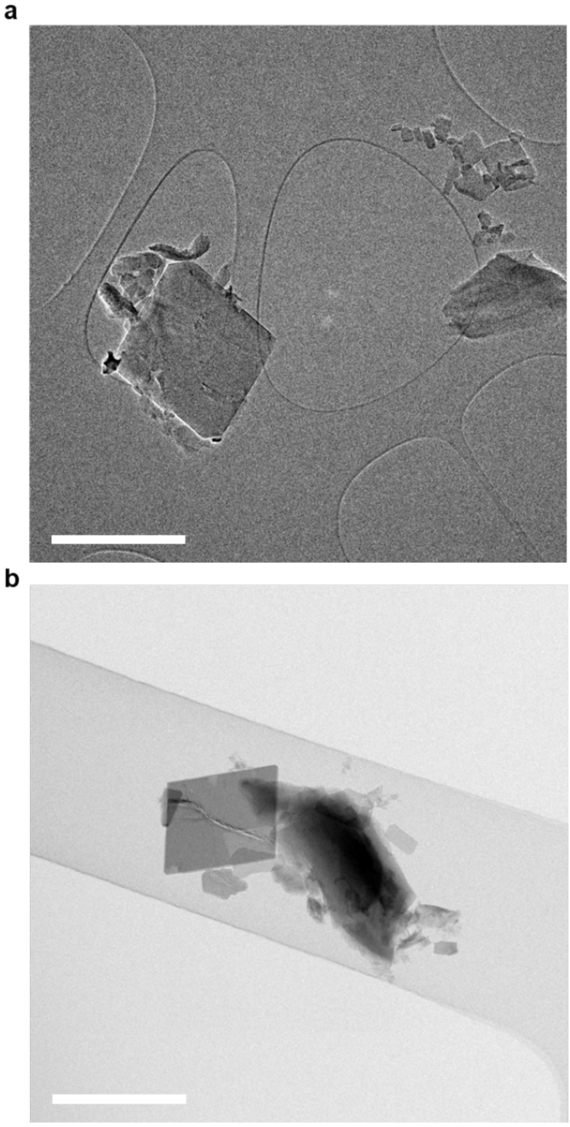

Figure S17. TEM images of BCN-22 nanosheets. Scale bars: 1 μm.

S6.7. BET and Pore Size Distribution measurements

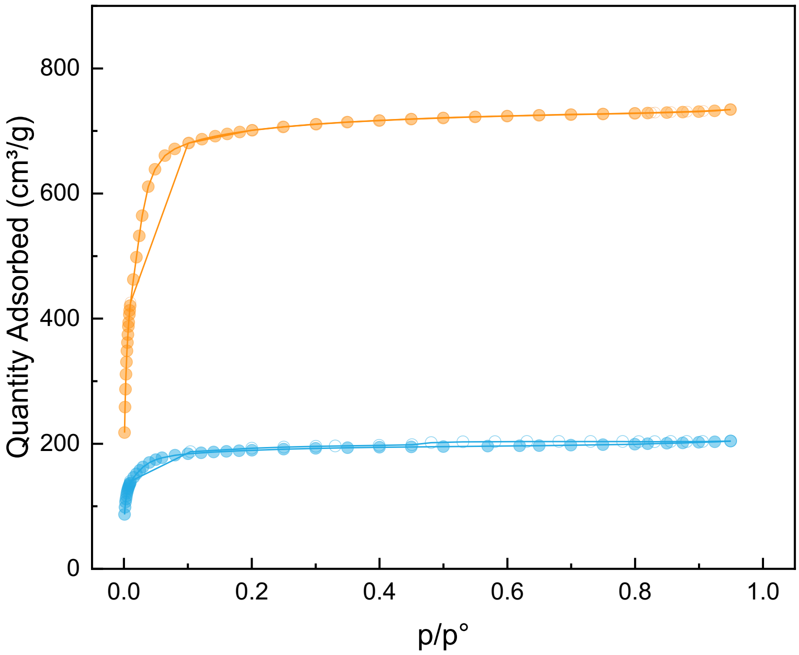

Figure S18. N_2 sorption isotherm (77 K) for LIMF-66W (orange) and BCN-22 (blue).

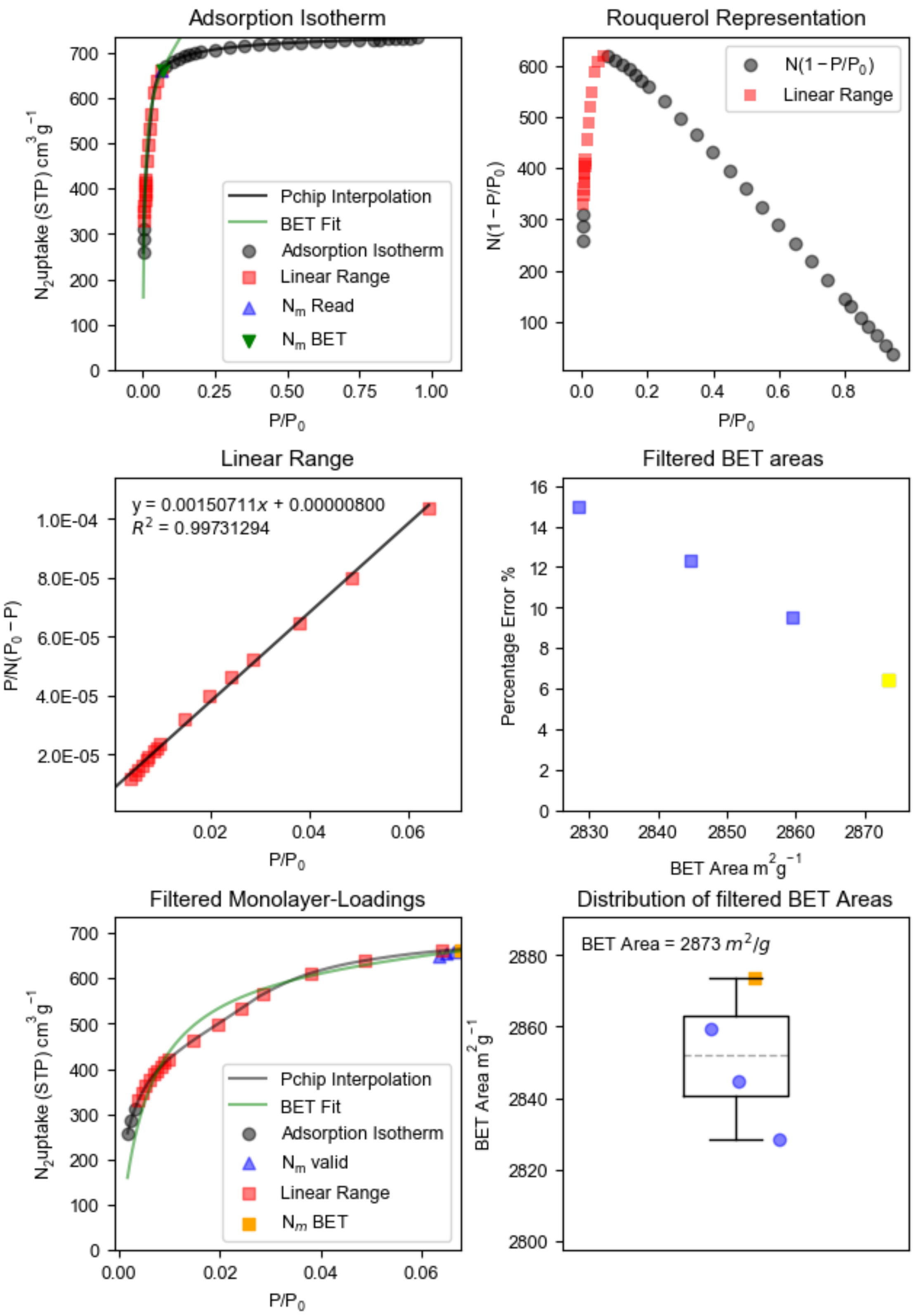

Figure S19. BETSI analysis of LIMF-66W.

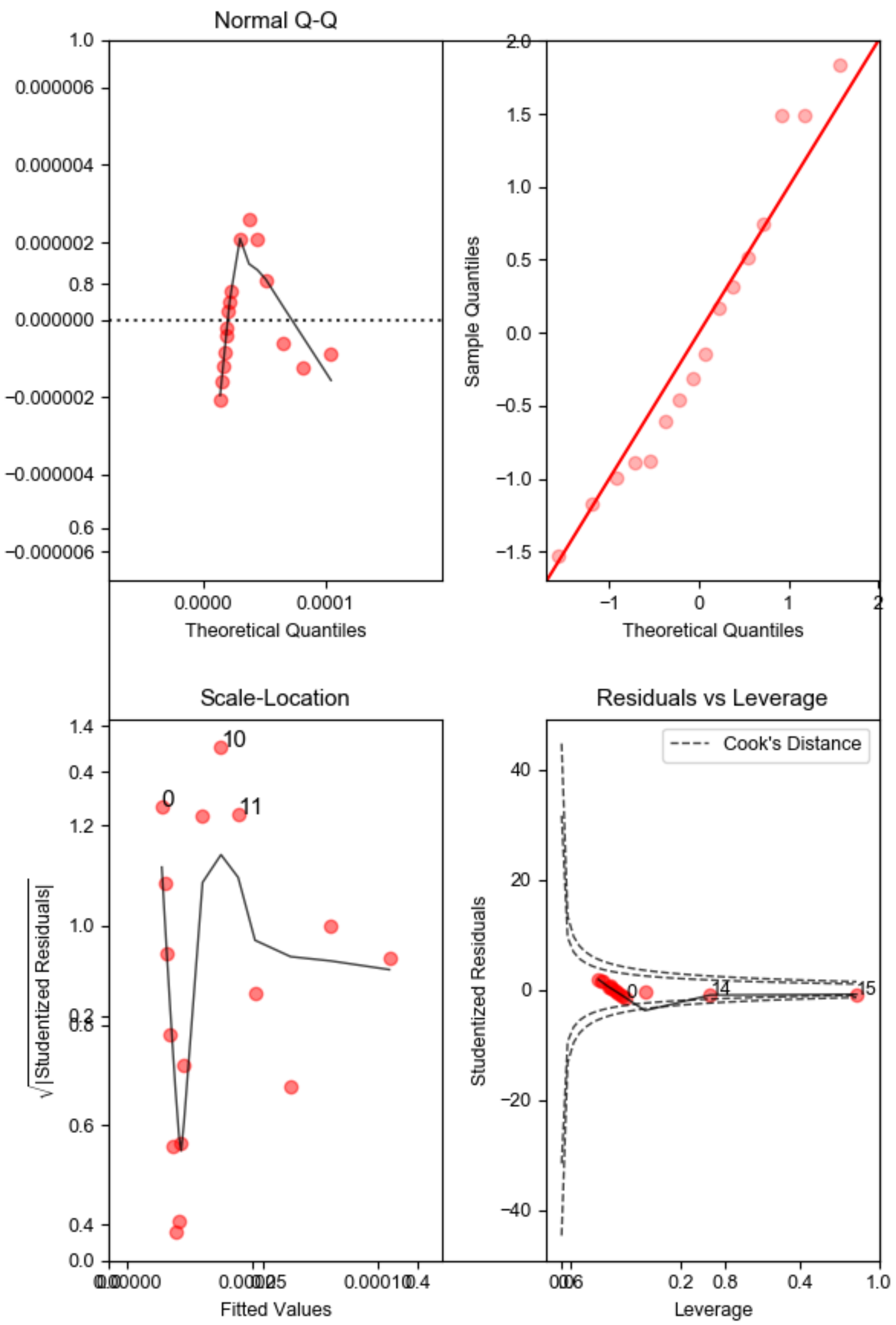

Figure S20. BETSI regression diagnostics for LIMF-66W.

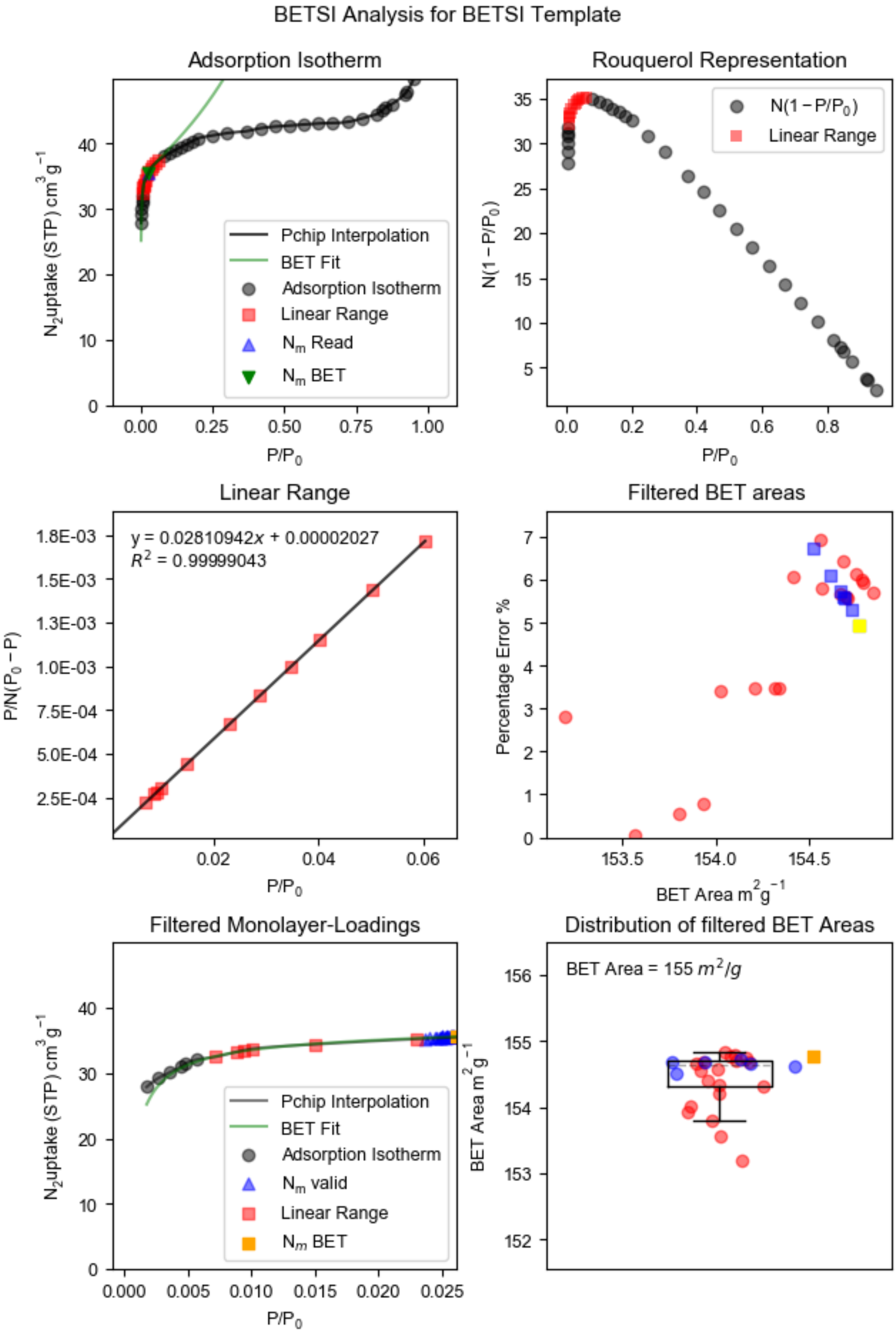

Figure S21. BETSI analysis of BCN-22.

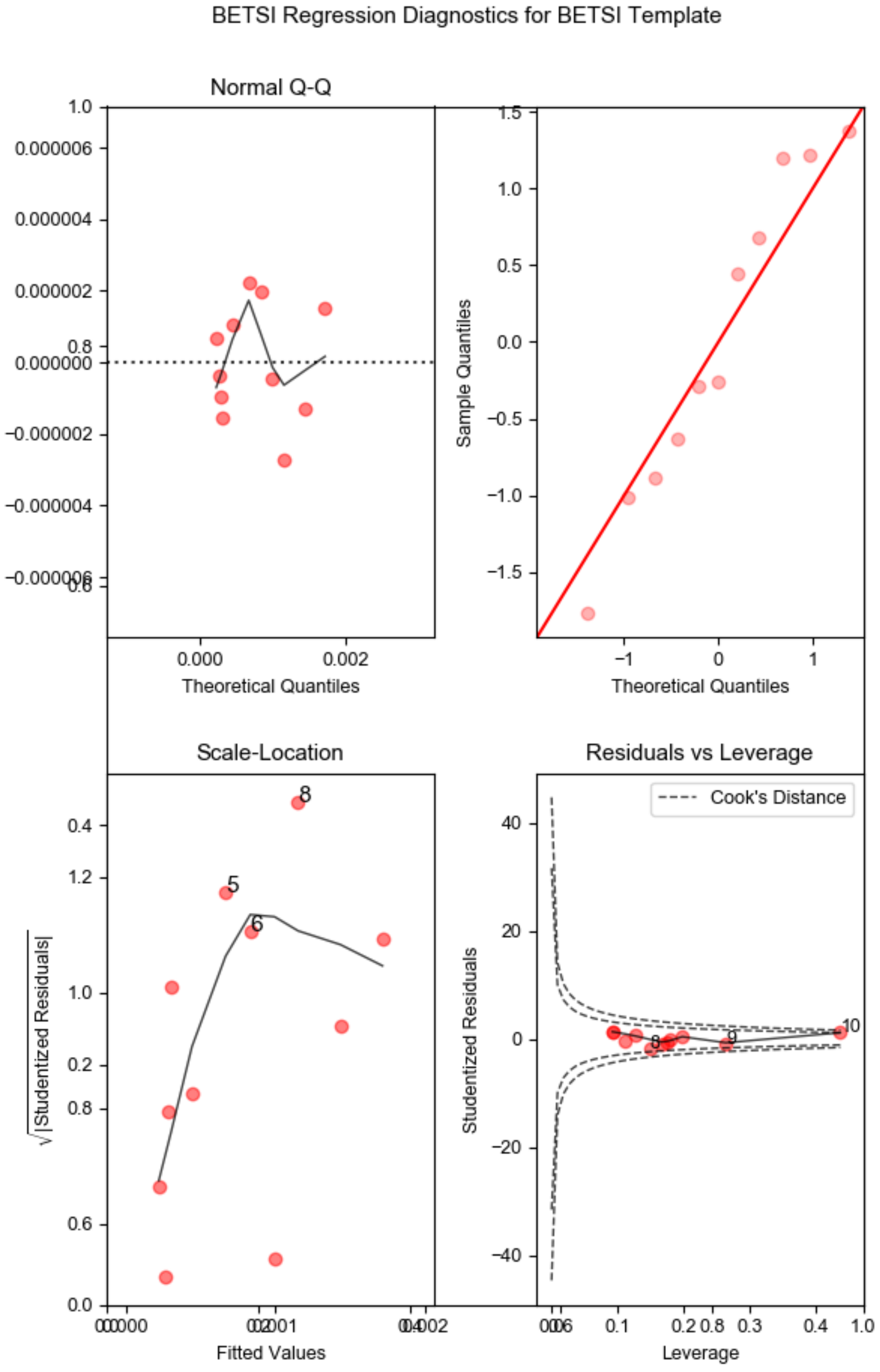

Figure S22. BETSI regression diagnostics for BCN-22.

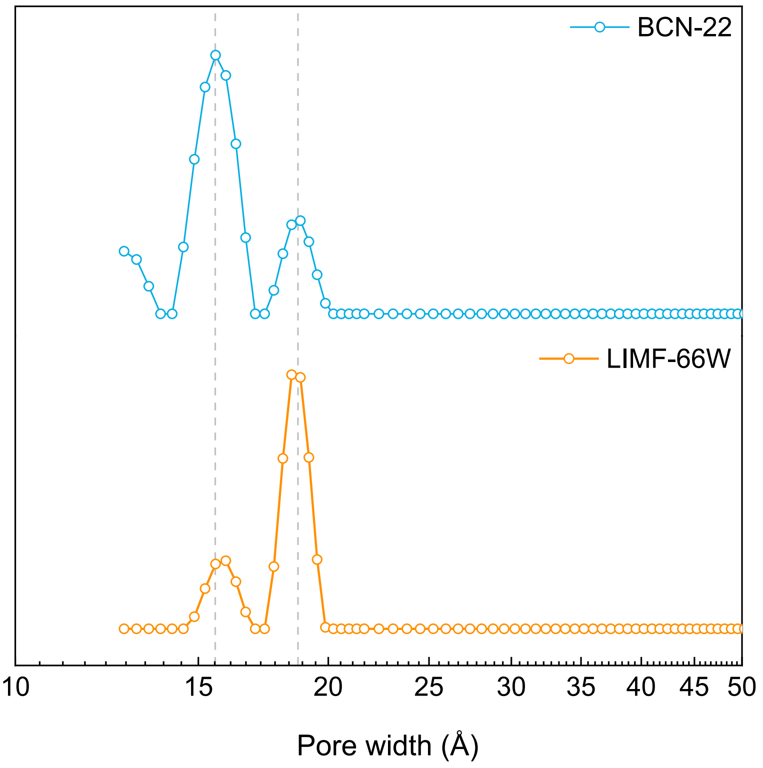

Figure S23. Pore size distribution analysis of LIMF-66W (orange) and BCN-22 (blue) using DFT model for N_2 77K.

Section S7. Catalytic experiment

S7.1. General protocol

1 mmol of oleic acid (282.5 mg) and 8 mmol of the respective alcohol were added in a 6 mL screw cap vial, followed by the addition of the catalyst and 3 Å molecular sieves. The resulting mixture was allowed to stir at 100 °C for the duration of 6 hours or 12 hours. After the respective duration, the reaction mixture was centrifuged to separate the catalysts. Then, the solution was analysed by ^{1}H-NMR spectroscopy in $CDCl_3$ or DMSO-d_6 solvent. In the ^{1}H-NMR spectroscopy, the α-proton of the ester formed during esterification showed a distinct peak. To calculate the % of yield, the integral of this α-proton peak was compared with the alkene protons of oleic acid or oleate (at 5.3 ppm for DMSO-d_6 and at 5.45 ppm for $CDCl_3$). For the blank experiments, the same process was followed without any catalysts.

Recyclability experiments were done following the same protocol for the reaction from oleic acid to ethyl oleate. After the catalytic process, used catalysts were washed thoroughly with ethanol and then were used for the next cycle.

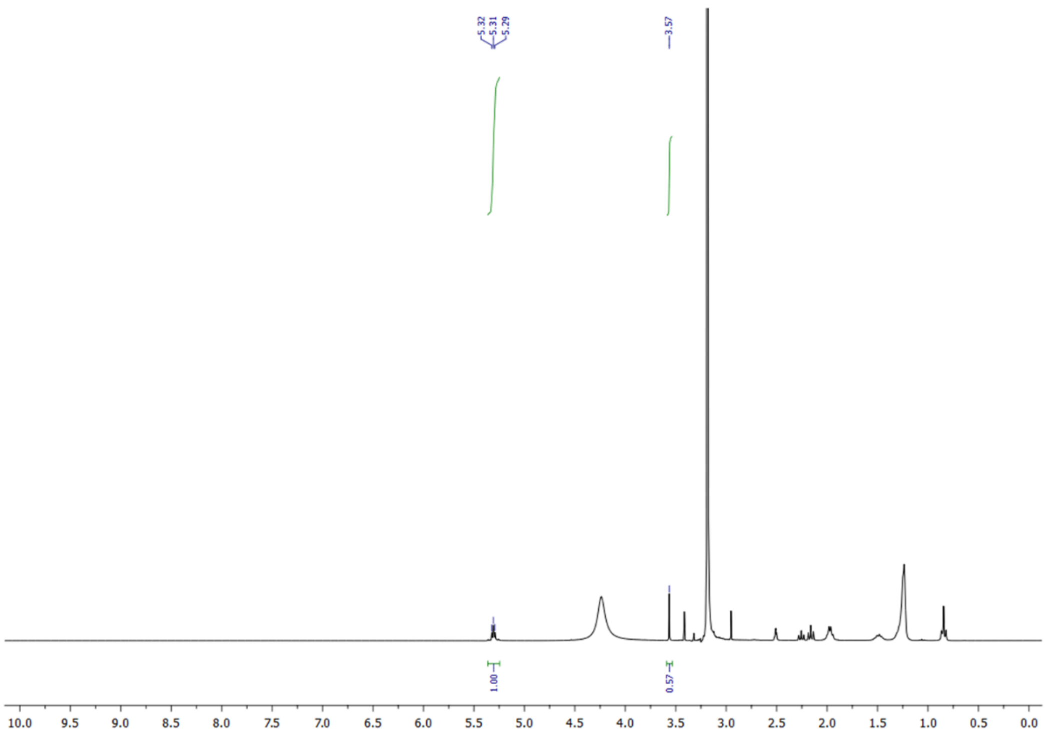

Figure S24. ^{1}H-NMR spectra (300 MHz, DMSO-d_6) of the reaction mixture between oleic acid and methanol catalysed by BCN-22 at 100 °C for a duration of 6 hours.

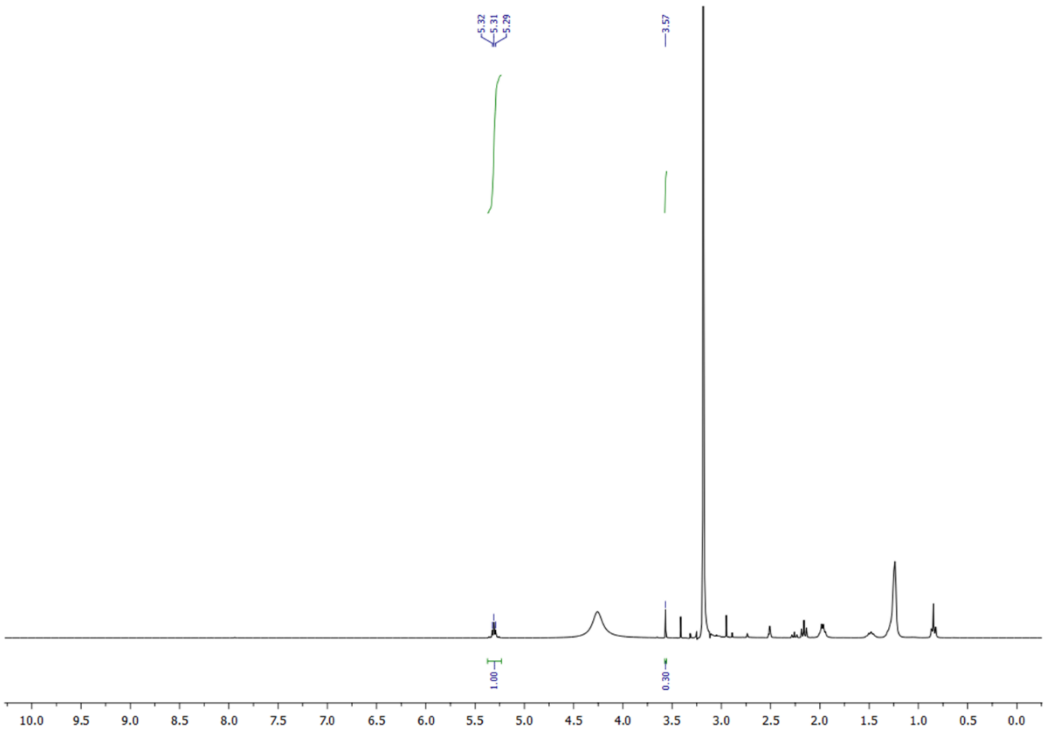

Figure S25. ^{1}H-NMR spectra (300 MHz, DMSO-d_6) of the reaction mixture between oleic acid and methanol catalysed by LIMF-66W at 100 °C for a duration of 6 hours.

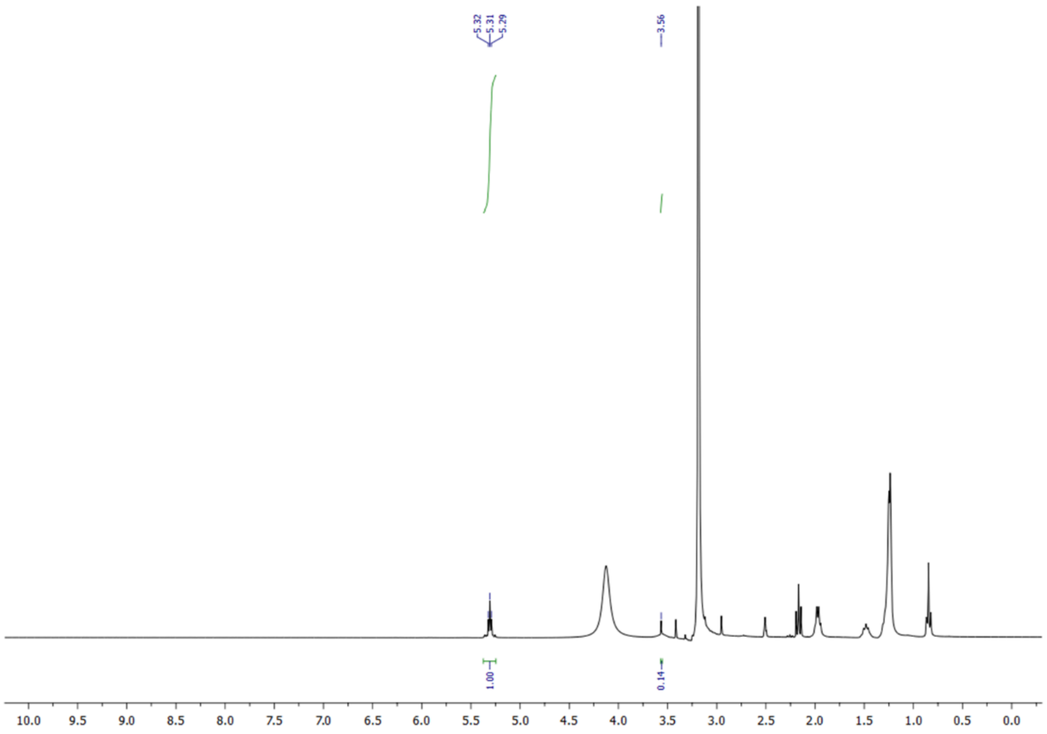

Figure S26. ^{1}H-NMR spectra (300 MHz, DMSO-d_6) of the reaction mixture between oleic acid and methanol without catalyst at 100 °C for a duration of 6 hours.

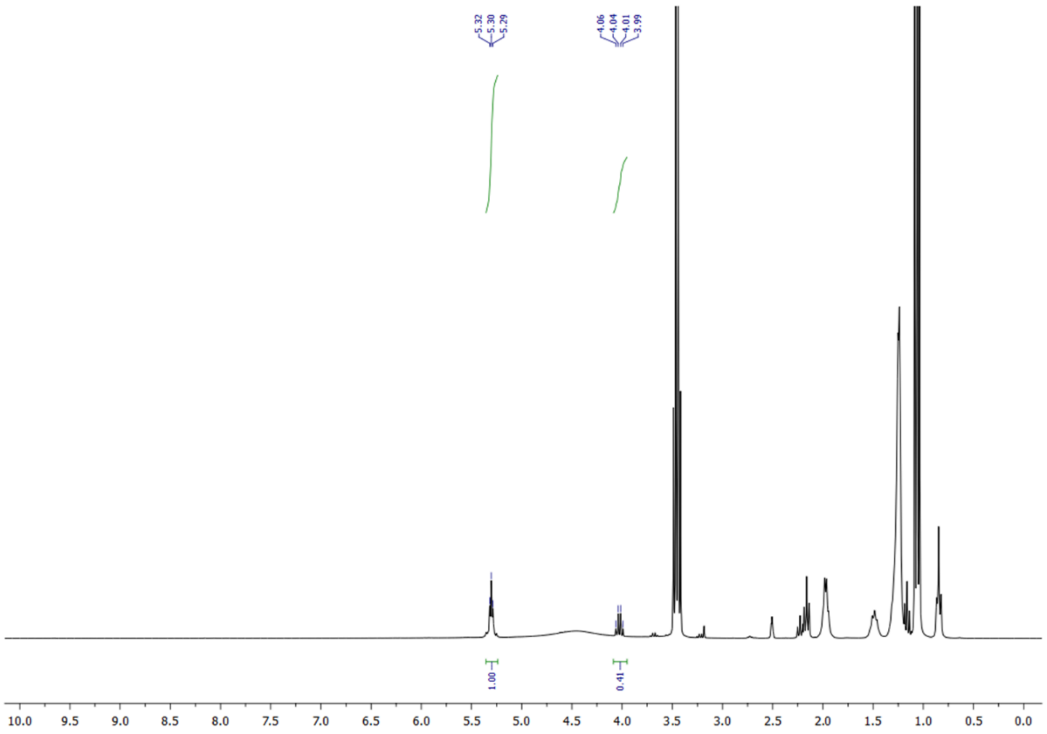

Figure S27. ^{1}H-NMR spectra (300 MHz, DMSO-d_6) of the reaction mixture between oleic acid and ethanol catalysed by BCN-22 at 100 °C for a duration of 6 hours.

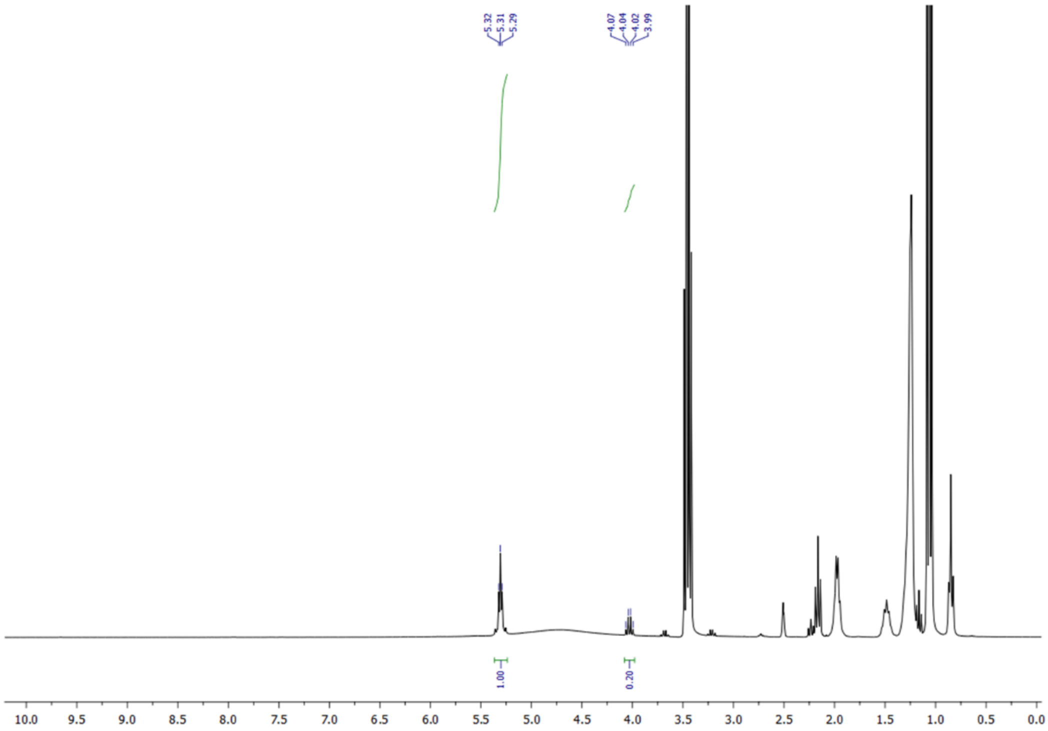

Figure S28. ^{1}H-NMR spectra (300 MHz, DMSO-d_6) of the reaction mixture between oleic acid and ethanol catalysed by LIMF-66W at 100 °C for a duration of 6 hours.

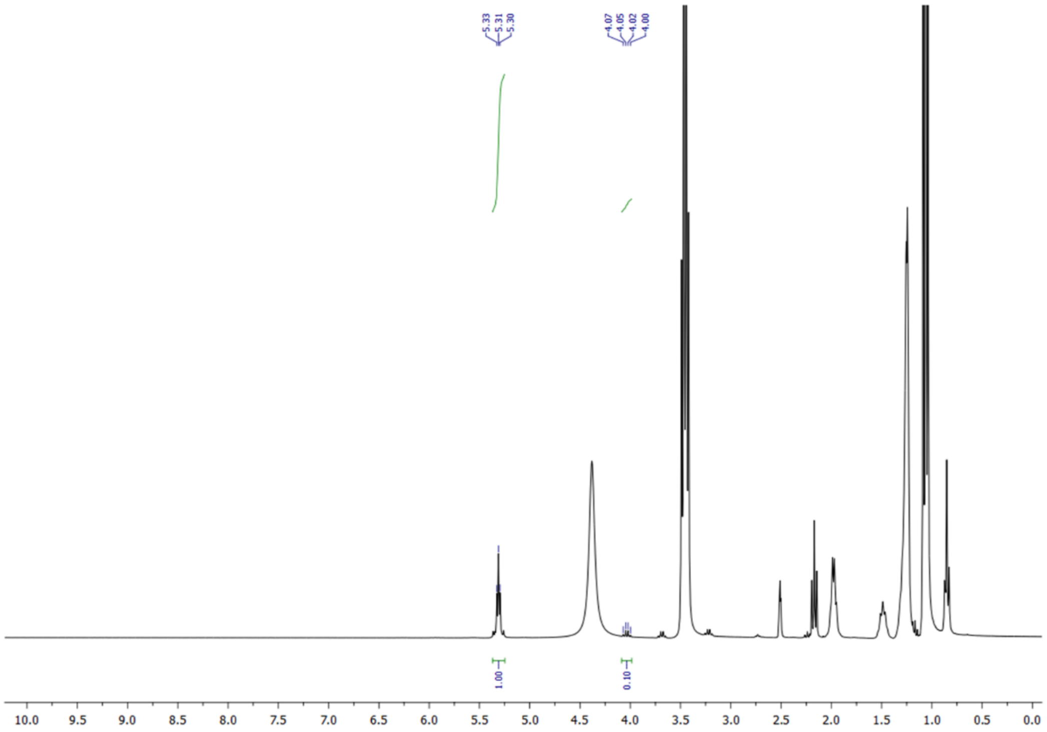

Figure S29. ^{1}H-NMR spectra (300 MHz, DMSO-d_6) of the reaction mixture between oleic acid and ethanol without catalyst at 100 °C for a duration of 6 hours.

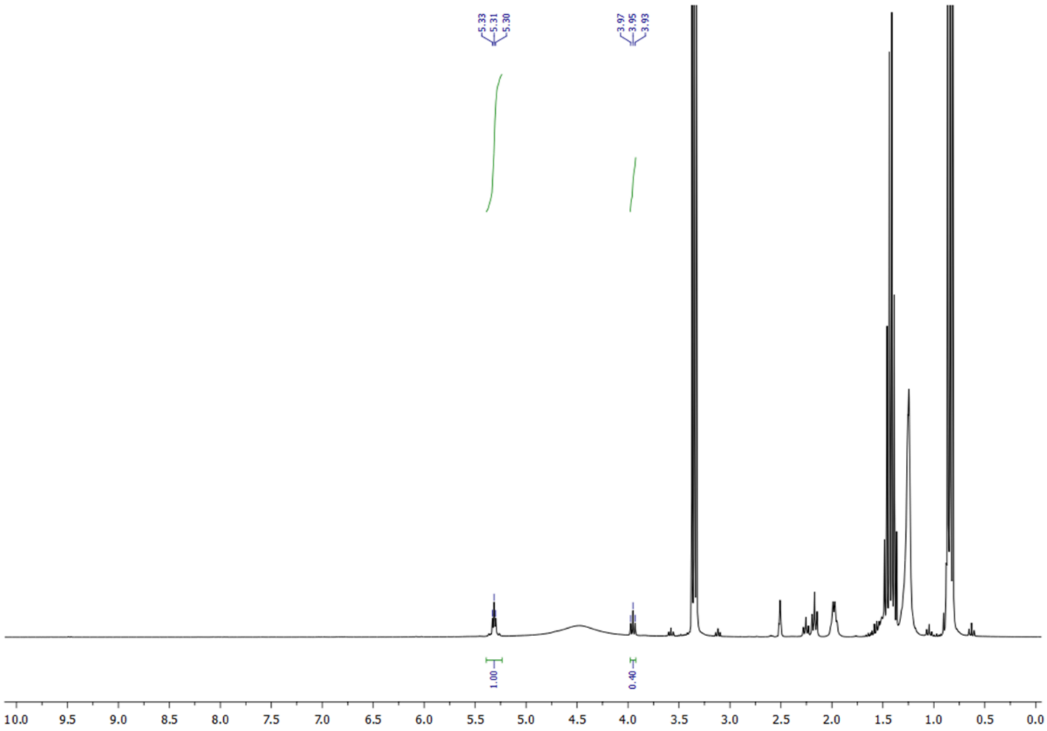

Figure S30. ^{1}H-NMR spectra (300 MHz, DMSO-d_6) of the reaction mixture between oleic acid and 1-propanol catalysed by BCN-22 at 100 °C for a duration of 6 hours.

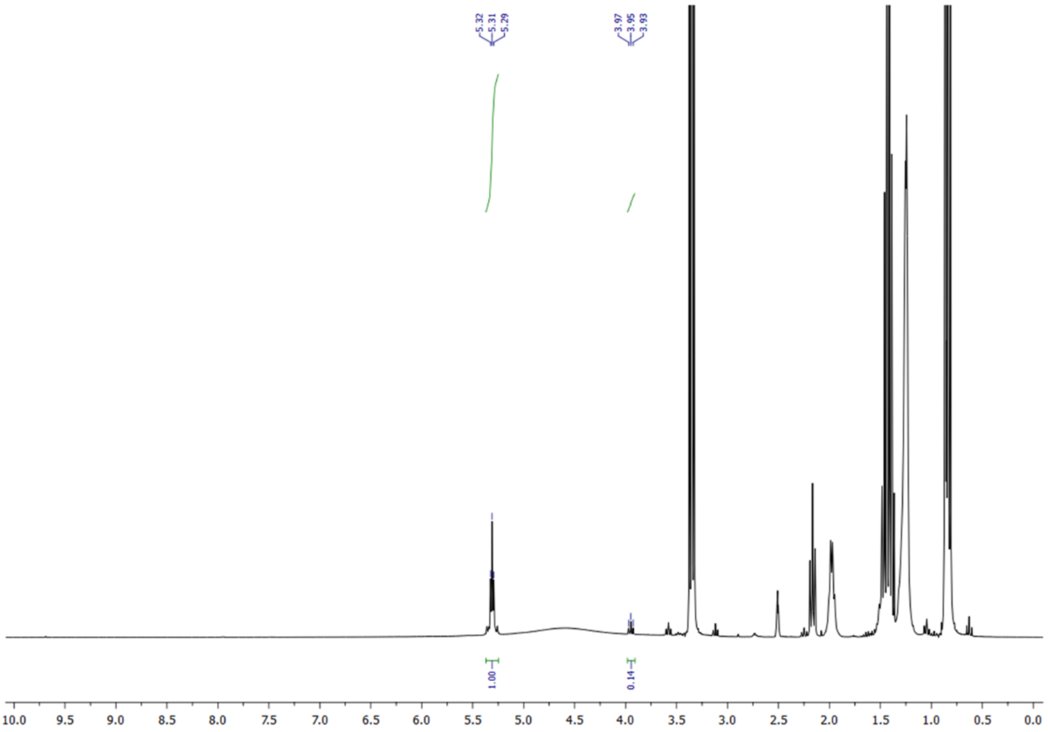

Figure S31. ^{1}H-NMR spectra (300 MHz, DMSO-d_6) of the reaction mixture between oleic acid and 1-propanol catalysed by LIMF-66W at 100 °C for a duration of 6 hours.

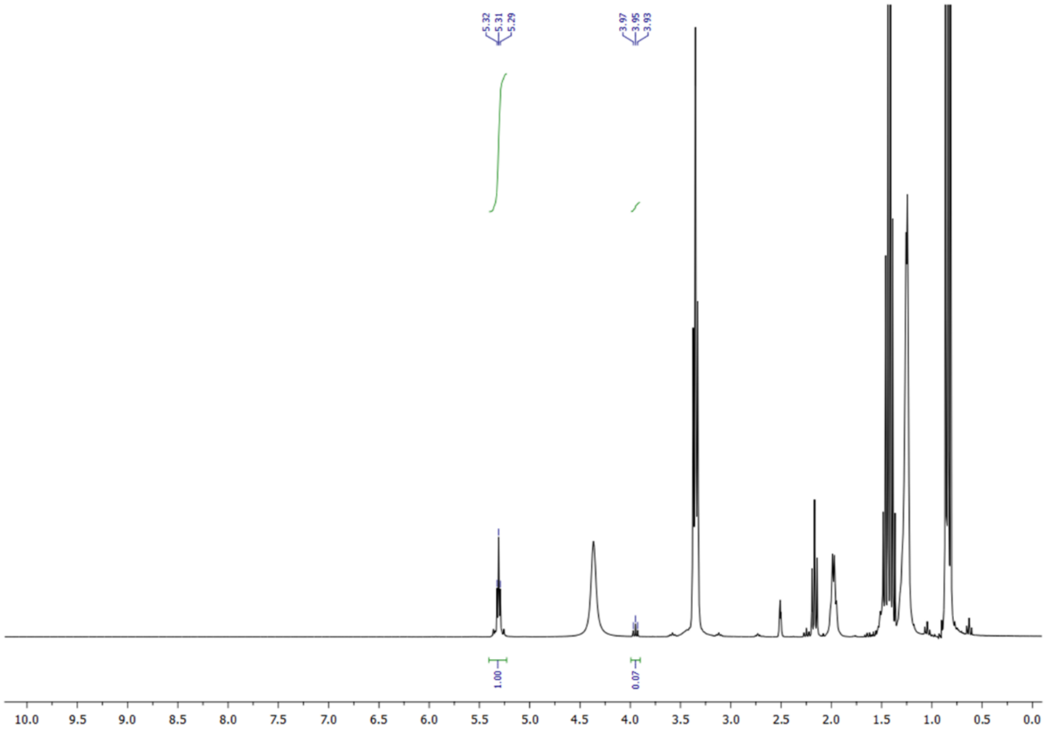

Figure S32. ^{1}H-NMR spectra (300 MHz, DMSO-d_6) of the reaction mixture between oleic acid and 1-propanol without catalyst at 100 °C for a duration of 6 hours.

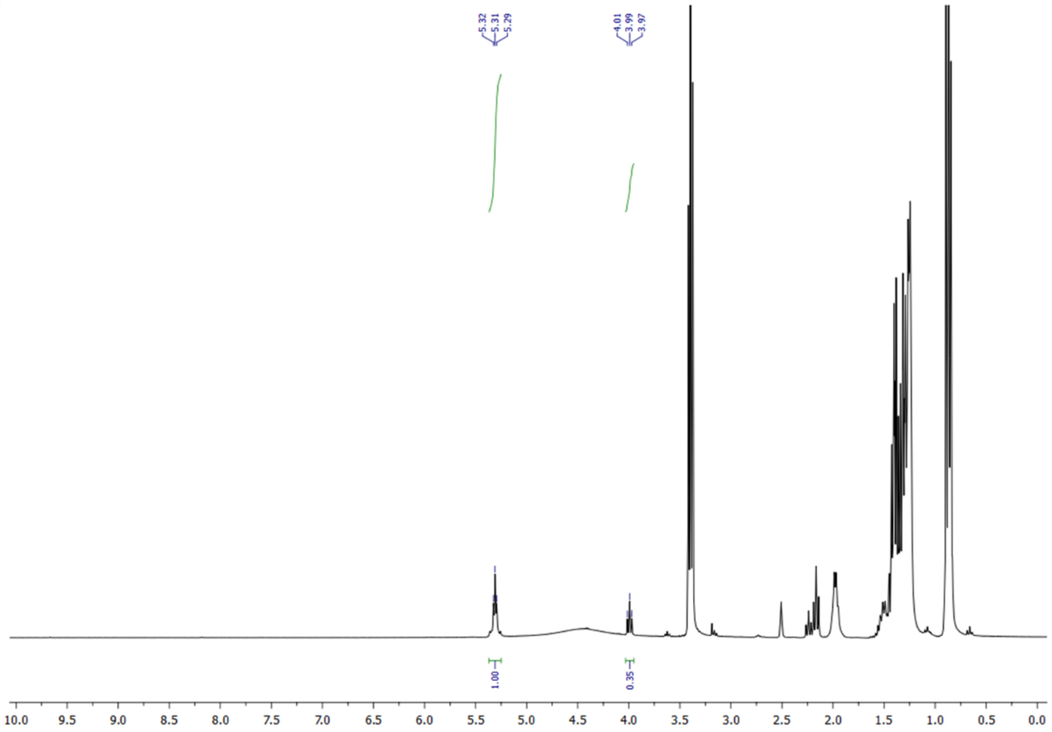

Figure S33. ^{1}H-NMR spectra (300 MHz, DMSO-d_6) of the reaction mixture between oleic acid and 1-butanol catalysed by BCN-22 at 100 °C for a duration of 6 hours.

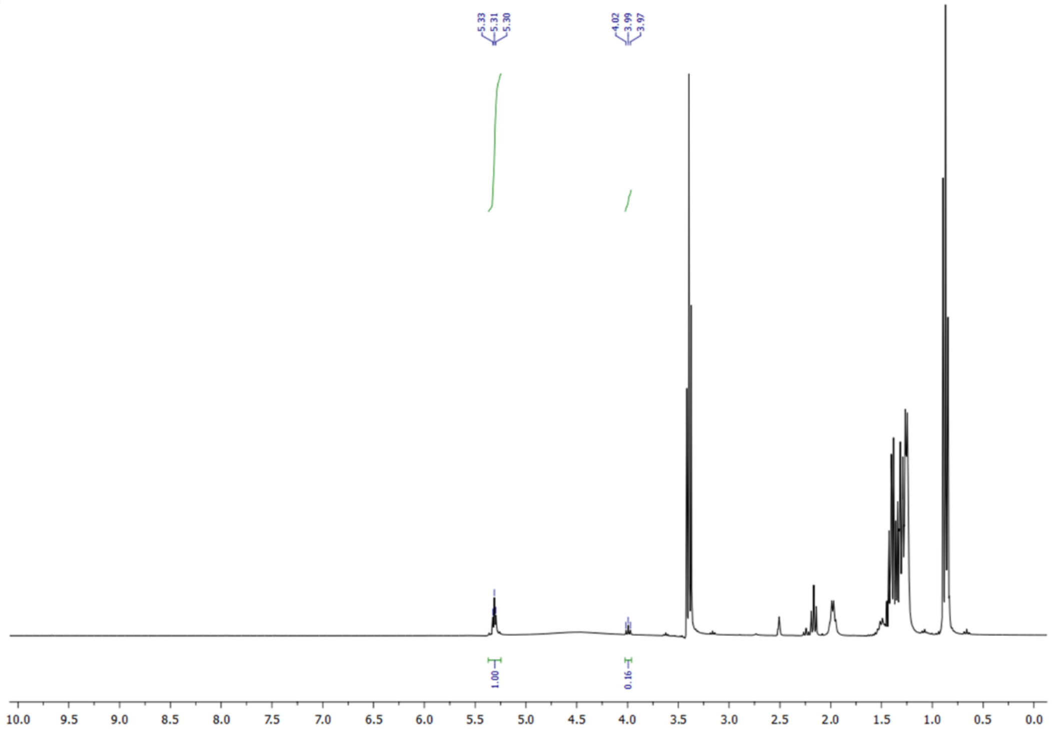

Figure S34. ¹H-NMR spectra (300 MHz, DMSO-*d*₆) of the reaction mixture between oleic acid and 1-butanol catalysed by LIMF-66W at 100 °C for a duration of 6 hours.

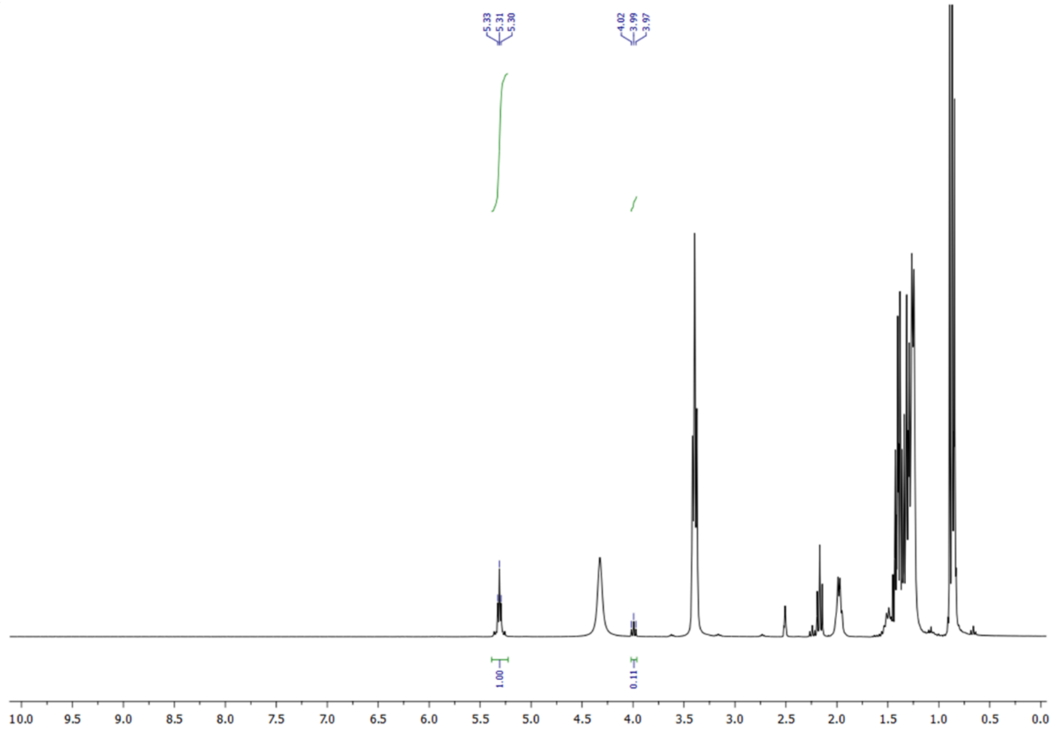

Figure S35. ¹H-NMR spectra (300 MHz, DMSO-*d*₆) of the reaction mixture between oleic acid and 1-butanol without catalyst at 100 °C for a duration of 6 hours.

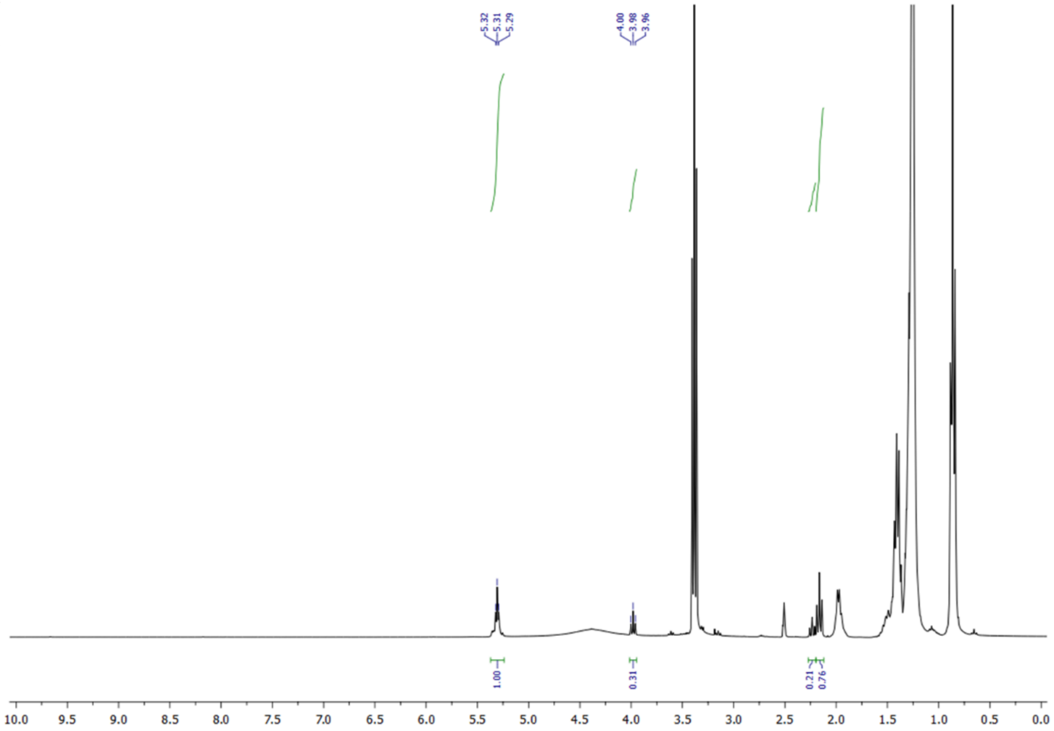

Figure S36. ^{1}H-NMR spectra (300 MHz, DMSO-d_6) of the reaction mixture between oleic acid and 1-hexanol catalysed by BCN-22 at 100 °C for a duration of 6 hours.

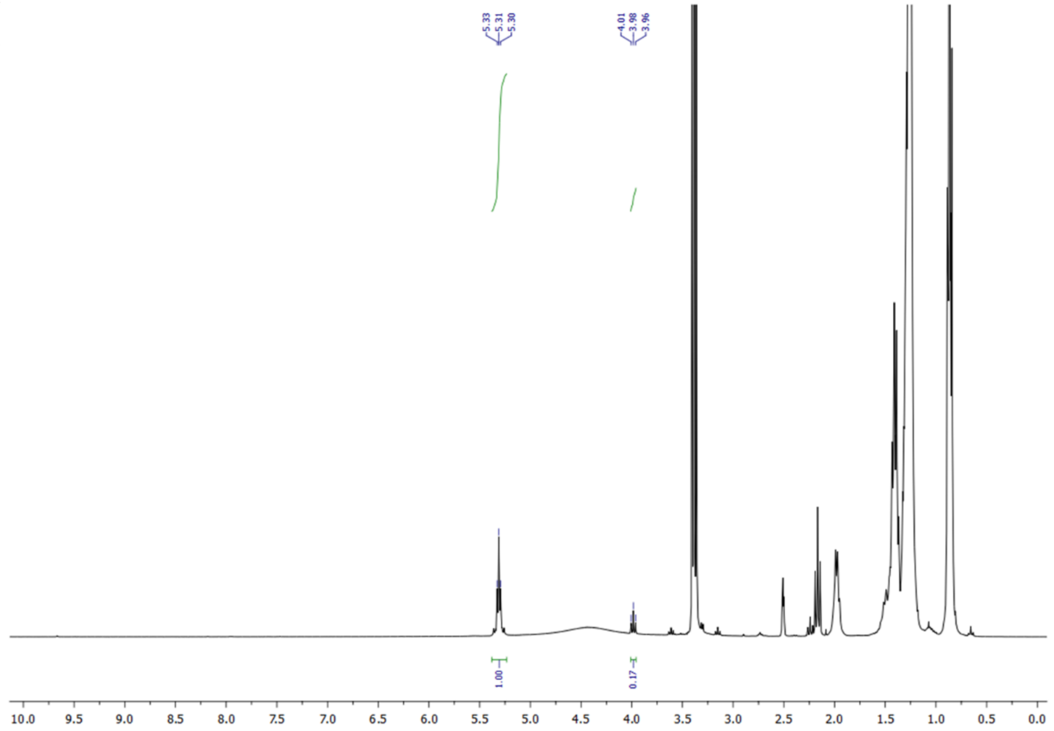

Figure S37. ^{1}H-NMR spectra (300 MHz, DMSO-d_6) of the reaction mixture between oleic acid and 1-hexanol catalysed by LIMF-66W at 100 °C for a duration of 6 hours.

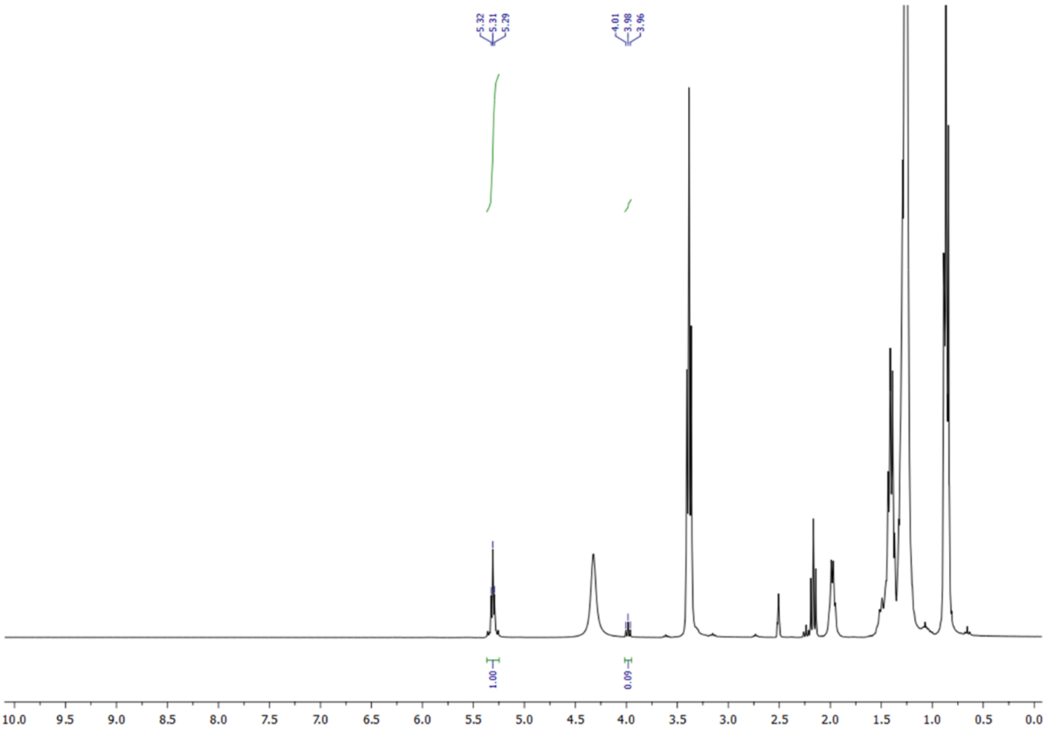

Figure S38. ^{1}H-NMR spectra (300 MHz, DMSO-d_6) of the reaction mixture between oleic acid and 1-hexanol without catalyst at 100 °C for a duration of 6 hours.

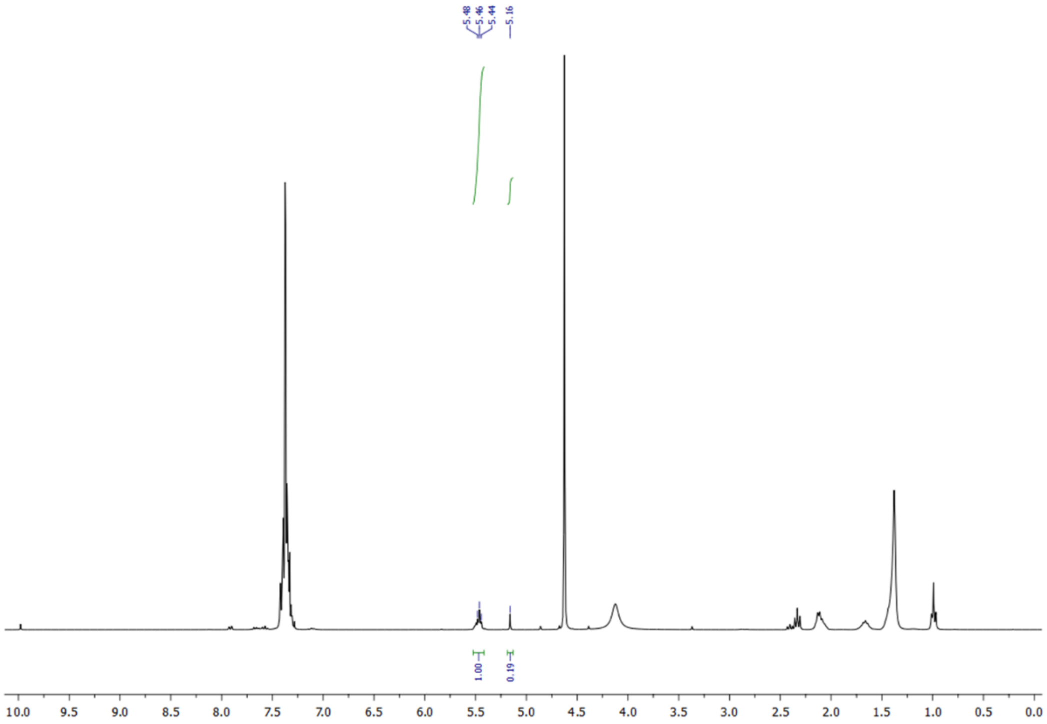

Figure S39. ^{1}H-NMR spectra (300 MHz, $CDCl_3$) of the reaction mixture between oleic acid and benzyl alcohol catalysed by BCN-22 at 100 °C for a duration of 6 hours.

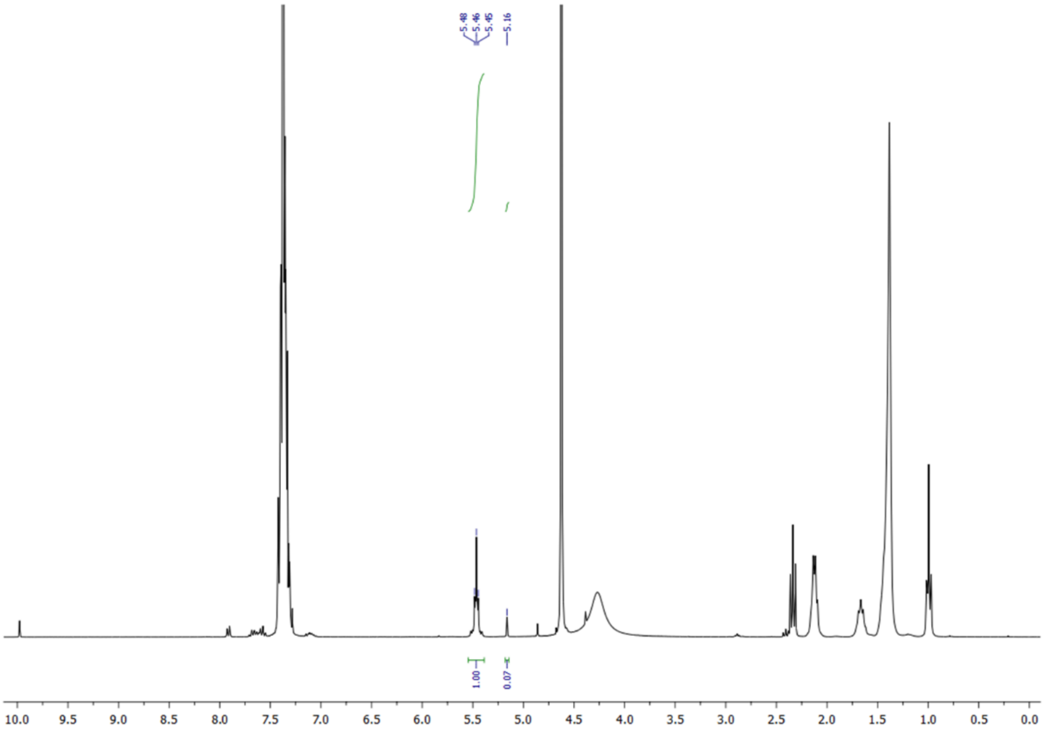

Figure S40. ^{1}H-NMR spectra (300 MHz, $CDCl_3$) of the reaction mixture between oleic acid and benzyl alcohol catalysed by LIMF-66W at 100 °C for a duration of 6 hours.

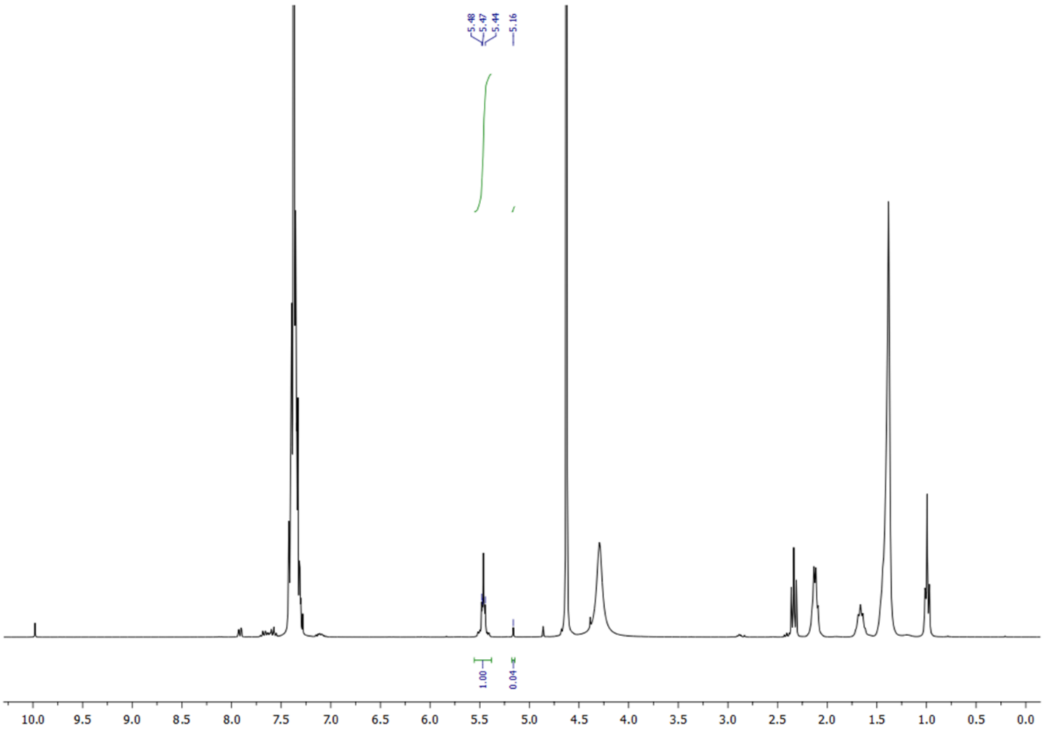

Figure S41. ^{1}H-NMR spectra (300 MHz, $CDCl_3$) of the reaction mixture between oleic acid and benzyl alcohol without catalyst at 100 °C for a duration of 6 hours.

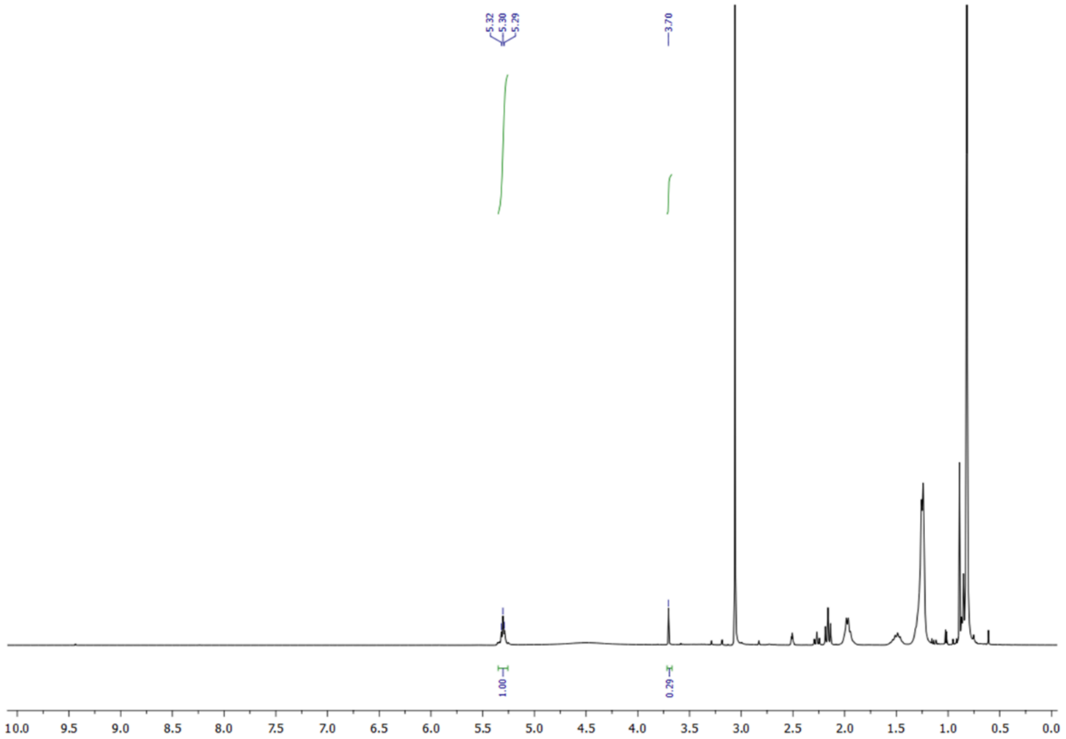

Figure S42. ^{1}H-NMR spectra (300 MHz, DMSO-*d6*) of the reaction mixture between oleic acid and 2,2-dimethyl-1-propanol catalysed by BCN-22 at 100 °C for a duration of 6 hours.

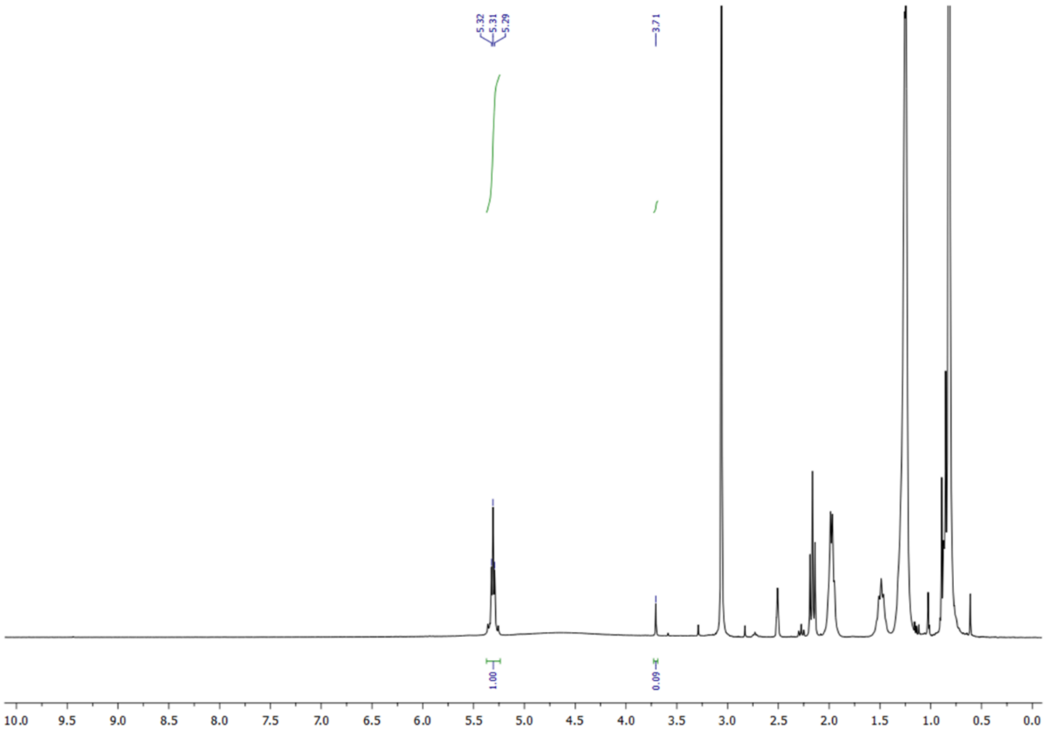

Figure S43. ^{1}H-NMR spectra (300 MHz, DMSO-*d6*) of the reaction mixture between oleic acid and 2,2-dimethyl-1-propanol catalysed by LIMF-66W at 100 °C for a duration of 6 hours.

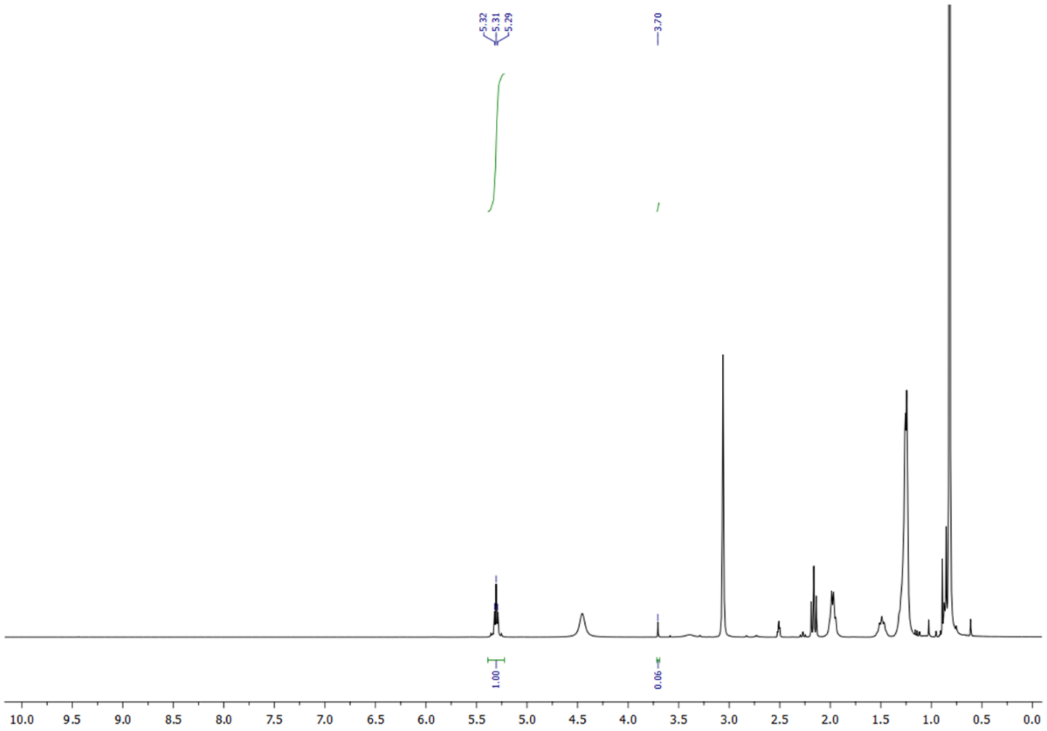

Figure S44. ^{1}H-NMR spectra (300 MHz, DMSO-d_6) of the reaction mixture between oleic acid and 2,2-dimethyl-1-propanol without catalyst at 100 °C for a duration of 6 hours.

Table S1. Catalytic esterification of oleic acid with different alcohols (reaction duration: 6 hours)

Alcohols	BCN-22		LIMF-66W		Blank
	% yield	TON	% yield	TON	% yield
Methanol	57	29	30	15	14
Ethanol	41	21	20	10	10
1-propanol	40	20	14	7	7
1-butanol	35	18	16	8	11
1-hexanol	31	16	17	8	9
Benzyl alcohol	19	10	7	4	4
2,2-dimethyl-1-propanol	29	15	9	5	6

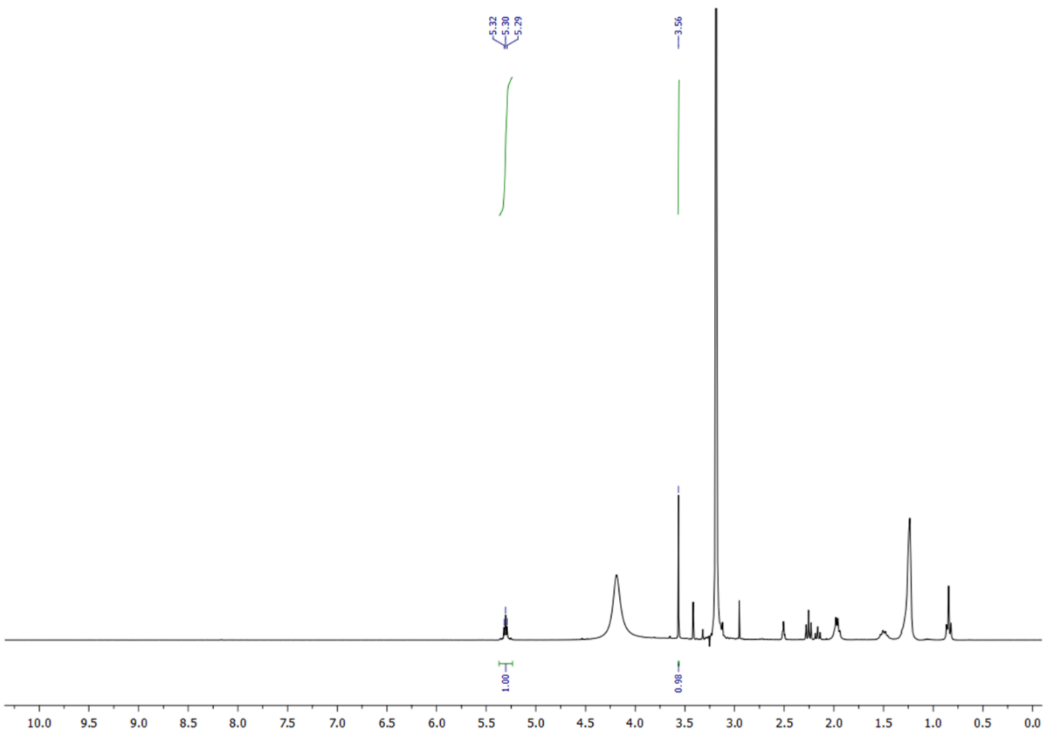

Figure S45. ^{1}H-NMR spectra (300 MHz, DMSO-*d*6) of the reaction mixture between oleic acid and methanol catalysed by BCN-22 at 100 °C for a duration of 12 hours.

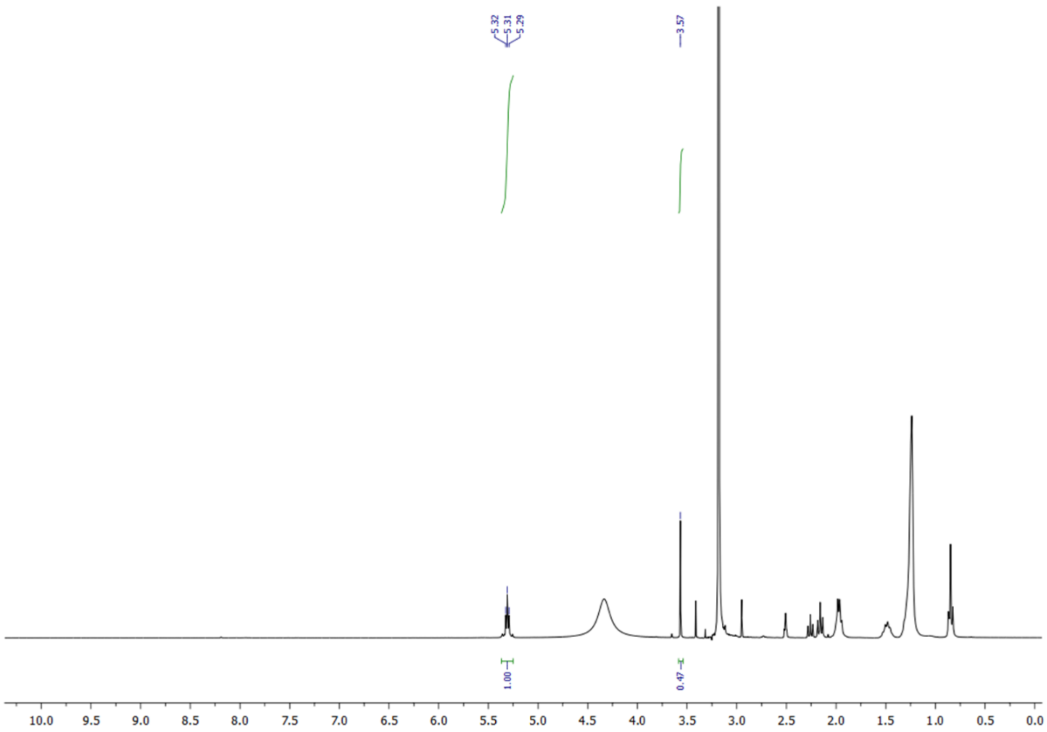

Figure S46. ^{1}H-NMR spectra (300 MHz, DMSO-*d*6) of the reaction mixture between oleic acid and methanol catalysed by LIMF-66W at 100 °C for a duration of 12 hours.

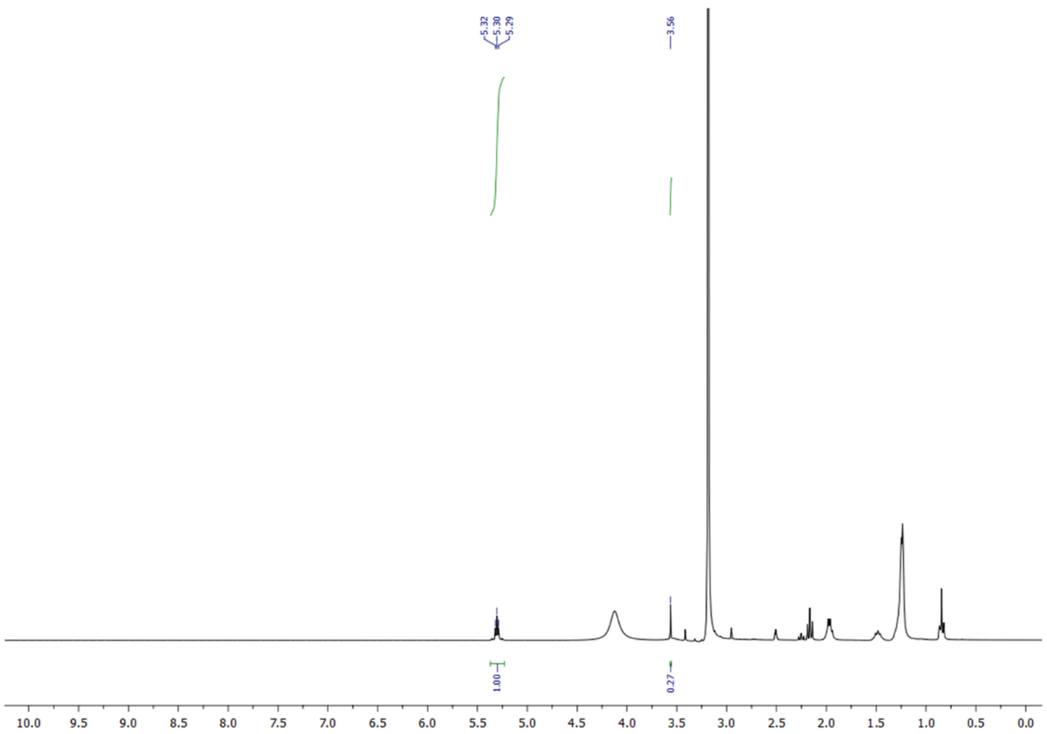

Figure S47. ^{1}H-NMR spectra (300 MHz, DMSO-*d*6) of the reaction mixture between oleic acid and methanol without catalyst at 100 °C for a duration of 12 hours.

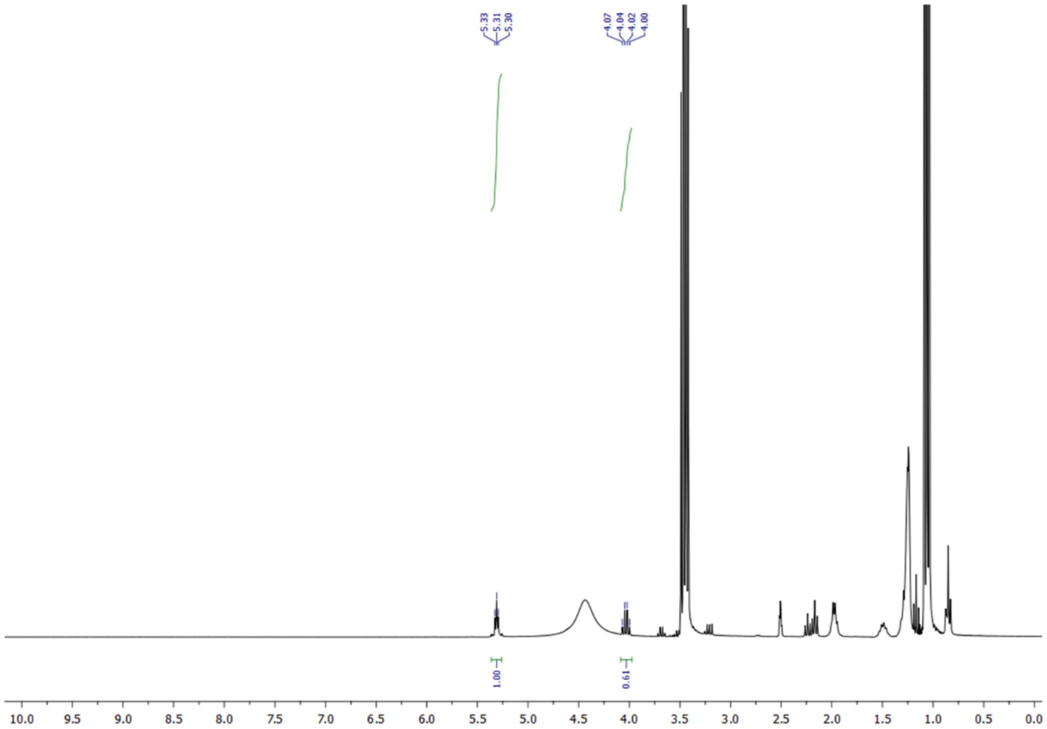

Figure S48. ^{1}H-NMR spectra (300 MHz, DMSO-*d*6) of the reaction mixture between oleic acid and ethanol catalysed by BCN-22 at 100 °C for a duration of 12 hours.

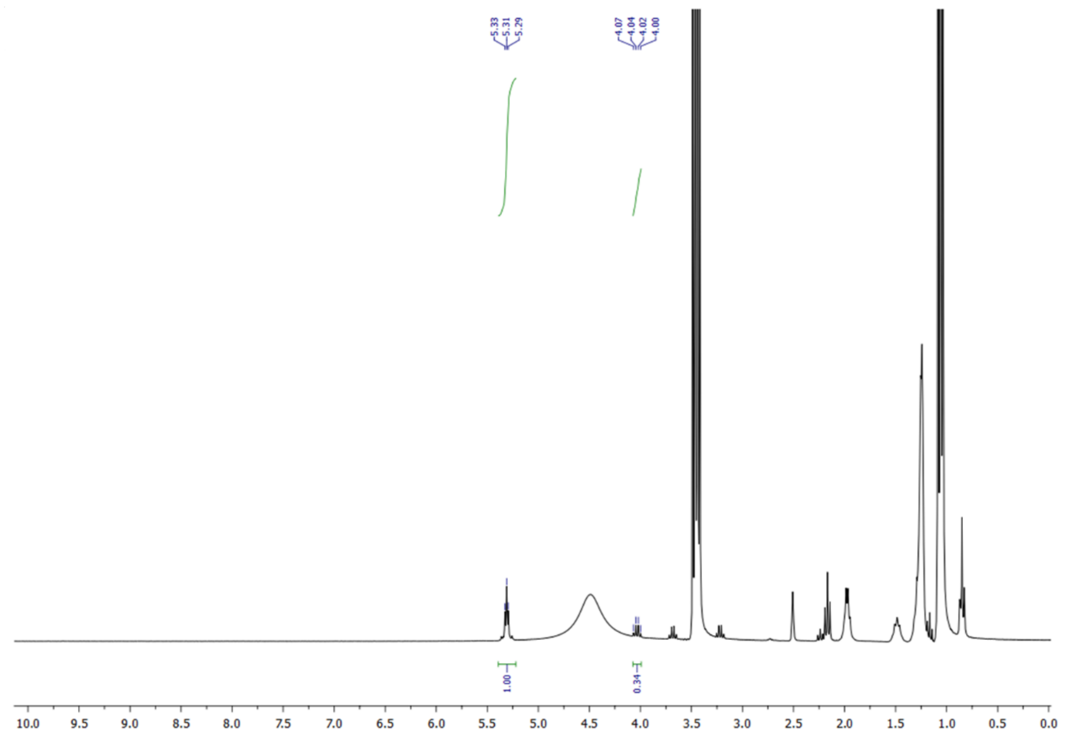

Figure S49. ^{1}H-NMR spectra (300 MHz, DMSO-d_6) of the reaction mixture between oleic acid and ethanol catalysed by LIMF-66W at 100 °C for a duration of 12 hours.

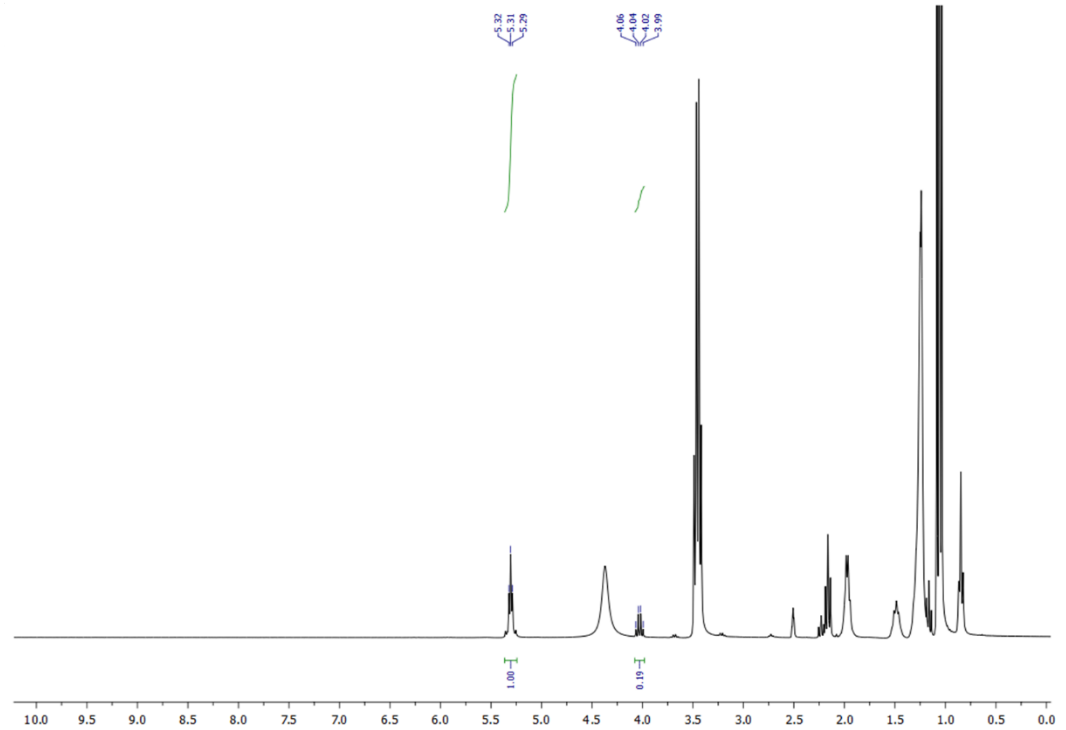

Figure S50. ^{1}H-NMR spectra (300 MHz, DMSO-d_6) of the reaction mixture between oleic acid and ethanol without catalyst at 100 °C for a duration of 12 hours.

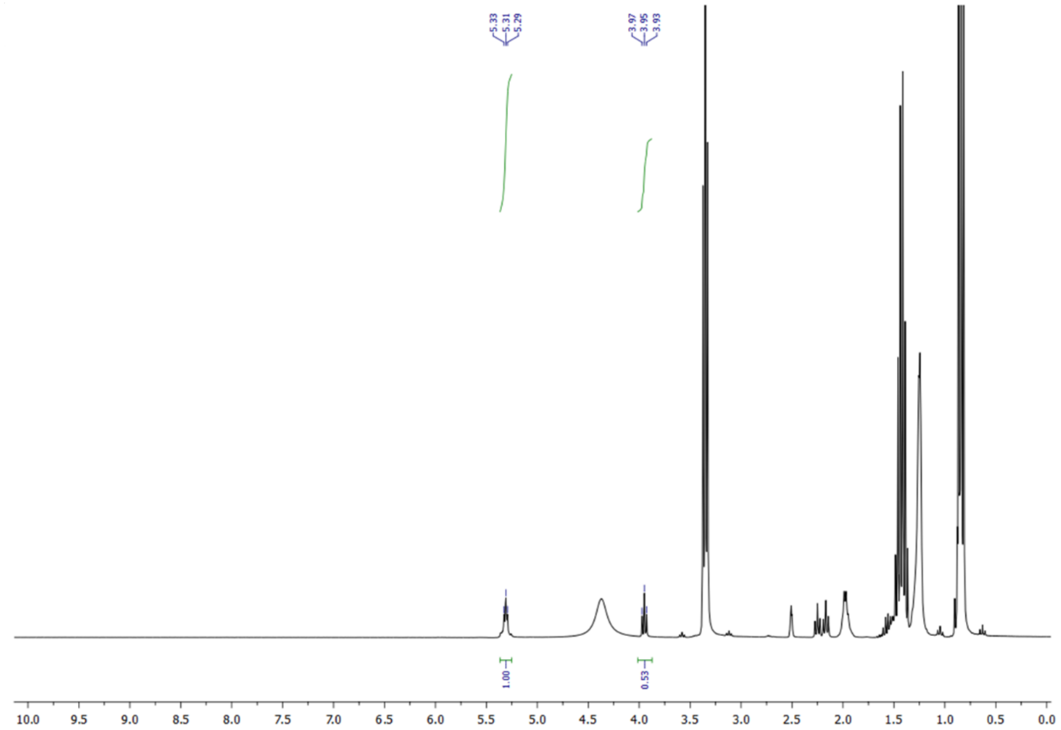

Figure S51. ^{1}H-NMR spectra (300 MHz, DMSO-d_6) of the reaction mixture between oleic acid and 1-propanol catalysed by BCN-22 at 100 °C for a duration of 12 hours.

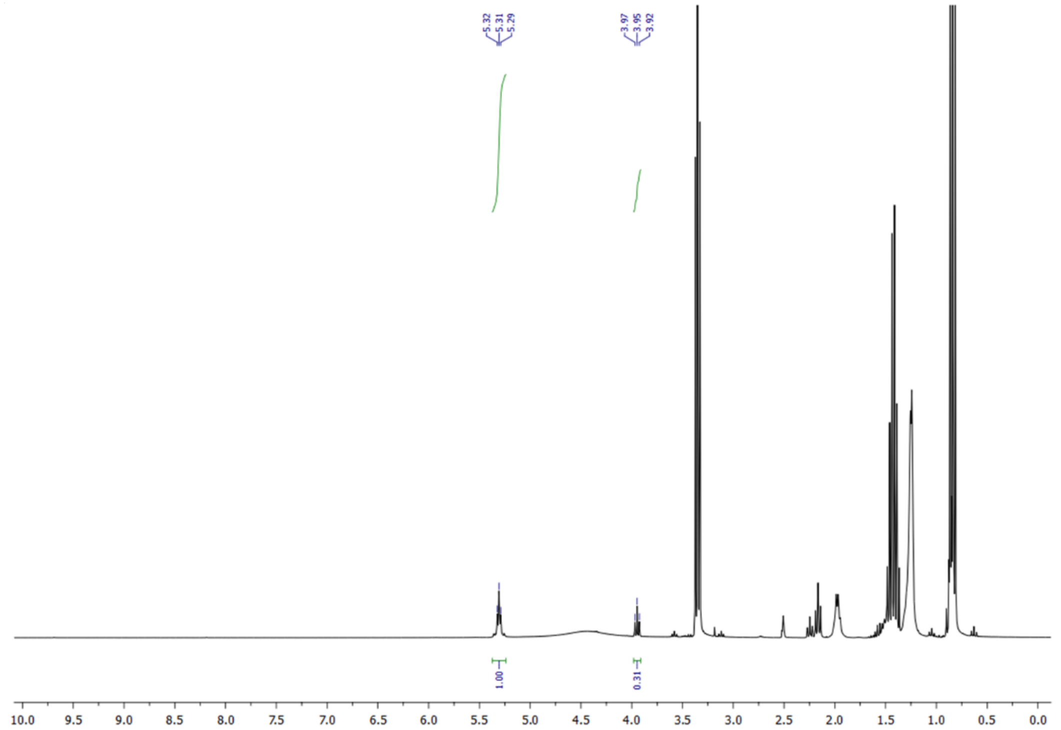

Figure S52. ^{1}H-NMR spectra (300 MHz, DMSO-d_6) of the reaction mixture between oleic acid and 1-propanol catalysed by LIMF-66W at 100 °C for a duration of 12 hours.

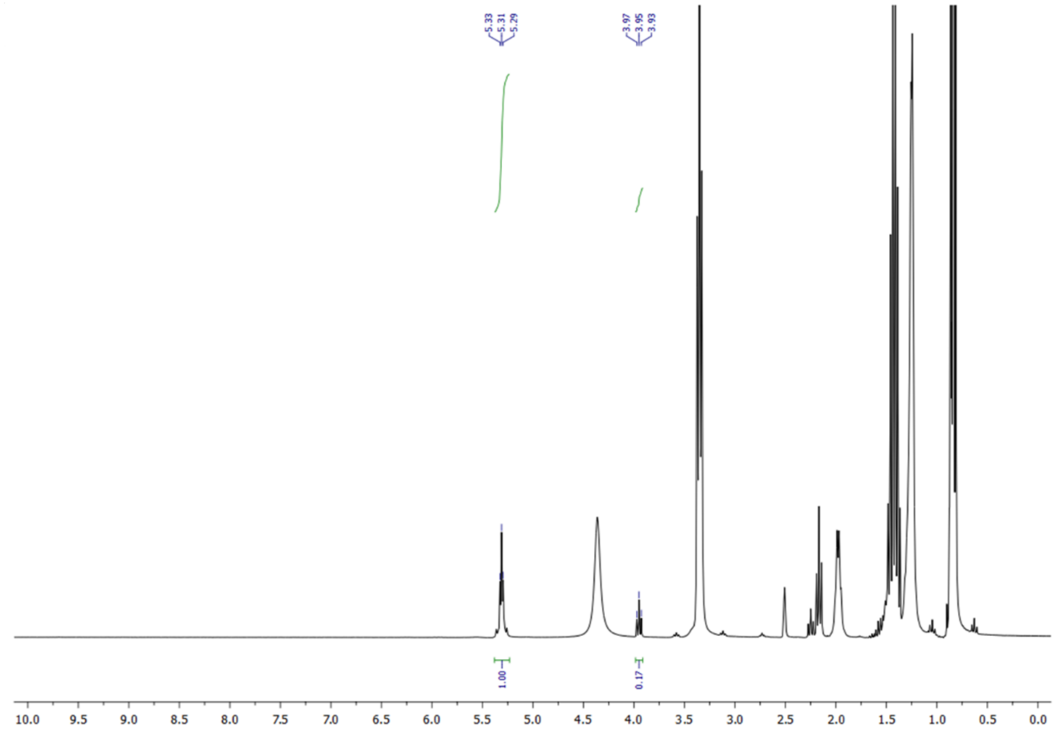

Figure S53. ^{1}H-NMR spectra (300 MHz, DMSO-d_6) of the reaction mixture between oleic acid and 1-propanol without catalyst at 100 °C for a duration of 12 hours.

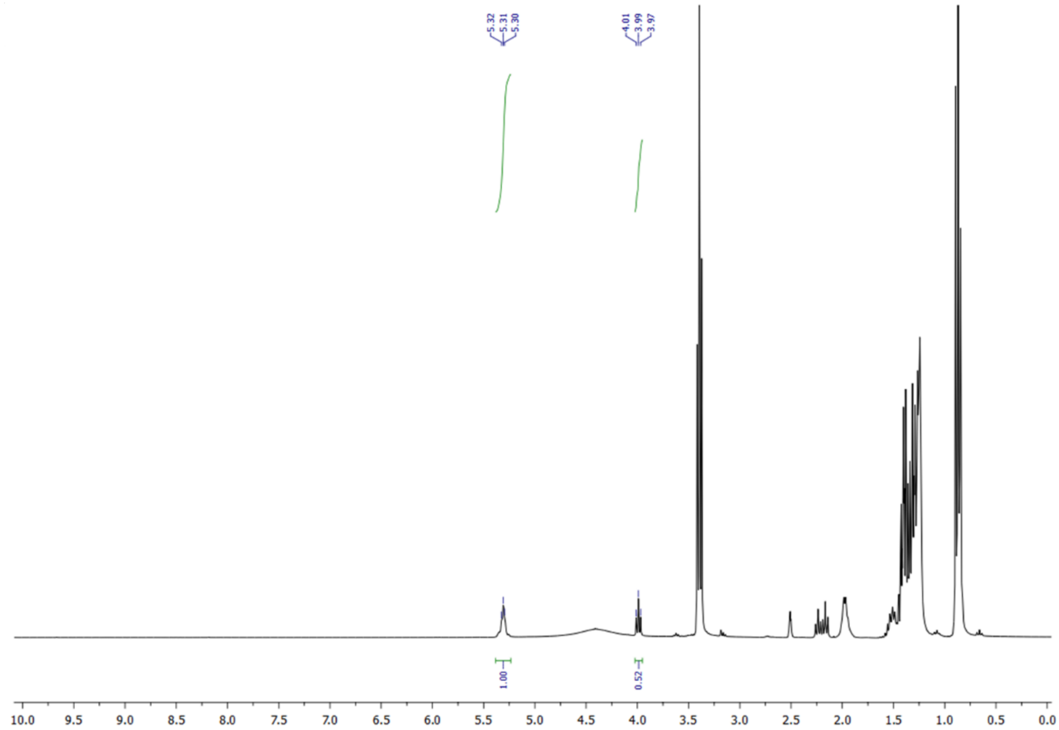

Figure S54. ^{1}H-NMR spectra (300 MHz, DMSO-d_6) of the reaction mixture between oleic acid and 1-butanol catalysed by BCN-22 at 100 °C for a duration of 12 hours.

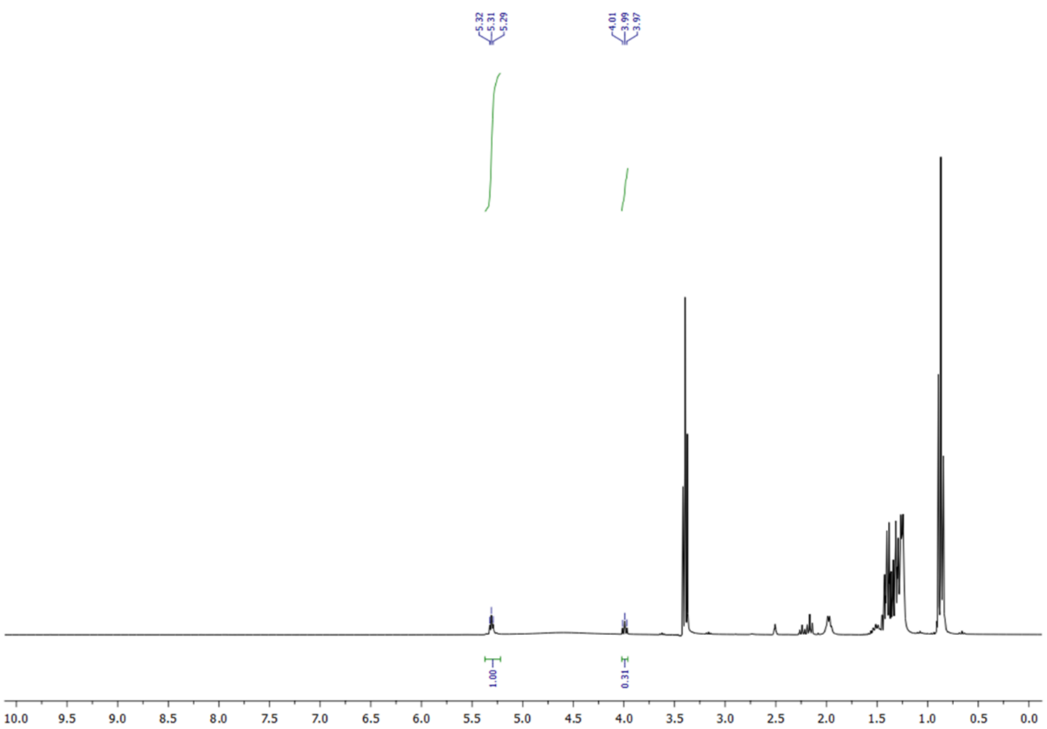

Figure S55. ^{1}H-NMR spectra (300 MHz, DMSO-*d*6) of the reaction mixture between oleic acid and 1-butanol catalysed by LIMF-66W at 100 °C for a duration of 12 hours.

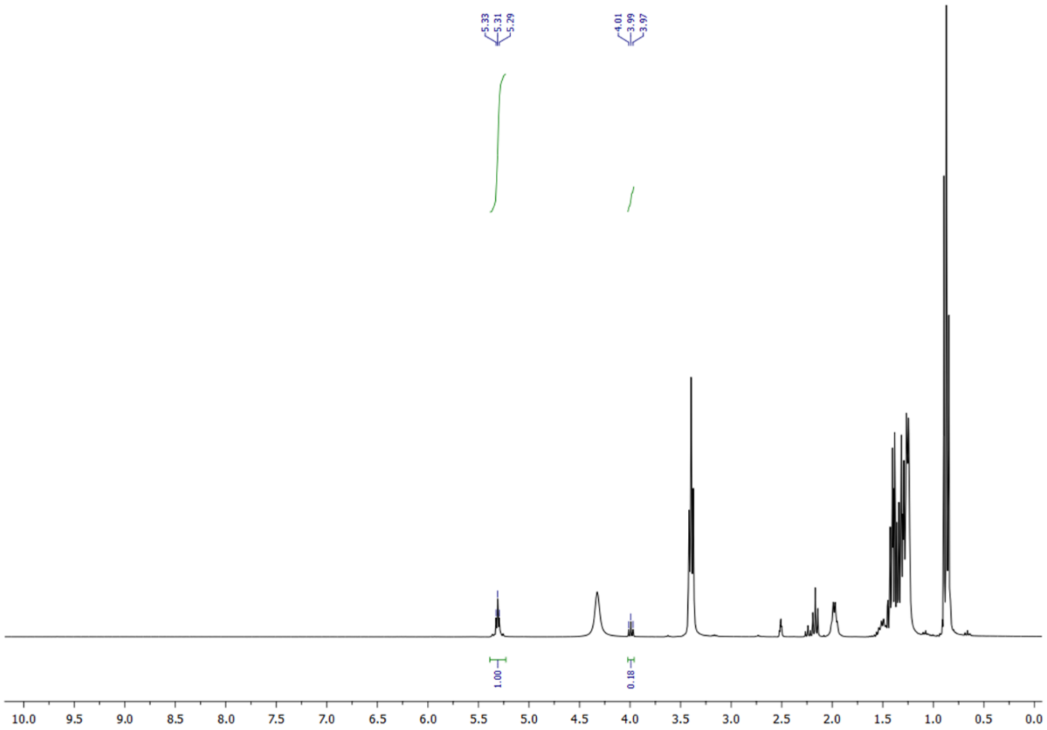

Figure S56. ^{1}H-NMR spectra (300 MHz, DMSO-*d*6) of the reaction mixture between oleic acid and 1-butanol without catalyst at 100 °C for a duration of 12 hours.

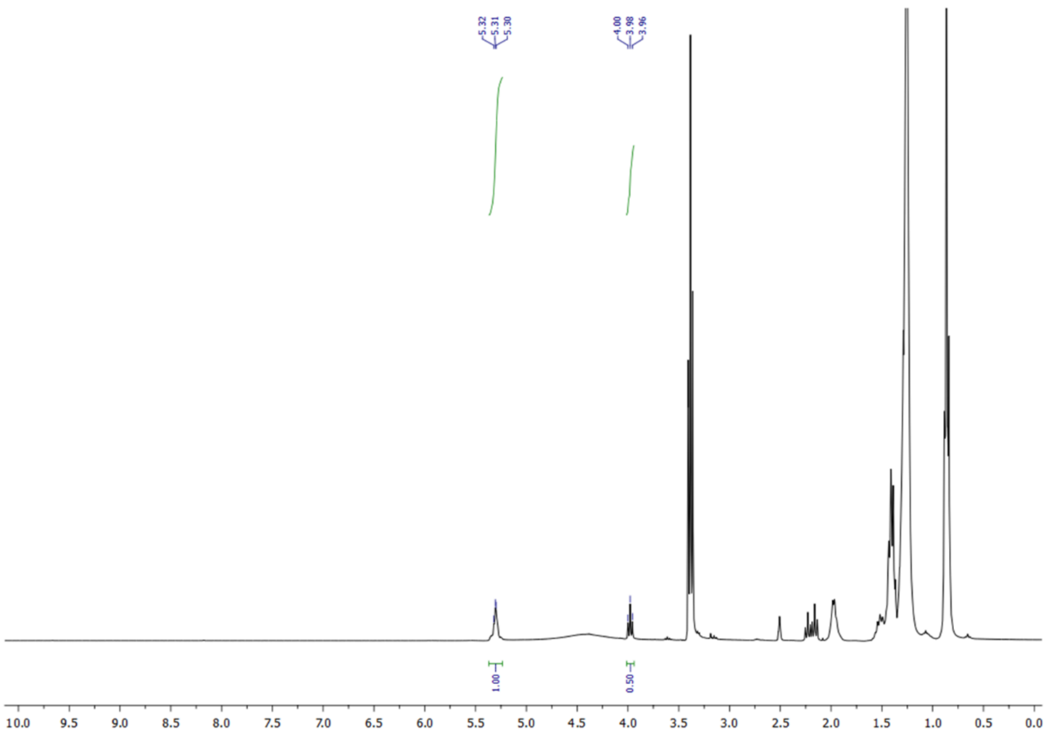

Figure S57. ^{1}H-NMR spectra (300 MHz, DMSO-d_6) of the reaction mixture between oleic acid and 1-hexanol catalysed by BCN-22 at 100 °C for a duration of 12 hours.

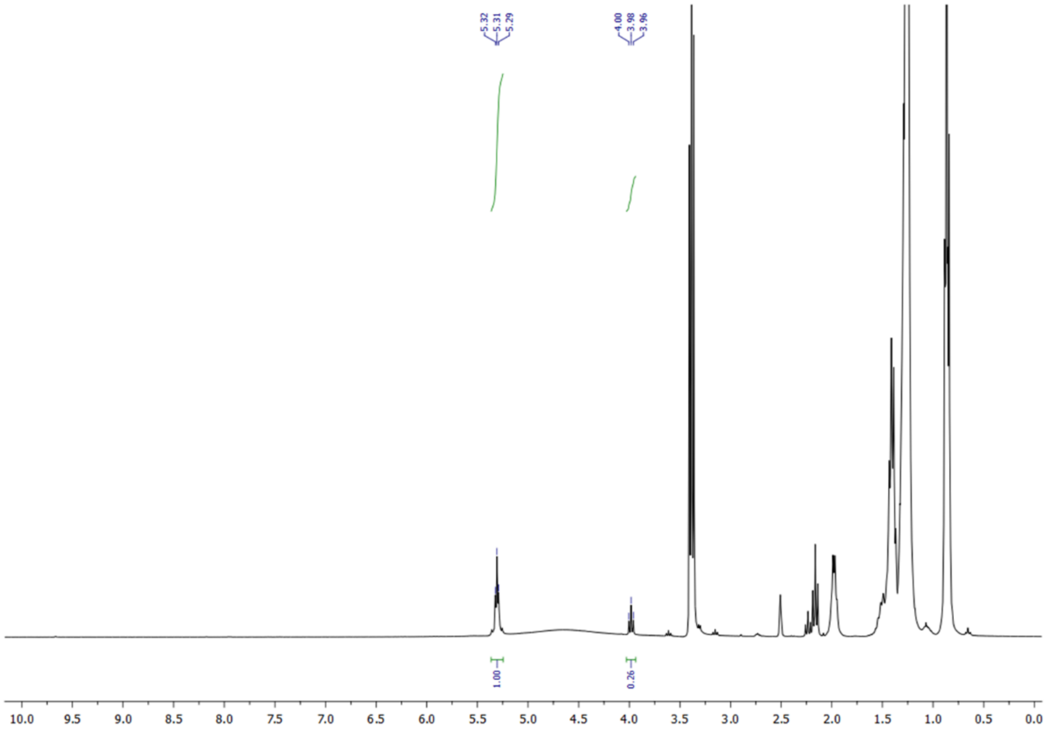

Figure S58. ^{1}H-NMR spectra (300 MHz, DMSO-d_6) of the reaction mixture between oleic acid and 1-hexanol catalysed by LIMF-66W at 100 °C for a duration of 12 hours.

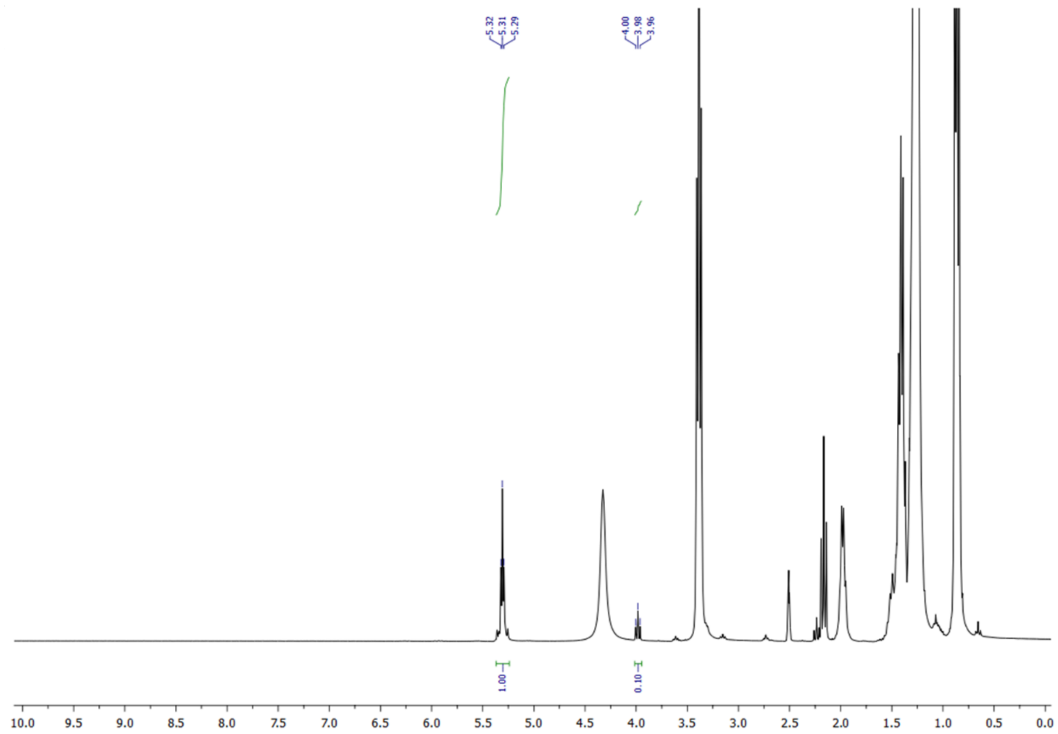

Figure S59. ^{1}H-NMR spectra (300 MHz, DMSO-d_6) of the reaction mixture between oleic acid and 1-hexanol without catalyst at 100 °C for a duration of 12 hours.

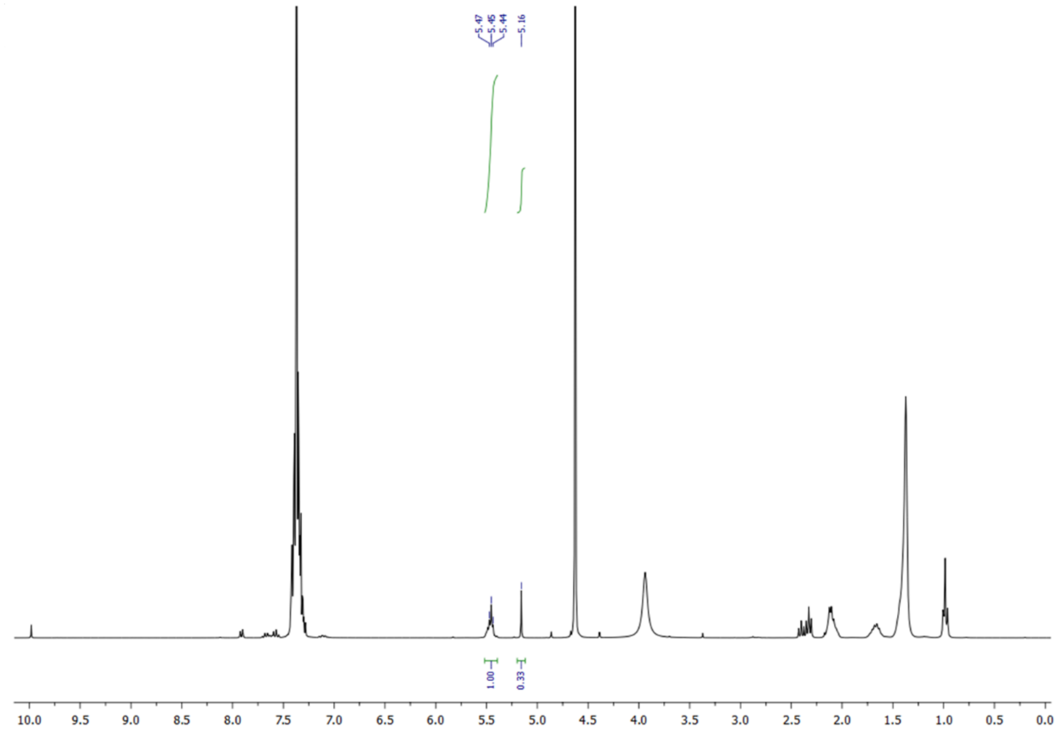

Figure S60. ^{1}H-NMR spectra (300 MHz, $CDCl_3$) of the reaction mixture between oleic acid and benzyl alcohol catalysed by BCN-22 at 100 °C for a duration of 12 hours.

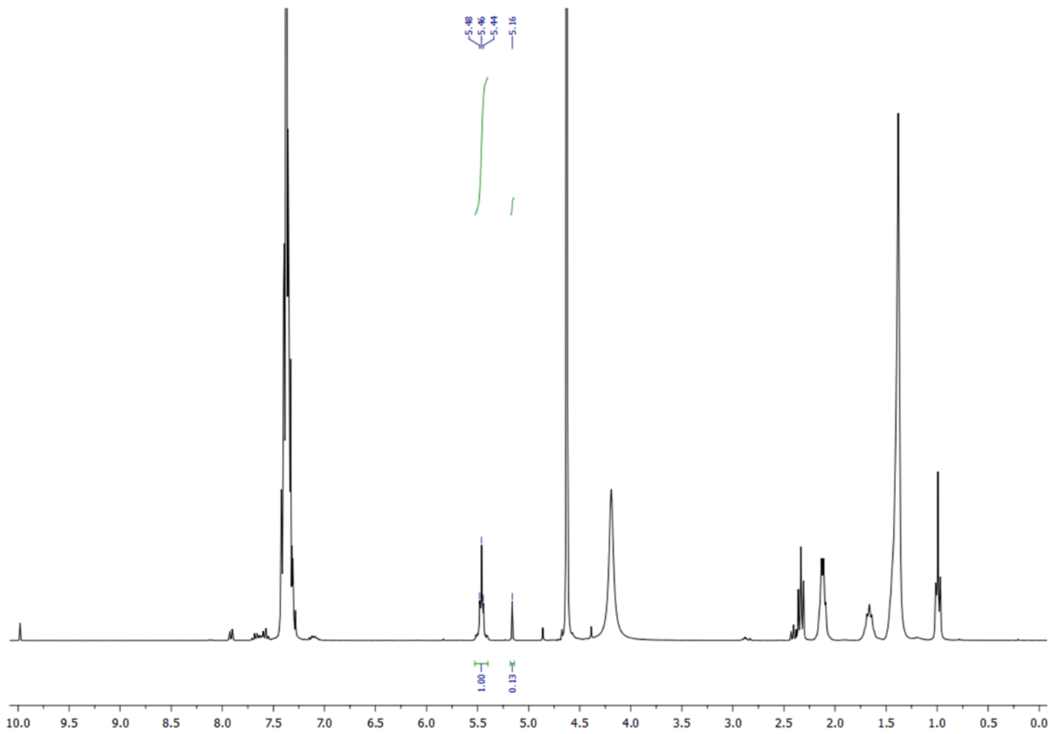

Figure S61. ^{1}H-NMR spectra (300 MHz, $CDCl_3$) of the reaction mixture between oleic acid and benzyl alcohol catalysed by LIMF-66W at 100 °C for a duration of 12 hours.

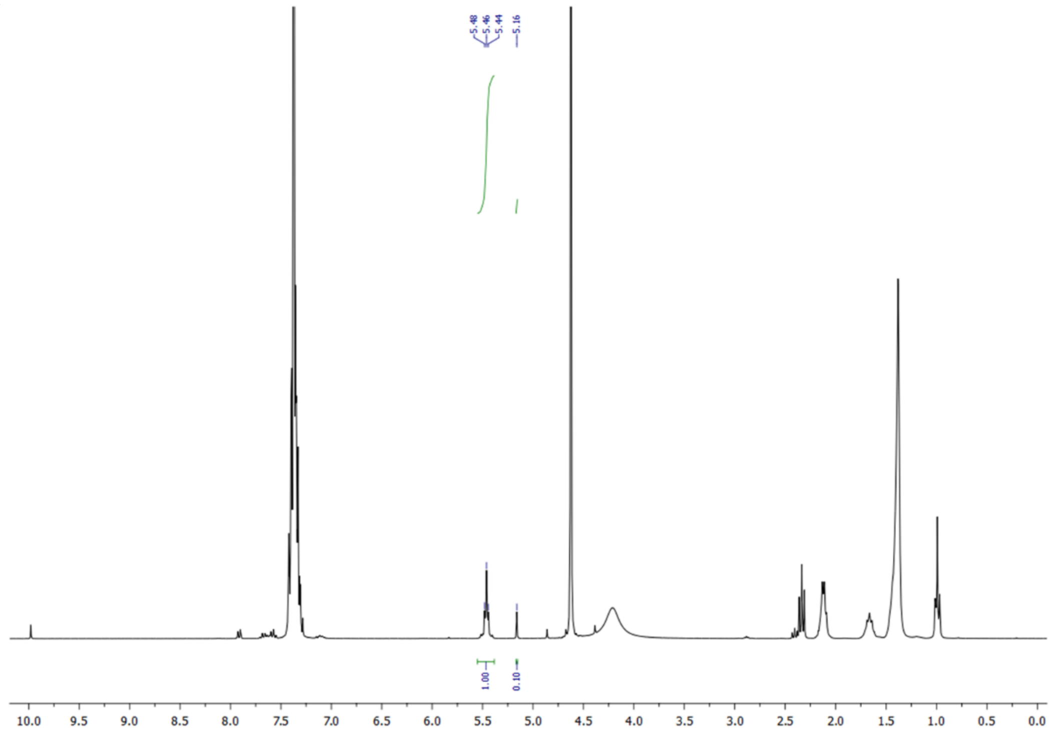

Figure S62. ^{1}H-NMR spectra (300 MHz, $CDCl_3$) of the reaction mixture between oleic acid and benzyl alcohol without catalyst at 100 °C for a duration of 12 hours.

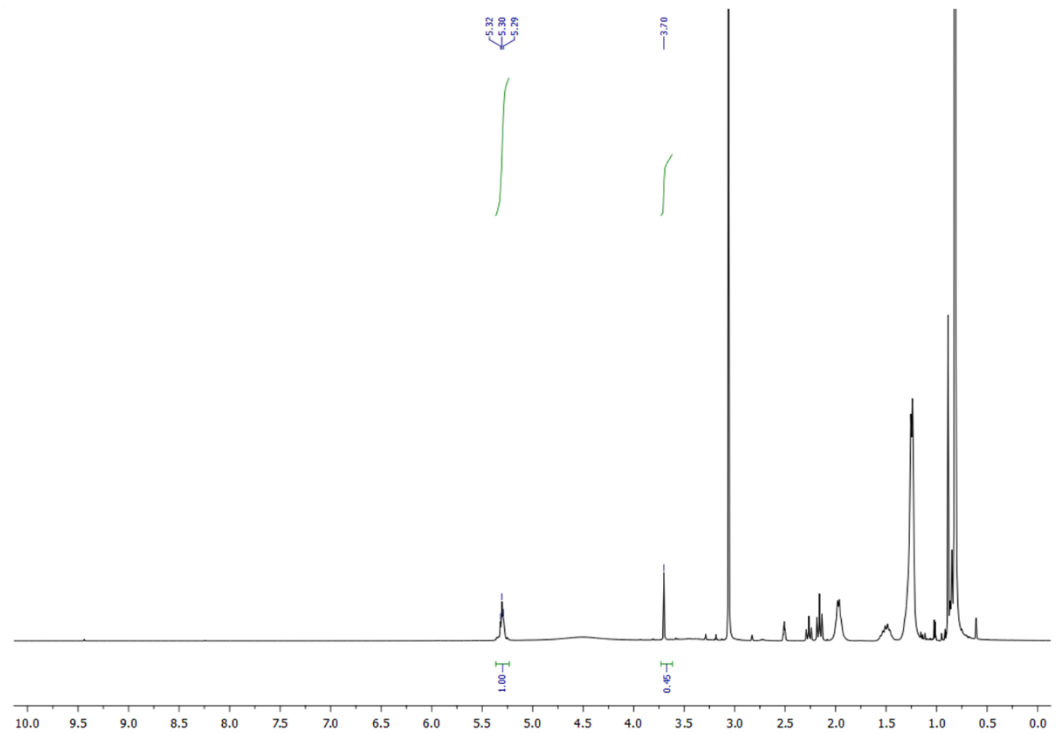

Figure S63. ^{1}H-NMR spectra (300 MHz, DMSO-d_6) of the reaction mixture between oleic acid and 2,2-dimethyl-1-propanol catalysed by BCN-22 at 100 °C for a duration of 12 hours.

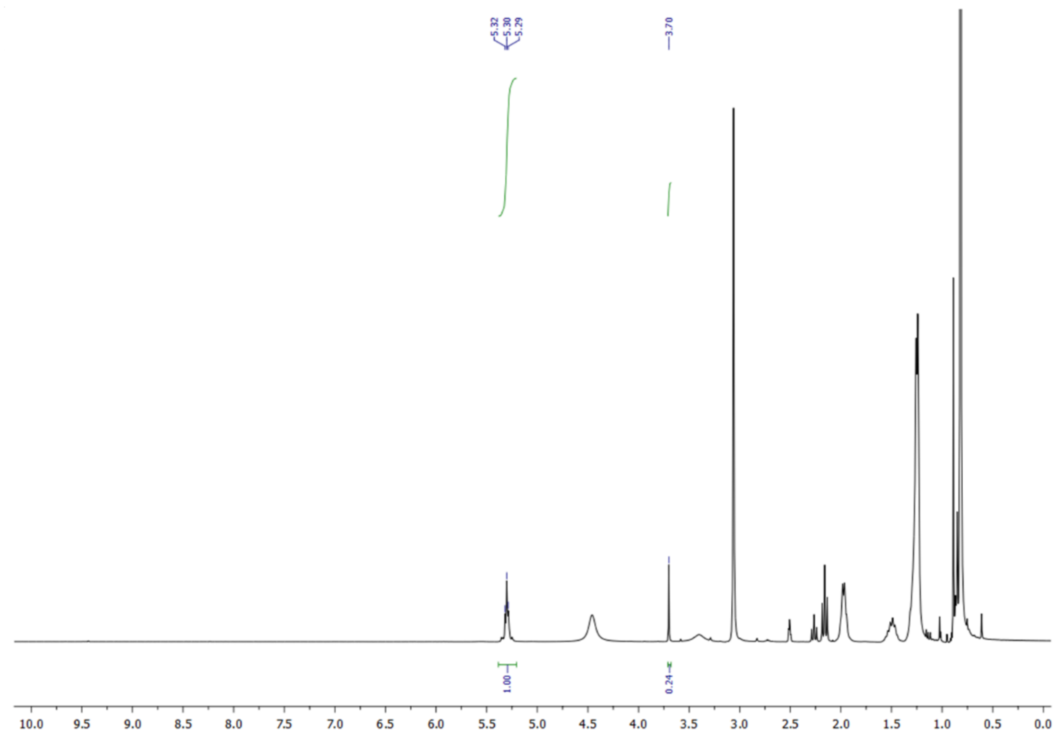

Figure S64. ^{1}H-NMR spectra (300 MHz, DMSO-d_6) of the reaction mixture between oleic acid and 2,2-dimethyl-1-propanol catalysed by LIMF-66W at 100 °C for a duration of 12 hours.

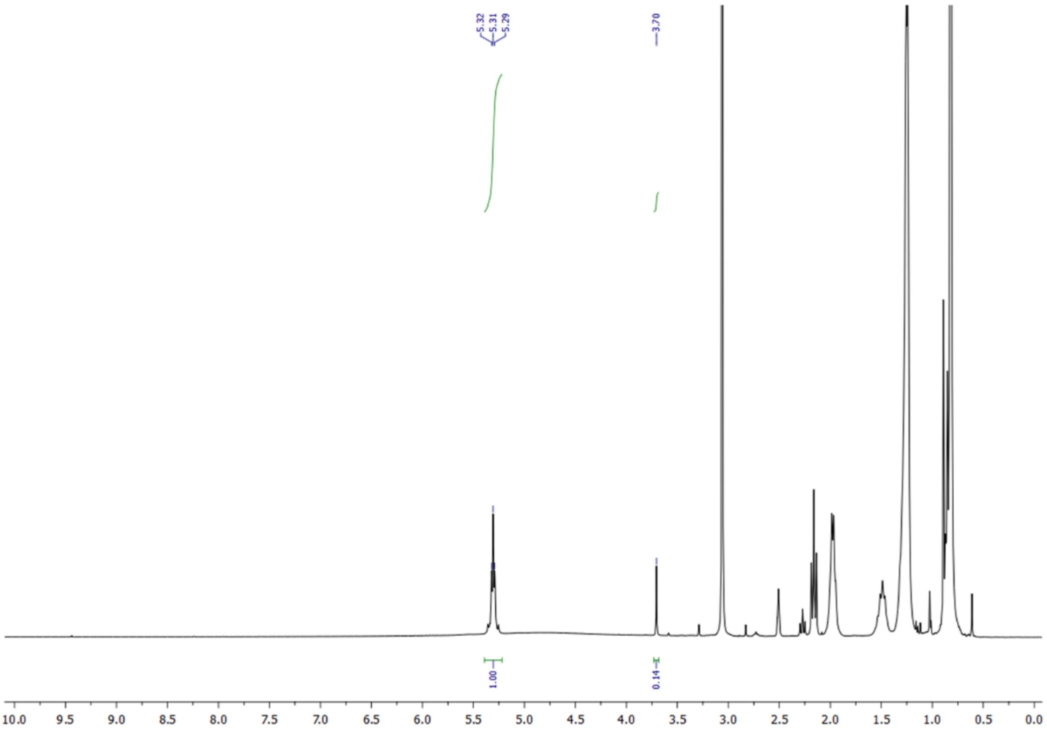

Figure S65. ^{1}H-NMR spectra (300 MHz, DMSO-d_6) of the reaction mixture between oleic acid and 2,2-dimethyl-1-propanol without catalyst at 100 °C for a duration of 12 hours.

Table S2. Catalytic esterification of oleic acid with different alcohols (reaction duration: 12 hours)

Alcohols	BCN-22		LIMF-66W		Blank
	% yield	TON	% yield	TON	% yield
Methanol	98	49	47	23	27
Ethanol	61	31	34	17	19
1-propanol	53	27	31	16	17
1-butanol	52	26	31	15	18
1-hexanol	50	25	26	13	10
Benzyl alcohol	33	17	13	6	10
2,2-dimethyl-1-propanol	45	23	24	12	14

S7.2. Recyclability experiments

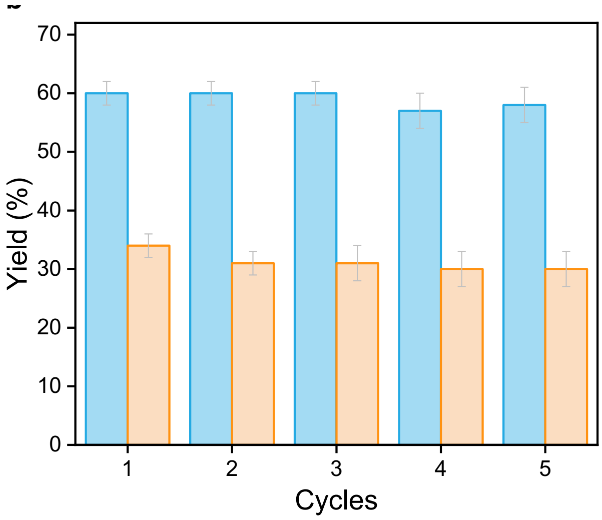

Figure S66. Graphical representation of the yield (in %) per catalytic run for the conversion of oleic acid to ethyl oleate using BCN-22 (blue) and LIMF-66W (orange) at 100 °C (time: 12 hours).

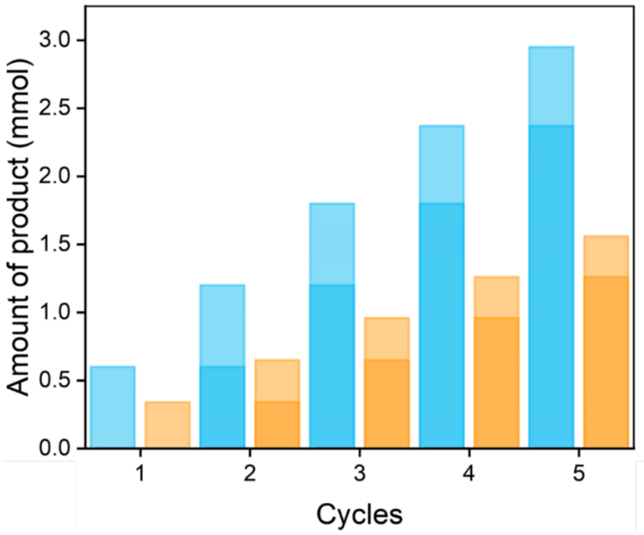

Figure S67. Graphical representation for the evolution of the cumulative yield (in mmol) per catalytic run for the conversion of oleic acid to ethyl oleate using BCN-22 (blue) and LIMF-66W (orange) at 100 °C (time: 12 hours).

S7.3. PXRD after catalytic experiments

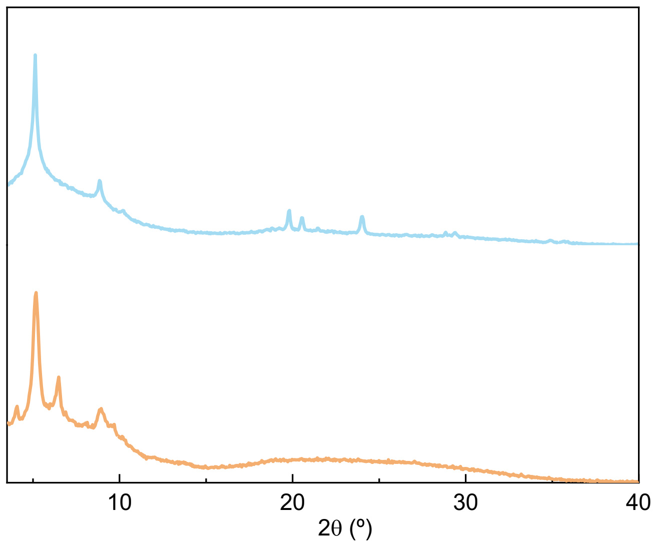

Figure S68. PXRD pattern of LIMF-66W (orange) and BCN-22 (blue) recovered from the catalytic experiments.

S7.4. ICP-OES

Table S3. ICP-OES measurements of LIMF-66W and BCN-22 before and after catalytic experiments.

Sample	wt% of Zr
LIMF-66W before catalysis	14.4
BCN-22 before catalysis	15.8
LIMF-66W after catalysis	14.4
BCN-22 after catalysis	15.2

S7.5. Hot-filtration experiments

Hot-filtration experiments were performed for the esterification of oleic acid (1 mmol) and ethanol (8 mmol), catalysed by either LIMF-66W or BCN-22 nanosheets (2.0 mol% of Zr) in the presence of 3 Å molecular sieves. After 3 hours of reaction at 100 ºC, the mixture was passed through a syringe filter to separate the solid catalyst. The reaction was then allowed to proceed up to 12 hours, following the general catalytic protocol (S7.1. General protocol), this time in the absence of the solid catalyst to check whether any active Zr species had leached into the reaction medium. The final reaction mixture was analyzed by ^{1}H-NMR spectroscopy to quantify the yield.

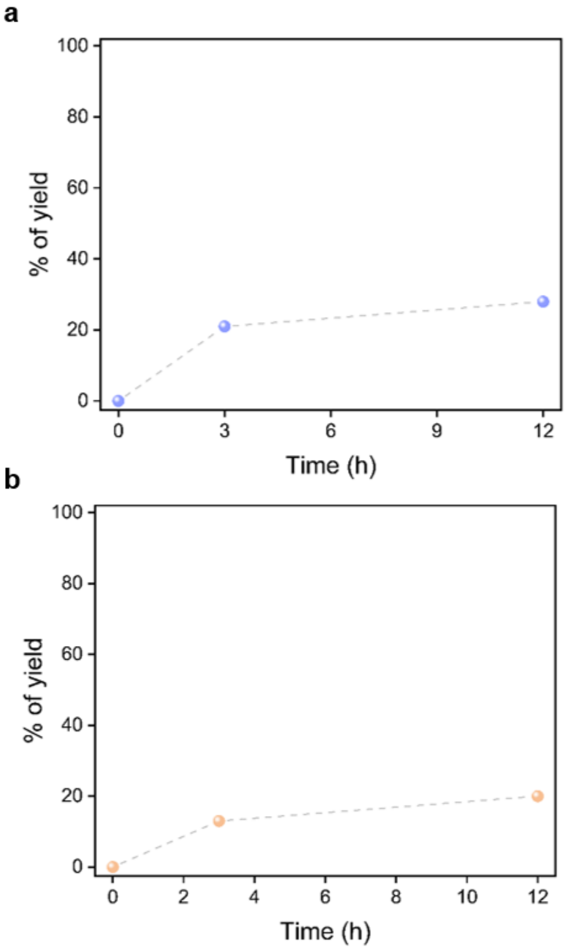

Figure S69. Hot filtration tests for the reaction between oleic acid and ethanol at 100 °C with (a) BCN-22 and (b) LIMF-66W. (Time duration of 12 hours and the catalyst was filtered at 3 hours).

References

1. Juanhuix, J.; Gil-Ortiz, F.; Cuní, G.; Colldelram, C.; Nicolás, J.; Lidón, J.; Boter, E.; Ruget, C.; Ferrer, S.; Benach, J. Developments in optics and performance at BL13-XALOC, the macromolecular crystallography beamline at the ALBA synchrotron. *J. Synchrotron Radiat.* **2014**, *21*, 679-689.
2. Hu, Z.; Huang, G.; Lustig, W. P.; Wang, F.; Wang, H.; Teat, S. J.; Banerjee, D.; Zhang, D.; Li, J. Achieving exceptionally high luminescence quantum efficiency by immobilizing an AIE molecular chromophore into a metal–organic framework. *Chem Comm.* **2015**, *51*, 3045-3048.
3. Chen, C.-X.; Wei, Z.-W.; Fan, Y.-N.; Su, P-Y.; Ai, Y.-Y.; Qui, Q.-F.; Wu, K.; Yin, S.-Y.; Pan, M.; Su, C.-Y. Visualization of Anisotropic and Stepwise Piezofluorochromism in an MOF Single Crystal. *Chem.* **2018**, *4*, 11, 2658-2669.
4. Wang, Y.; Li, L.; Yan, L.; Gu, X.; Dai, P.; Liu, D.; Bell, J. G.; Zhao, G.; Zhao, X.; Thomas, K. M. Bottom-Up Fabrication of Ultrathin 2D Zr Metal–Organic Framework Nanosheets through a Facile Continuous Microdroplet Flow Reaction. *Chem. Mater.* **2018**, 30, 9, 3048–3059.
5. Yu Y, Feng X, Xie Z, Wu Y, Xi H. 2D MOF with multifunctionalized catalytic sites for one-pot tandem synthesis of cyclic carbonates from olefins and CO_2. *AIChE J.* **2024**; 70(2): e18290.
6. Zhan, D.; Yu, Z.; Saeed, A.; Hu, Q.; Zhao, N.; Xu, W.; Wang, J.; Kong, L.; Liu, J. Molecular insights into the sensitivity detection mechanism of fluorescent 2D Zr-BTB for 2,4-dinitrophenol. *J. Mater. Chem. C*, **2023**,11, 10738-10747.
